# Genome-Wide Association Analyses of Equine Metabolic Syndrome Phenotypes in Welsh Ponies and Morgan Horses

**DOI:** 10.3390/genes10110893

**Published:** 2019-11-06

**Authors:** Elaine Norton, Nichol Schultz, Ray Geor, Dianne McFarlane, James Mickelson, Molly McCue

**Affiliations:** 1 Veterinary Population Medicine Department, University of Minnesota, St. Paul, MN 55108, USA; mccar556@umn.edu (N.S.); mccu0173@umn.edu (M.M.); 2 College of Sciences, Massey University, Palmerston North 4442, New Zealand; 3 Department of Physiological Sciences, Oklahoma State University, Stillwater, OK 74078, USA; diannem@okstate.edu; 4 Veterinary Biomedical Sciences Department, University of Minnesota; St. Paul, MN 55108, USA; micke001@umn.edu

**Keywords:** EMS, insulin dysregulation, fat metabolism, genetics, genetic risk factors, horses, genome-wide association analysis

## Abstract

Equine metabolic syndrome (EMS) is a complex trait for which few genetic studies have been published. Our study objectives were to perform within breed genome-wide association analyses (GWA) to identify associated loci in two high-risk breeds, coupled with meta-analysis to identify shared and unique loci between breeds. GWA for 12 EMS traits identified 303 and 142 associated genomic regions in 264 Welsh ponies and 286 Morgan horses, respectively. Meta-analysis demonstrated that 65 GWA regions were shared across breeds. Region boundaries were defined based on a fixed-size or the breakdown of linkage disequilibrium, and prioritized if they were: shared between breeds or across traits (high priority), identified in a single GWA cohort (medium priority), or shared across traits with no SNPs reaching genome-wide significance (low priority), resulting in 56 high, 26 medium, and seven low priority regions including 1853 candidate genes in the Welsh ponies; and 39 high, eight medium, and nine low priority regions including 1167 candidate genes in the Morgans. The prioritized regions contained protein-coding genes which were functionally enriched for pathways associated with inflammation, glucose metabolism, or lipid metabolism. These data demonstrate that EMS is a polygenic trait with breed-specific risk alleles as well as those shared across breeds.

## 1. Introduction

Equine metabolic syndrome (EMS) is now best described as a clustering of risk factors often leading to laminitis, which is the primary clinical concern due to the painful and often career ending outcome of this disease [1]. Although laminitis itself is not fatal, in the best interest of the patient, the severity and crippling pain often leads to a decision of euthanasia [2]. The key component of EMS is insulin dysregulation, which are derangements in the balanced relationship between plasma insulin, glucose and lipids, and manifests clinically as baseline hyperinsulinemia, an exaggerated or prolonged insulin or glucose response post oral or intravenous carbohydrate challenge, tissue insulin resistance, or hypertriglyceridemia [1]. 

Although a dominant mode of inheritance for laminitis status was originally proposed for a small group of ponies [3], breed differences in EMS susceptibility, metabolic profiles, and clinical severity have led to the more widely applicable, alternative hypothesis that EMS is a complex disease, with both environmental and genetic risk factors contributing to disease severity. As a complex trait, it is likely that EMS is the result of a combination of genes with variable modes of inheritance, penetrance and effect size [4]. Recently, our laboratory provided evidence for this hypothesis through estimation of narrow sense heritability in a cohort of Morgan horses and Welsh ponies, where eight metabolic measurements were estimated to have low, moderate or high heritability [5]. Further, several heritability estimates varied across the two breeds, providing further evidence for breed related differences and are consistent with heritability estimates across ethnic groups for metabolic syndrome (MetS) in humans, a syndrome with shares distinct metabolic similarities to EMS.

Although heritability estimates provide valuable insight into the genetic contribution to a trait, they do not provide information on the number of contributing genes, specific genes involved, or where in the genome these genes are located. Identification of the coding and non-coding variants contributing to a complex trait are important for understanding its complete pathophysiology and to gain a better understanding of how genes interact or are influenced by the environment. Further, this information is necessary for the development of genetic tests which would allow veterinarians to assess a patient’s risk for developing EMS before they show clinical signs, identify horses that need frequent monitoring or early environmental modifications, and provide responsible breeding recommendations.

Genome-wide association analyses (GWA) has been used and validated across multiple species for both simple and complex traits to narrow down the genome to specific regions of interest harboring the risk alleles and to provide valuable information about the genetic architecture of a trait. For example, GWA for MetS have led to identification of quantitative trait loci harboring candidate genes in several metabolic pathways, including alleles influencing lipoprotein particle size and glucose, insulin and lipid homeostasis [6,7]. Further, these studies have identified different risk alleles amongst ethnic groups [8].

We hypothesized that major genetic risk factors leading to EMS are shared across breeds, and that differences in the severity and secondary features of the EMS phenotype between breeds, or between individuals within a breed, are the result of modifying genetic risk alleles with variable frequencies between breeds. The objectives of this study were to perform within breed GWA to identify significant contributing loci in Welsh ponies and Morgan horses, two breeds known to be high risk for EMS, and to use meta-analysis to identify shared and unique loci between both breeds. We further prioritized regions identified on GWA and performed a functional enrichment analysis on protein-coding genes within the prioritized regions. 

## 2. Materials and Methods 

### 2.1. Samples

Horses used in this study were a part of a large across-breeds study evaluating the EMS phenotype [9]. From this dataset, 264 Welsh ponies (194 females and 70 males with a mean age of 11.7 years) and 287 Morgan horses (184 females and 102 males with a mean age of 12.3 years) were included in this analysis. The Welsh pony cohort represented 74 section As, 146 section Bs, three section Cs, 15 section Ds, 19 section Hs, seven sections Ps, and 10 unknown/unregistered Welsh ponies (Appendix A). Samples were collected from 31 and 28 farms for the Morgan horses and Welsh ponies, respectively, throughout the United States and Canada.

Sample procedures and protocols have been described elsewhere [5,9,10]. Briefly, phenotype data collected on all horses included signalment, medical history, laminitis status, environmental management (feed, supplements, turnout and exercise regimen), and morphometric measurements (body condition score (BCS), wither height, and neck and girth circumference). After an eight hour fast, an oral sugar test (OST) was performed using 0.15 mg/kg Karo lite corn syrup as previously described [11]. Biochemical measurements at baseline included insulin (TKIN1 Insulin Coat-A-Count Kit, Siemens Healthcare Diagnostics Inc., Los Angeles, CA, USA), glucose (YSI 2300 STAT Plus glucose and lactate analyzer, YSI Inc., Yellow Springs, OH, USA), non-esterified fatty acids (NEFA; Wako HR Series NEFA-HR kit, Wako Pure Chemical Industries, Ltd., Chuo-Ku Osaka, Japan), triglycerides (TG; TR0100 Serum Triglyceride Determination kit, MilliporeSigma, Saint Louis, MO, USA), adiponectin (EZHMWA-64K Human High Molecular Weight Adiponectin ELISA, MilliporeSigma, Saint Louis, MO, USA), leptin (XL-85K Multi-Species Leptin RIA, MilliporeSigma, Saint Louis, MO, USA) and ACTH (LKAC1 ACTH kits, Siemens Healthcare Diagnostics Inc., Los Angeles, CA, USA). Biochemical measurements 75 minutes after the OST included insulin (INS-OST) and glucose (GLU-OST). 

Horses with a history or phenotypic appearance of pars pituitary intermedia dysfunction (PPID) were excluded from the study. A previously laminitic horse was defined as an individual who had been diagnosed with pasture-associated or endocrinopathic laminitis by a veterinarian, had radiographic evidence of laminitis, or had signs indicative of chronic laminitis observed by the researchers at the time of sampling. Horses in which laminitis could have been caused by another inciting factor (history of illness, grain-overload, corticosteroid administration or PPID), or who had clinically-evident, acute laminitis at the time of sampling, were excluded from the study.

### 2.2. Genotype Data

DNA was isolated from whole blood or hair roots using the Puregene Blood Core Kit, (Qiagen, Germantown, MD, USA) per the manufacturer’s instructions. Genome-wide single nucleotide polymorphism (SNP) genotyping was performed on all horses. Horses were genotyped either on the EquineSNP50 Genotyping BeadChip (Illumina, Inc., San Diego, CA, USA; 268 Morgan horses), Axiom Equine MCEc670 array (Thermo Fisher Scientific, Coon Rapids, MN, USA; 220 Welsh ponies), or Axiom Equine MCEc2M array (Thermo Fisher Scientific, Coon Rapids, MN, USA; 44 Welsh ponies and 43 Morgan horses), containing 54,602 SNPs, 670,795 SNPs, and 2,011,826 SNPs across the equine genome including the 31 autosomes and X chromosome, respectively.

Haplotype phasing and genotype imputation up to the ~two million SNPs present on the Axiom Equine MCEc2M array were performed on horses genotyped on the two lower density arrays using Beagle software [12]. Based on published recommendations [13], a cross breed population of 496 horses genotyped on the MCEc2M array, including the Welsh ponies and Morgan horses described above, were used as the reference population. Imputation concordance was determined to be 99.2% in the Morgan horses and 99.1% in the Welsh ponies. SNPs that did not have 100% concordance were removed from the data, yielding a total of 1,931,327 SNPs in the Welsh ponies and 1,932,766 SNPs in the Morgan horses.

Quality control on the imputed data was performed using the Plink software package [14]. All horses passed quality control, including evaluation for discordant sex information and SNP genotyping rate (>95%). Individual SNPs that had a genotyping rate of <90%, a minor allele frequency (MAF) of <1.0% or were outside of Hardy-Weinberg equilibrium (*p*-values < 1.0 × 10^−05^), were removed. After genotype pruning, 1,428,337 and 1,158,831 SNPs remained for subsequent analyses in the Welsh ponies and Morgan horses, respectively. Of these, a total 688,471 SNPs were shared between both breeds. Base pair positions for all SNPs were mapped to EquCab3 [15].

### 2.3. Genome-Wide Association Analyses (GWA)

Eleven traits significantly associated with EMS, including insulin, glucose, adiponectin, leptin, NEFA, TG, ACTH, INS-OST, GLU-OST, and measures of obesity (neck circumference to withers height ratio [NH] and girth circumference to withers height ratio [GH]), were treated as quantitative response variables in the GWA analyses. Laminitis status was treated as a binary response variable. All quantitative traits were tested for normality using a normal probability plot and Shapiro test and adjusted for normality as appropriate. Adiponectin, leptin, and NEFA were square root adjusted and insulin, INS-OST and triglycerides and ACTH were log transformed. Glucose, GLU-OST, and NH and GH ratios were normally distributed and did not need to be adjusted. 

Trait measurements were adjusted to account for known confounding covariates using the residuals from a linear mixed effects model in the R software program Linear and Nonlinear Mixed Effects Models (nlme), with sex and age included as fixed effects and farm as a random effect [16]. For each trait, within breed GWA were performed from the imputed SNP genotype data using a custom code for an improved mixed linear regression analysis [9]. This algorithm utilizes a three-step process, which combines a Bayesian Sparse Linear Mixed Model (BSLMM) [17] available in the software program Genome-wide Efficient Mixed Model Association (GEMMA) [18] and a linear mixed model implemented in FaST-LMM [19] (see Appendix B for a full description of this model). The threshold for genome-wide significance was based on the effective number of independent tests (SNPs not in linkage disequilibrium [LD]) as calculated by the Genetic Type 1 Error Calculator [20]. In the Welsh ponies, this value was 841,750 SNPs, resulting in a Bonferroni-corrected threshold for genome-wide significance of 5.98 × 10^−08^. For the Morgan horses, the effective number of independent tests was 657,030 SNPs, resulting in a Bonferroni corrected threshold for genome-wide significance of 7.61 × 10^−08^. The suggestive threshold for both breeds was set at 1.00 × 10^−05^ [21,22]. 

**Figure A1:**
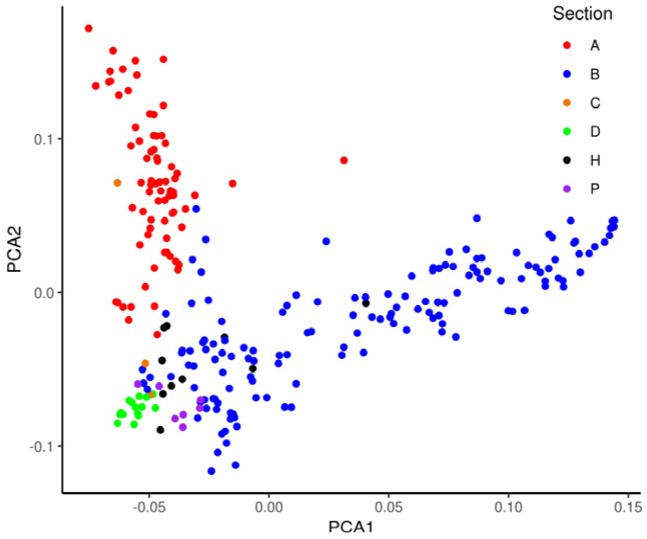
Principal components analysis for the Welsh ponies showing clustering between sections.

Principal components analysis (PCA) revealed population stratification in the Welsh pony cohort based on clustering of the registered sections (Figure A1). To account for this population substructure, and avoid over-fitting the model, three separate GWA were performed using the full cohort (*n* = 264), sections A, B, C and D (*n* = 238) and sections A and B (*n* = 220). In order to maximize sensitivity, the union of the GWA results from all three cohorts was used (Appendix A).

### 2.4. Meta-analysis

A GWA meta-analysis was performed with the software program METASOFT [23] using the Morgan horse and Welsh pony GWA summary data from the 688,471 SNPs that were shared between breeds. Briefly, the METASOFT algorithm uses a random effects model which adjusts for heterogeneity between studies by allowing the effect size of the alternative allele to vary between populations. Unlike other random effects models, where both the null and alternative models assume heterogeneity, METASOFT uses a likelihood ratio test that assumes heterogeneity only under the alternative model [24]. The effective number of shared SNPs was 306,023 in the Morgan horses and 307,349 in the Welsh ponies as calculated by GEC. For a more conservative p-value, the threshold for genome-wide significance was determined using the effective number of SNPs for the Welsh ponies (0.05/307,349) and set at 1.63 × 10^−07^. The suggestive threshold was set at 1.00 × 10^−05^ [21,22]. To be considered a region of interest identified on meta-analysis (MA-ROI), at least one SNP needed to exceed the threshold for genome-wide significance.

### 2.5. Prioritization of GWA Regions and Identification of Positional Candidate Genes

All GWA regions where SNPs exceeded the suggestive threshold for significance were reviewed. To be considered within a single region, consecutive SNPs on the same chromosome had to be within 500 kb of each other [24,25]. Regions of interest had to contain a minimum of five SNPs exceeding the suggestive threshold, with at least one SNP exceeding the threshold for genome-wide significance.

#### 2.5.1. Fixed-Size Regions

The boundaries of the fixed-size region were defined as 500 kb 5′ of the base pair position of the minimum SNP within the region and 500 kb 3′ of the base pair position of the maximum SNP [24,25,26,27,28,29]. A region was identified as shared if it was within the boundaries of another region and prioritized as described below.

#### 2.5.2. LD-Bound Regions

To define the boundaries of the LD-bound region, the software program Plink was utilized to calculate the pairwise LD measures for all SNPs within the region [14]. Window size was set at 1 Mb from the minimum and maximum SNP within the region; a pairwise calculation for LD with the test SNP was performed for all SNPs within the window. The threshold for SNPs within LD was set at an r^2^ of greater than 0.3 [27]. A custom code was used to identify regions where LD for all SNPs dropped below 0.3 and spanned at least 100 kb both 5′ and 3′ to the widest peak of LD within the window, which was used to define the boundaries of the region. If LD did not drop for at least 100 kb on either side of the LD peak, window size was increased by 1 Mb until the region could be defined. An LD- bound region was identified as shared if it was within the boundaries of another LD-bound region and prioritized as described below.

#### 2.5.3. Prioritization

**Figure 1:**
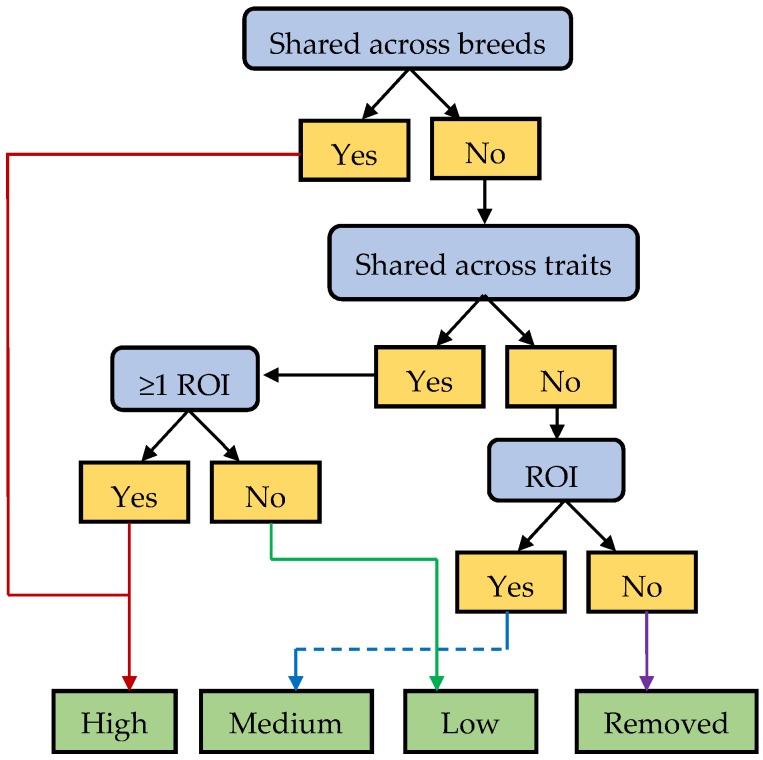
Flow chart of the prioritization of the regions identified on GWA.

Regions were prioritized if they were identified as shared between breeds on meta-analysis (MA-ROI), shared across traits within a single GWA cohort (for example, a region shared between insulin and adiponectin in the Morgan horses), or breed specific. The prioritized regions were categorized as high, medium or low priority (Figure 1) as follows:

- High priority: Region was identified as an MA-ROI or it was shared across traits with at least one region being considered an ROI.
- Medium priority: Region was identified as an ROI in at least one GWA cohort.
- Low priority: Region was shared across traits, but no regions met the criteria to be considered an ROI.
- If a region met the criteria for more than one category (for example a region identified as a MA-ROI and was also shared across traits but not an ROI) then the region was assigned the higher priority level.

#### 2.5.4. Identification of Positional Candidate Genes and Functional Enrichment Analysis

Positional candidate genes were identified using the Bioconductor/R software package biomaRt [30] with EquCab3 as the reference genome [31]. Positional candidate genes were defined as all protein coding genes, pseudogenes, and RNA genes within each GWA region as defined by the fixed-size region or the LD-bound regions as described above. Based on the comparison between the fixed-size and LD-bound regions, protein-coding genes within all prioritized LD-bound regions were assessed for functional enrichment analysis between traits using a multi-query approach available in the g: Profiler toolset [32].

## 3. Results

### 3.1. GWA Results

The union of GWA results across all twelve traits for the Welsh ponies identified 303 regions where at least one SNP exceeded the suggestive threshold. Of these regions, 64 were considered ROI (i.e., five SNPs exceeding the suggestive threshold and one or more SNPs exceeded the threshold for genome-wide significance) and included: seven ROI for baseline insulin, one ROI for INS-OST, three ROI for baseline glucose, two ROI for GLU-OST, two ROI for NEFA, one ROI for triglycerides, two ROI for adiponectin, three ROI for leptin, three ROI for ACTH, 14 ROI for NH, 16 ROI for GH, and 10 ROI for laminitis status (Tables S1 and S2).

GWA across all twelve traits for the Morgan horses identified 142 regions where at least one SNP exceeded the suggestive threshold. Of these regions, 37 ROI were identified and included one ROI for baseline insulin, one ROI for INS-OST, two ROI for baseline glucose, three ROI for GLU-OST, four ROI for NEFA, four ROI for adiponectin, three ROI for leptin, three ROI for ACTH, five ROI for NH, four ROI for GH, and seven ROI for laminitis status (Table S1).

### 3.2. Shared Regions Across Welsh Ponies and Morgan Horses and Across Traits

**Figure 2:**
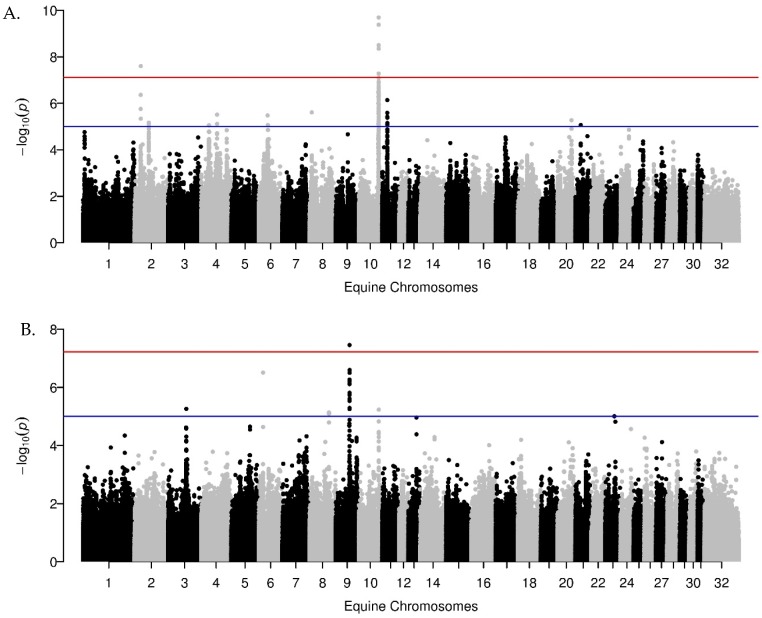
Manhattan plots of the genome-wide association results for insulin concentration post oral sugar test in (**A**) Morgan horses and (**B**) the section A and B Welsh ponies. The equine chromosomes (ECA) are plotted on the x-axis and the −log of the *p*-value is plotted on the y-axis. The blue line indicates the suggestive threshold (1.0 × 10^−05^) and the red line represents the genome-wide significant threshold (7.61 × 10^−08^ in the Morgan horses and 5.98 × 10^−08^ the Welsh ponies). In the section A, B, C and D Welsh ponies (not shown) and A and B Welsh ponies (B), the same region on ECA10 exceeds the suggestive threshold, which was also identified as an ROI in the Morgan horses (A). GWA meta-analysis identified this region as shared across both breeds.

Identification of the shared regions between the Morgan horses and the Welsh pony cohort from the boundaries of the fixed region obtained from the GWA results identified one shared region for laminitis status, one shared region for ACTH, and one shared region for insulin-OST (Figure 2). The boundaries defined by LD identified the above shared regions as well as an additional shared region for GH on ECA 22.

Meta-analysis across breeds identified the four shared regions above, as well as an additional 56 regions and five unique regions (regions not identified in either breed as significant on GWA), for a total of 65 shared regions of interest (MA-ROI). MA-ROI included two for insulin and four for glucose post oral sugar challenge, three for insulin, two for glucose, four for NEFA, seven for adiponectin, five for leptin, 15 for NH, eight for GH, and 12 for laminitis status. Unique regions were found for insulin (one MA-ROI) and glucose (one MA-ROI) post oral sugar test and NH (three MA-ROI). Across the MA-ROI, three regions were also shared across traits. No MA-ROI were identified for plasma triglyceride levels (Table 1).

Of the 56 regions identified on meta-analysis that were only significant in one breed-specific GWA, 30 (22 ROI) were called in at least one Welsh pony cohort and 26 (20 ROI) were called in the Morgan horses. Twenty-eight of the MA-ROI contained less than five SNPs of which 11 were single SNP regions. Comparison of the results using a fixed effects model identified 32 of the 65 MA-ROI (Table 1).

### 3.3. Prioritization of GWA Results and Identification of Positional Candidate Genes Based on Fixed-Size Regions in Welsh Ponies

For the Welsh pony cohorts, 189 of the 303 regions were eliminated from further prioritization. For the remaining 114 regions, 46 regions were considered high priority regions and contained 890 positional candidate genes, 34 regions were considered medium priority regions and contained 719 positional candidate genes, and 35 regions were considered low priority regions and contained 289 positional candidate genes. Accounting for the 19 shared regions resulted in 91 unique regions and 1511 positional candidate genes (Table S7).

### 3.4. Prioritization of GWA Results and Identification of Positional Candidate Genes Based on Fixed-Size Regions in Morgan Horses

For the Morgan horses, 88 of the 142 regions were eliminated from further prioritization (Table S8). This resulted in 54 regions being prioritized and 1104 positional candidate genes with 38 high priority regions containing 801 positional candidate genes, eight medium priority regions containing 139 positional candidate genes, and eight low priority regions containing 164 positional candidate genes. Accounting for the 10 shared regions resulted in 44 unique regions and 963 positional candidate genes (Tables S8 and S9).

### 3.5. Prioritization of LD-Bound GWA Regions, Identification of Positional Candidate Genes, and Network Analysis in Welsh Ponies

In the Welsh ponies, the LD-bound regions identified five additional regions shared across traits (ECA1 for adiponectin and INS-OST, ECA5 for insulin and leptin, ECA6 for leptin and GH, ECA9 for INS-OST and NEFA, and ECA18 for insulin and GH). However, the LD-bound regions did not identify six regions as shared across traits that were identified with the fixed-size boundaries (ECA4 for leptin and GH, ECA10 for NH and GH, ECA14 for leptin and laminitis status, ECA19 for ACTH and laminitis status, ECA 28 for insulin and INS-OST, and ECA28 for adiponectin and leptin). This resulted in 214 regions being removed and 89 regions being prioritized with 56 high priority regions containing 1567 positional candidate genes (Table 2). Further, 26 regions were given medium priority and contained 620 positional candidate genes and seven regions were given low priority and contained 30 positional candidate genes for a total of 2217 positional candidate genes. Accounting for the 18 shared regions resulted in 1853 positional candidate genes (Table S13).

Across nine EMS traits, functional enrichment analysis of the protein-coding genes within the prioritized regions identified enrichment (adjusted *p*-value < 0.05) for Gene Ontology (GO) terms associated with inflammation and fatty acid metabolism, including: interleukin-1 receptor binding (GH), lipid antigen binding (insulin), RAGE receptor binding (insulin and leptin), toll-like receptor binding (insulin and leptin), fatty-acyl-coA-synthase (INS-OST), cytokine activity (ACTH, NEFA, GH and NH), and regulation of NK-kappa signaling (GH, glucose, insulin, laminitis status, leptin, NEFA and NH). Enrichment for protein-coding genes associated with the GO terms thiosulfate sulfurtransferase activity was identified for NEFA concentrations (*p*-value = 4.9 × 10^−02^).

### 3.6. Prioritization of LD-Bound GWA Regions, Identification of Positional Candidate Genes, and Network Analysis in Morgan Horses

The LD-bound GWA regions for the Morgan horse identified three additional regions shared across traits (ECA 21 for triglycerides and adiponectin, ECA 6 for adiponectin and INS-OST, and ECA 19 for NH and laminitis status), but did not identify two regions as shared across traits that were identified with the fixed boundaries (ECA 20 for adiponectin and insulin and ECA 24 for insulin and NEFA) (Table S14). This resulted in 39 high priority regions containing 1142 positional candidate genes (Table 3). Further, eight regions were assigned medium priority and contained 155 positional candidate genes, and nine regions were assigned low priority and contained 176 positional candidate genes for a total of 1473 positional candidate genes. Accounting for the 12 shared regions resulted in 1167 positional candidate genes (Tables S14 and S15).

Functional enrichment analysis of the protein-coding genes within the prioritized regions identified enrichment (adjusted *p*-value < 0.05) for the Kyoto Encyclopedia of Genes and Genomes (KEGG) terms PI3K-AKT signaling pathway (ACTH, adiponectin, GH, glucose-OST, INS-OST, laminitis status, leptin, NEFA, and NH) and the Wnt-signaling pathway (ACTH, adiponectin, INS-OST, NH, and TG). Enrichment was also identified for the GO terms arylesterase activity for adiponectin (adjusted *p*-value = 1.3 × 10^−02^) and G-protein-coupled receptor activity for leptin (adjusted *p*-value = 1.2 × 10^−21^).

## 4. Discussion

In this study, we used high density SNP genotype data and GWA in two high risk breeds to identify hundreds of regions of the genome contributing to 12 EMS traits. Both fixed (500 kb) and linkage disequilibrium-based approaches were used to identify the boundaries of genomic regions identified on GWA and from there positional candidate genes within these regions. Within breed prioritization of the LD-bound regions was used to begin the process of organizing and managing this large list, and resulted in 56 high, 26 medium, and seven low priority regions, for a total of 1853 candidate genes in the Welsh ponies; and 39 high, eight medium, and nine low priority regions, for a total of 1167 candidate genes in the Morgan horses. Meta-analysis demonstrated that 65 of these regions were shared across breeds. These data support the hypothesis that EMS is a polygenic trait with both across breed and breed-specific genetic variants and predict that identifying even the major functional variants and risk loci will be a challenging task.

Comparison of the regions identified from the within breed GWA identified fewer shared regions across breeds than we anticipated. This could indicate that breed differences account for more of the risk alleles for EMS than previously thought, or that additional regions were shared but not identified in the breed-specific GWA, which can occur for several reasons. First, if the allele frequency of the variant is low in one breed, then it will not be detected on that GWA. Second, the effect of the variant on a trait can vary between breeds. The within-breed population sizes were powered to detect variants of moderate to high effect but would not find variants of low effect [33,34]. Third, variations in recombination of the ancestral chromosome can lead to differences in marker alleles between populations [35]. Depending on the markers represented on the genotyping array, the variant may be identified in one breed but not the other. Increasing the power of the study by performing across-breed GWA could identify more shared regions between breeds. However, combining data can lead to the inclusion of additional population substructure and unknown confounding variables into the model [36]. Therefore, we used meta-analysis to increase the number of individuals within the analysis and to improve the power to find unique associations, variants of low effect, and additional shared regions across breeds [37,38], and identified 65 shared regions of which five were unique (not identified in either breed specific GWA). 

Selective breeding can lead to population stratification within breeds [39], and not accounting for this population stratification can lead to spurious associations on GWA [40]. For this data, principal components analysis revealed population stratification in the Welsh pony cohort based on clustering of the registered sections (Figure A1). This was not unexpected as the Welsh pony sections are distinct subpopulations based on pedigree and conformation. Mixed linear models are a common way to account for population stratification and relatedness in GWA with the inclusion of a genetic relationship matrix (GRM) [18,41]. However, the Welsh ponies presented a unique challenge since, although the GRM would account for genetic similarities between Welsh pony sections, it would not account for all the phenotypic (conformation traits) variation between sections. On the other hand, including both the GRM and section as a covariate would lead to over-fitting of the model by accounting for relatedness both as a random effect (GRM) and fixed effect (section). Therefore, to account for population stratification within our Welsh pony cohort while maximizing sensitivity to identify genetic variations contributing to EMS both within and across sections, we chose to perform the GWA using the full data set and then subset the data to the section A, B, C and D ponies, and the section A and B ponies. Notably, there is likely a degree of population segregation in the section A and B which we are not fully controlling with a mixed model analysis, and ideally, we would have included the section A, B, C and D ponies as a separate GWA cohorts in order to account for all population substructure; however, our sample size made these analyses unfeasible due to the limited statistical power.

To reduce false positives, regions were prioritized based on sharing across breeds and traits. Regions shared across breeds (MA-ROI) were given high priority, as these regions were not breed specific and likely to be found in other high-risk breeds. Regions shared across traits with at least one ROI were also assigned high priority since many components and downstream effects of the endocrine system are highly interrelated; therefore, a variant affecting multiple traits would be expected to have a larger biological effect then a variant affecting a single trait. An ROI identified in one GWA cohort was assigned medium priority as these regions were likely breed or section (Welsh pony) specific and, based on the power of our study, variants of moderate to high effect. Finally, regions that were not an ROI but shared across traits were assigned low priority, because these regions were identified across multiple GWA and were less likely to be false positives and/or regions that contain variants of low effect.

Our prioritization removed 71% of the 303 GWA regions for the Welsh ponies and 58% of the 142 GWA regions in the Morgan horses. Of the removed regions, 49% were single SNP regions, 38% were regions with less than five SNPs, and 13% were regions with greater than or equals to five SNPs but no SNPs which exceeded the threshold for genome wide significance. Given: (i) the large percentage of single or low SNP regions that were removed, (ii) the high-density genotype data used in these analyses, and (iii) the use of the max gamma value for BSLMM (improving sensitivity at the cost of specificity), it is likely most of these regions were false positives. However, we utilized Bonferroni corrected p-values which tend to be more conservative corrections [42]; therefore, some of the removed regions may harbor genetic variants associated with EMS but represent variants with very low effect or poorly annotated regions of the genome (relative decreased number of SNPs in that region). Increasing the number of individuals, or represented Welsh pony sections, would improve the power of the study to determine which of these regions were true or false positives.

In order to identify candidate genes, we first used a fixed boundary of 500 kb 5′ of the SNP identified on GWA with the lowest base pair position and 3’ of the SNP with the highest base pair position. 500 kb was chosen based on the average distance for LD to breakdown in Thoroughbred horses [26,27,29]. Although LD decay varies between horse breeds [28], using the more conservative Thoroughbred estimate gave a higher likelihood that we would capture all variants within LD (r^2^ > 0.3) of the marker SNPs in our cohorts. From the fixed boundaries, 1511 and 963 positional candidate genes were identified in the Welsh ponies and Morgan horses, respectively. 

Estimates of LD decay are based on the average r^2^ across chromosomal segments and do not represent specific regions of the genome [26,28]. Newer variants or variants within regions under selection will have longer LD blocks whereas older/ancestral variants will have shorter LD blocks due to longer periods of recombination. Therefore, using a fixed region has the potential to exclude causal variants or to include candidate genes that are not in LD with the marker SNPs. To more precisely call positional candidate genes for GWA regions, we calculated LD using the squared correlation coefficients between SNPs. SNPs within LD were defined as an r^2^ > 0.3 [28]. Boundaries were identified based on gaps of LD, i.e. where all SNPs dropped below 0.3 for a span of 100 kb 5′ (defined the start of the LD block) and 3’ (defined the end of LD block) to the widest peak of LD.

Across all Welsh pony cohorts, 70% of the boundaries identified by LD were smaller than those identified by the fixed-size region, with an average difference of 645.4 kb (range of 11.4 kb to 1.7 Mb); whereas, in the Morgan horses, 57% of the LD boundaries were smaller than that of the fixed regions, with an average difference of 566.6 kb (range of 51.5 kb to 2.2 Mb). The large percentage of fixed-size boundaries, likely overestimating the region size, is not surprising given that the fixed-size regions were based on data from Thoroughbreds which have one of the highest inbreeding coefficients and LD amongst horse breeds [24,28]. Ponies and Morgan horses were identified to have LD similar to Quarter Horses [28], a breed with a high level of genetic diversity. For the remaining regions, the LD boundaries were an average of 1.9 Mb longer (range of 22.8 kb to 9.3 Mb) in the Welsh ponies and 1.4 Mb longer (range of 12.6 kb to 8.2 Mb) in the Morgan horses then defined by the fixed-size region and likely represent regions under selection. 

Results of the functional enrichment analysis of the prioritized regions based on LD provided support for our approach. In the Welsh ponies, nine traits were enriched for pathways associated with inflammation or fatty acid metabolism. This is not surprising given that chronic, low-grade inflammation and dyslipidemia are common components of EMS. The latter results are also consistent with human GWA studies of MetS which have consistently identified a large number of variants related to lipid metabolism [43,44,45]. In addition, the GO term thiosulfate sulfurtransferase (*Tst, Rhodanese*) activity was enriched in positional genes identified for plasma NEFA concentrations. *Tst* encodes a mitochondrial enzyme which is involved in mitochondrial energetics and removal of reactivate-oxygen species and was identified as a candidate obesity-resistant gene in the polygenic Lean mouse model [46]. In this study, the authors evaluated *Tst* mRNA concentrations in multiple cross-ethnic human populations and found that they were higher in lean individuals compared with obese individuals or individuals with type 2 diabetes and negatively correlated with body mass index [46]. 

In the Morgan horses, the KEEG terms for PI3K/AKT and Wnt signaling pathways were functionally enriched across several traits. Both of these pathways have significant roles in metabolism, with the PI3K/AKT signaling pathway being essential for glucose homeostasis and lipid metabolism [47,48], and the Wnt signaling pathway regulating body mass, glucose metabolism, de novo lipogenesis, and low-density lipoprotein clearance [49]. Further, the GO term arylesterase (*ARE*) activity was enriched for plasma adiponectin concentrations. *ARE* activity has been linked to the paraoxonase-1 gene which has roles in lipoprotein oxidative damage prevention and being protective against metabolic syndrome [50,51]. Lastly, serum leptin concentrations were enriched for the GO term G-protein-coupled receptor activity, which have been found to be directly involved in pancreatic β-cell destruction and insulin resistance and remain potential targets for drug therapy for metabolic syndrome and type II diabetes [52]. 

## 5. Conclusions

In conclusion, the results of these data provide strong evidence that EMS is a complex, polygenic syndrome with dozens of risk alleles contributing to the phenotype. Prioritization of the hundreds of regions identified on the GWA of 12 individual traits led to the identification of thousands of positional candidate genes which contained protein-coding genes which were functionally enriched for pathways associated with glucose metabolism, lipid metabolism and inflammation. However, further work is required to narrow down the candidate gene pool. Nonetheless, these data were an important first step in the identification of the genetic risk alleles associated with EMS. In addition, this data concludes that a single variant genetic test will not provide enough information to accurately predict an individual’s risk for EMS, and a future DNA test will require a panel of genetic variants including both shared and breed specific variants.

## Figures and Tables

**Table 1:** Meta-analysis across breeds and across EMS metabolic traits.

Trait	Chr	Min_SNP	Max_SNP	Sugg_SNPs	Sign_SNPs	FE
**Insulin**	5	44104129	45081679	39	4	-
	15	5887873	6225014	21	2	-
	24	28804043	29076914	3	2	-
**INS-OST**	10	71620835	72425049	19	4	X
	28	38307699	38344594	2	1	-
**Glucose**	4	18053357	18550035	20	1	-
	8	9289661	9312611	2	1	-
**GLU-OST**	3	55982921	56558742	57	39	X
	4	27802674	28514796	18	4	X
	15	79697363	79717603	3	3	-
	28	34861664	34868420	2	2	-
**NEFA**	1	183532379	184178932	21	15	X
	17	13355958	14014858	23	1	X
	24	20975408	NA	1	1	-
	30	20148173	20205201	10	3	X
**Adiponectin**	2	16725632	17531903	25	19	X
	4	37105938	37523046	6	2	X
	6	31582345	31708194	17	1	X
	6	67097628	68036518	16	1	-
	18	41399862	41533081	9	1	-
	18	60138400	60241267	10	2	-
	20	3447045	3609674	10	4	X
**Leptin**	7	65731012	65804974	6	3	X
	10	871456	NA	1	1	-
	19	48839140	49627683	44	22	X
	24	28551544	28744981	17	6	-
**ACTH**	1	69730886	70257187	4	1	-
	1	82755708	82879246	10	1	-
	3	41684754	NA	1	1	-
	3	101236287	101618645	42	24	X
	5	28822515	29342972	12	3	X
	10	78846710	NA	1	1	-
**NH**	1	88009187	NA	1	1	-
	3	58464229	NA	1	1	-
	4	51903203	53474757	64	40	-
	6	63614756	63814984	20	10	-
	9	22745020	NA	1	1	-
	9	33549797	34165892	31	1	-
	11	18987272	19176693	10	8	-
	14	63778931	63876998	7	2	X
	19	1134701	1139669	2	2	-
	19	32230245	33643392	55	2	-
	20	39797561	40162785	7	4	X
	20	59659997	60403627	11	4	X
	21	20193411	21256032	18	11	X
	24	33852631	34812035	36	23	X
**GH**	1	121484057	121775873	47	19	-
	**1**	131512239	131621826	3	3	X
	4	84181768	85275183	29	11	X
	11	18987272	19176693	10	9	X
	17	32120145	32544617	23	4	X
	19	28934939	NA	1	1	-
	20	63560971	63691145	10	6	-
	22	40135963	40167502	4	4	X
**LAM**	1	49077969	NA	1	1	-
	2	36104151	36108219	6	6	-
	4	17765473	18991639	11	3	-
	12	29378128	30296509	19	11	X
	14	88430222	89591967	20	5	-
	18	31679672	33134556	51	26	X
	19	28057756	28417335	5	2	-
	19	57605404	58429206	36	20	X
	22	3565315	4307679	62	38	X
	23	12226548	12763020	35	24	X
	28	9446507	9643240	13	5	X

To be considered an MA-ROI, at least one SNP had to exceed the threshold for genome-wide significance (1.6 × 10^−07^) on meta-analysis. Provided is the base pair position of the lowest (Min_SNP) and highest (Max_SNP) SNP, as well as the number of SNPs per region which exceeded the suggestive (Sugg_SNPs) and genome-wide significance (Sign_SNPs) threshold. Regions shared across two traits in the meta-analysis are represented by the corresponding highlighted chromosomes (Chr). Regions which were statistically significant using a standard fixed effects models (FE) are indicated by an X.

**Table 2:** LD-bound high priority GWA regions for the Welsh pony cohorts.

Trait	Chr	Min	Max	Protein Coding	RNA Genes	Total Genes
**Insulin**	5	35409104	44806458	267	38	306
	8	69350844	75906595	32	23	55
	15	5748377	6612684	0	1	1
	18	78720858	79634082	2	4	6
	24	28451012	29887250	2	4	6
**INS-OST**	1	176773704	176873704	0	1	1
	8	73173455	73699198	1	1	2
	10	71967783	72438937	3	0	3
	28	39322188	39488807	8	1	9
**Glucose**	15	83728178	83828178	2	0	2
**GLU-OST**	28	34271949	35138699	9	0	9
**Adiponectin**	1	171861236	178270042	25	24	49
	18	60060215	61349045	7	6	13
**Leptin**	5	39751797	50431769	207	32	239
	6	488137	4012580	15	10	25
	7	65678376	68117086	1	2	3
	10	692055	1068890	0	5	5
	21	22940681	23516697	1	0	1
**NEFA**	19	1005718	1105718	2	0	2
	28	32909542	35703535	65	11	76
**ACTH**	1	42944403	45232767	5	4	9
	1	69558737	70960589	7	16	23
	10	55060512	56255134	1	1	2
	10	78795710	80306613	20	5	25
	20	60381850	60481850	0	0	0
**NH**	4	67130904	69873296	8	8	16
	4	77298241	81186565	24	15	40
	4	83144842	83244842	1	0	1
	7	93176991	93628686	0	1	1
	9	32632235	37587269	10	8	18
	11	18342117	19876247	55	4	60
	14	63702522	63847210	0	2	2
	20	40244007	41210876	3	11	14
	20	60723014	61735694	0	2	2
	21	5280993	6396786	2	6	8
	21	19515280	25046226	22	27	49
	24	31843480	36758215	47	10	57
**GH**	1	132184772	133716124	9	7	16
	4	68425678	69636837	5	1	6
	4	70026254	81648125	49	45	95
	4	82570011	86366835	49	25	75
	7	93191676	93628672	0	1	1
	11	15414337	16451463	24	1	25
	11	18613895	19317536	26	0	26
	18	79527484	81467661	13	12	25
	19	31204596	31799125	0	0	0
	20	29486630	30976763	54	8	62
	20	59464566	61015217	1	2	3
	20	64722427	65336095	1	3	4
	21	20611963	22057711	3	4	7
	22	41032889	41066045	0	0	0
	25	19435041	19535041	4	0	4
**LAM**	1	49391032	49491032	0	1	1
	2	35880861	36665473	8	6	14
	19	57082025	62825378	42	16	59
	28	9990892	10844823	4	0	4
**Total**				1146	415	1567

Final region boundaries were based on LD and are indicated by the lowest base pair position (Min) and the highest base pair position (Max). The total number of genes includes all protein-coding genes, pseudogenes, and RNA genes identified for region based on EquCab3. Shared regions across prioritized traits are indicated by highlighted chromosomes.

**Table 3:** LD-bound high priority GWA regions for the Morgan horses.

Trait	Chr	Min	Max	Protein Coding	RNA Genes	Total Genes
**Insulin**	24	21134897	21184897	1	0	1
**INS-OST**	4	28373202	28423202	0	0	0
	6	32751552	34029749	12	10	22
	10	71666607	73534053	6	5	12
**Glucose**	4	17239374	19043831	6	5	11
	8	11193683	12404572	8	9	17
**GLU-OST**	3	55746338	58085997	15	6	21
	4	26695616	29116058	5	4	9
**NEFA**	1	184859013	187238015	24	17	41
	17	12653835	14464765	4	2	6
	24	20287835	20973401	15	1	16
	30	20915473	21380977	0	0	0
**Adiponectin**	2	16362904	18105119	21	21	42
	4	34723398	39321960	36	10	47
	6	32486287	32841880	3	4	7
	6	64297403	71493047	168	22	191
	18	41448414	41498414	0	1	1
	20	3649052	4325872	8	3	11
**Leptin**	4	51590680	52810437	4	5	9
	19	51286493	53959028	7	14	21
	24	25564765	29384679	7	14	21
	1	82700933	84269783	18	5	24
	3	42674448	44422013	2	7	10
	3	102944842	103801021	2	4	6
	5	25378878	27689002	12	16	28
**NH**	1	82097718	83618523	14	6	20
	4	52024470	54237747	8	12	20
	6	60410647	70570773	144	26	172
	19	661978	1345372	4	1	6
	19	32962795	37391949	53	19	73
**GH**	1	120644115	124691346	36	20	56
	17	31806060	33720086	3	3	7
**LAM**	4	17301415	19812653	8	7	16
	12	32885278	34800986	29	16	45
	14	87916190	91602875	32	26	58
	18	30095266	35177011	23	13	36
	19	30133826	30183826	2	0	2
	22	2843476	5225020	13	10	23
	23	7656404	12984095	11	23	34
**Total**				764	367	1142

Final region boundaries were based on LD and are indicated by the lowest base pair position (Min) and the highest base pair position (Max). The total number of genes includes all protein-coding genes, pseudogenes, and RNA genes identified for region based on EquCab3. Shared regions across prioritized traits are indicated by highlighted chromosomes.

**Figure A2:**
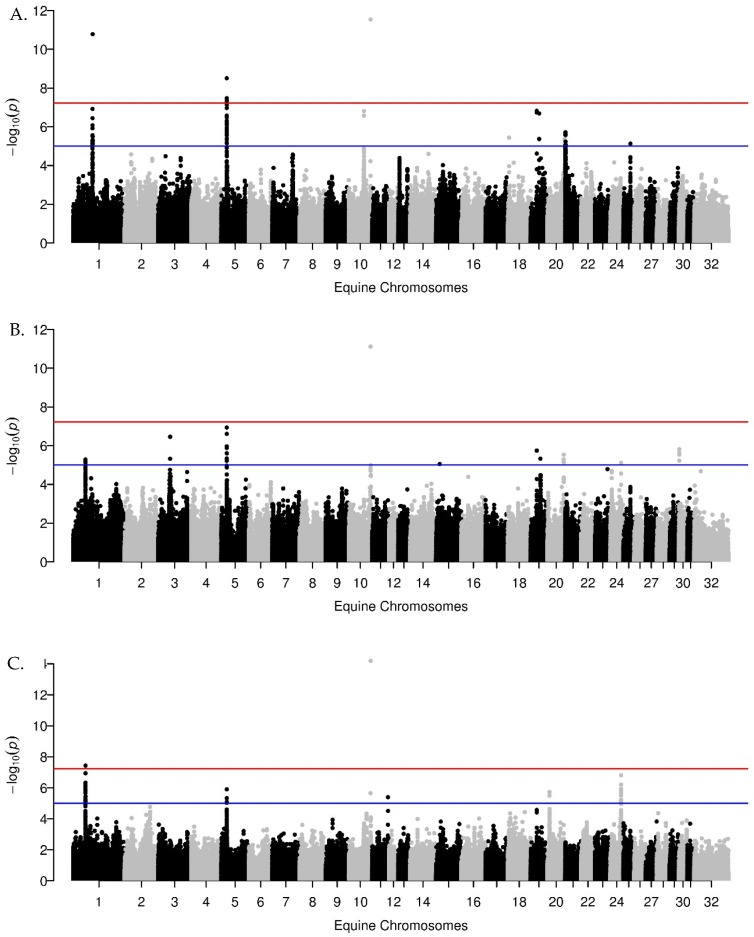
Manhattan plots of the genome-wide association results for ACTH in (**A**) full Welsh pony cohort, (**B**) the section A, B, C and D Welsh ponies, and (**C**) the section A and B Welsh ponies. The equine chromosomes (ECA) are plotted on the x-axis and the −log10 of the *p*-value is plotted on the y-axis. The blue line indicates the suggestive threshold (1.0 × 10^−05^) and the red line represents the genome-wide significant threshold (5.9 × 10^−08^). In all three GWA, the same region on ECA5 exceeds the suggestive threshold but is only identified as an ROI in the full cohort (A).

## Supplementary Materials

The following are available online at https://www.mdpi.com/2073-4425/10/11/893/s1, Figure S1: Linkage disequilibrium (LD) for neck-to-height-ratio (NH) on equine chromosome 4 (ECA4) in the Morgan horses, Table S1: Summary table for GWA regions for each of the 12 EMS traits in Welsh ponies and Morgan horses, Table S2: Summary table of the shared regions across two or three cohorts for each of the 12 EMS traits from the Welsh pony GWA, Table S3: Shared regions from the Welsh pony GWA, Table S4: Prioritization of the GWA results of the full Welsh pony cohort based on fixed-size regions, Table S5: Prioritization of the GWA results of the section A, B, C and D Welsh ponies based on fixed-size regions, Table S6: Prioritization of the GWA results of the section A and B Welsh ponies based on fixed-size regions, Table S7: Final prioritization of the GWA results based on a fixed-size region for the Welsh ponies, Table S8: Prioritization of the GWA results of the Morgan horses based on fixed-size regions, Table S9: Final prioritization of the GWA results based on a fixed-size region for the Morgan horses, Table S10: Prioritization of the GWA results of the full Welsh pony cohort based on LD-bound regions, Table S11: Prioritization of the GWA results of the section A, B, C and D Welsh ponies based on LD-bound regions, Table S12: Prioritization of the GWA results of the section A and B Welsh ponies based on LD-bound regions, Table S13: Final prioritization of the GWA results based on an LD-bound region for the Welsh ponies, Table S14: Prioritization of the GWA results of the Morgan horses based on LD-bound regions, Table S15: Final prioritization of the GWA results based on an LD-bound region for the Morgan horses.

## Appendix A

### Appendix A.1. Welsh Pony Population Structure

The Welsh Pony and Cob Society (http://wpcs.uk.com) registers Welsh ponies into six sections based on pedigree, withers height and conformation as follows: section A (sire and dam must both be section A, and the pony can be up to 50 inches for withers height), section B (either sire and dam are both section B or one parent can be a section A, and the pony can be up to 58 inches for withers height), section C (at least one parent must be C or D and the pony can be up to 54 inches for withers height), section D (at least one parent must be C or D and the pony must be over 54 inches for withers height), section H (either the sire or dam is a registered Welsh pony, and there are no height restrictions) and section P (either sire or dam is at least 50% Welsh pony with no height restrictions).

### Appendix A.2. GWA Results

For the Welsh ponies, GWA across all 12 traits resulted in 130 regions where at least one SNP exceeded the suggestive threshold, of which 33 were identified as ROI (entire Welsh pony cohort; *n* = 264), 139 regions where at least one SNP exceeded the suggestive threshold, of which 23 were identified as ROI (section A, B, C, and D Welsh ponies; *n* = 238), and 82 regions where at least one SNP exceeded the suggestive threshold, of which 13 were identified as ROI (section A and B Welsh ponies; *n* = 220) (Table S1).

Across all 12 traits, 38 regions were shared with two of the Welsh pony GWA cohorts and 5 regions were shared with all three of them. Fifteen of the 43 shared regions contained at least one GWA where the region met the criteria to be considered an ROI (Tables S2 and S3). The 43 shared regions represented 18.9%, 26.6%, and 30.5% of the total regions identified in the full cohort, the section A, B, C and D Welsh ponies, and the section A and B Welsh ponies, respectively. Eight regions had an ROI identified in the full cohort (24.2% of the total ROI for this cohort), six regions had an ROI identified in the section A, B, C and D ponies (26.1% of the total ROI for this cohort), and six regions had an ROI identified in the section A and B ponies (46.2% of the total ROI identified in this cohort). For example, analysis of ACTH identified five shared regions. The region on equine chromosome (ECA) 5 was shared across all three cohorts but was only identified as an ROI in the GWA of the full cohort (Figure A2 and Table S3).

### Appendix A.3. Prioritization of GWA Results and Identification of Positional Candidate Genes Based on Fixed-Size Regions in Welsh Ponies

For the full Welsh pony cohort, 78 of the 130 regions were eliminated from further prioritization, 35 were categorized as high priority, 12 were categorized as medium priority and five were categorized as low priority (Table S4). For the section A, B, C and D Welsh ponies, 94 of the 139 regions were eliminated from further prioritization, 19 were categorized as high priority, 19 were categorized as medium priority and eight were categorized as low priority (Table S5). For the section A and B Welsh ponies, 57 of the 82 regions identified on GWA were eliminated from further prioritization, nine were categorized as high priority, 10 were categorized as medium priority and six were categorized as low priority (Table S6).

### Appendix A.4. Prioritization of LD-Bound GWA Regions, Identification of Positional Candidate Genes, and Network Analysis in Welsh Ponies

The LD boundaries for the 130 regions identified on GWA for the full Welsh pony cohort, the 139 regions identified on GWA for the Section A, B, C and D Welsh ponies, and the 82 regions identified on GWA for the Section A and B Welsh ponies are presented in Tables S10–S12, respectively. Our prioritization removed 61% of the 130 regions for the full Welsh pony cohort, 66% of the 139 regions in the section A, B, C and D Welsh ponies, 63% of the 82 regions in the section A and B Welsh ponies.

### Appendix A.5. Shared Regions Across Welsh Ponies and Morgan Horses

Identification of the shared regions between the Morgan horses and at least one Welsh pony cohort from the boundaries of the fixed region obtained from the GWA results identified one shared region for laminitis status (all ponies), one shared region for ACTH (Morgan horses with section A, B, C, and D ponies), and one shared region for INS-OST (for Morgan horses with both the section A, B, C, and D and section A and B ponies; Figure 2). The boundaries defined by the LD-region identified the above shared regions as well as an additional shared region for GH on ECA 22 between the Morgan horses and the full Welsh pony cohort.

## Appendix B

### Appendix B.1. Description of the Mixed Liner Model Used for Genome-Wide Association Analyses

Within breed GWA were performed from the imputed SNP genotype data using a custom code for an improved mixed linear regression analysis [9]. This algorithm utilizes a three-step process, which combines a Bayesian Sparse Linear Mixed Model (BSLMM) [18] available in the software program Genome-Wide Efficient Mixed Model Association (GEMMA) [19] and a linear mixed model implemented in FaST-LMM [20]. In step one, the genome is divided by chromosome and SNPs are placed into 500 kb bins. Based on results from BSLMM, the SNP with the highest model frequency and the two adjacent SNPs were chosen to represent the corresponding bin. In step two, a likelihood ratio test was performed to determine if inclusion of the top ranked bin as a random effect will improve the null model (model with sex and age as fixed effects and farm as a random effect). If the model was improved, the alternative model became the null model and the next highest-ranked bin was tested. If the model was not improved, the bin was discarded, and the next highest-ranked bin was tested against the null hypothesis. After all bins were evaluated, SNPs which improved the model were utilized to create the select SNP genetic relationship matrix (GRM). In step three, all imputed SNPs were tested for an effect on the trait using FastLMM with the inclusion of the select SNP GRM in place of the standard all-SNP GRM. For the GWA, the tested SNP, and SNPs within 1Mb of the tested SNP, were removed from the select SNP GRM to avoid proximal contamination.

The number of iterations for the Markov chain Monte Carlo (MCMC) implemented in BSLMM has not been previously assessed [9,17], and our initial results with the default of 550 thousand (k) iterations with 10 k burn-in iterations provided inconsistent results across seeds. Therefore, we took appropriate steps to determine the number of iterations for the MCMC to converge and provide consistent results across seeds. First, to assess the concordance of SNPs identified by BSLMM, we performed this step using 10 million (M) iterations with 100 K burn-in iterations, which was repeated across ten different seeds. SNPs with a beta value greater than zero (i.e. the posterior mean for SNPs which were estimated to have a large effect on the outcome variable) were extracted from the dataset for each seed. For this subset of SNPs, the intersect between seeds was determined, and correlations between gamma values (proportion of iterations that the SNP was estimated to have a large effect) were calculated. For 10 M iterations, the total number of SNPs with a beta value greater than zero ranged from 486,937 to 497,207 SNPs. Approximately 50% of the SNPs were shared between two seeds, ~13% were shared between four seeds, and ~3% were shared across all ten seeds. Pearson’s correlation coefficient between gamma values were minimal at <0.01. Thus, this process was repeated using 20 M iterations (200 K burn-in iterations) and then increasing in 10 M and 100 k increments up to 100 M iterations (1 M burn-in iterations). Although SNP concordance improved as the number of iterations increased, the gain plateaued after 50 M iterations where all SNPs had a beta value greater than zero. In addition, the Pearson’s correlation coefficient for gamma values was still poor at 0.20 at 100 M iteration. Computational time was extensive at 30 and 60 days to complete the 50 M and 100 M iterations, respectively, utilizing six processors per node and running seeds in parallel [53].

Previous studies have averaged the values of MCMC estimates across repeated chains [54]. For this analysis the goal was to maximize sensitivity; therefore, using data from the 10 M iterations, the max gamma value across all ten seeds was chosen to represent each SNP in which beta was greater than zero. These values were then used to choose the most influential SNP per 500 kb bins (step 1). To assess the repeatability of these results, this process was repeated using 10 different seeds at 10M iterations and 20 M iterations. Although differences were present, most regions were shared across all three results (Table A1). Thus, to maximize computational efficiency and sensitivity, we used the max gamma value across 10 seeds obtained from 10 M iterations (100 K burn-in) and prioritized regions of interest (see Section 2.5. Prioritization of GWA Regions and Identification of Positional Candidate Genes).

### Appendix B.2. Fixed and Random Effects

Age and sex were included in our model as fixed effects based on epidemiological studies which identified both as risk factors for EMS [55,56]. Season [57,58], diet [3,59], exercise [59,60], and endocrine-disrupting chemicals [61] have also been identified as environmental risk factors for EMS, but a large percentage of environmental variation has yet to be explained [9]. Further, several studies have produced conflicting findings as to the effect of season on EMS biochemical measurements [62,63], as well as the long-term effect of high non-structural carbohydrate diets on insulin sensitivity [64,65]. Therefore, we included farm as a random variable to account for both known and unknown environmental risk factors, as well as non-independent sampling of our data (each farm was required to have one control and one horse with EMS to be included in the study).

**Table A1:** Repeatability across results for the Bayesian sparse linear mixed model (BSLMM).

	10 M Iterations Seeds 1–10		10 M Iterations Seeds 11–20		20 M Iterations Seeds 1–10		30 M Iterations Seeds 1–10	
CHR	Sugg	Sign	Sugg	Sign	Sugg	Sign	Sugg	Sign
**1**	2	0	NA	NA	NA	NA	NA	NA
**1**	1	0	NA	NA	1	0	4	0
**2**	38	27	5	1	32	2	5	1
**3**	NA	NA	NA	NA	1	NA		
**4**	54	4	25	2	3	0	5	2
**6**	68	4	8	1	11	2	11	2
**7**	14	0	10	0	5	0	8	0
**8**	6	0	NA	NA	NA	NA	NA	NA
**9**	NA	NA	2	0	NA	NA	NA	NA
**14**	NA	NA	NA	NA	1	0	NA	NA
**15**	6	1	5	1	5	2	12	1
**16**	NA	NA	NA	NA	2	0	NA	NA
**18**	NA	NA	1	0	NA	NA	NA	NA
**19**	NA	NA	NA	NA	NA	NA	3	0
**20**	NA	NA	NA	NA	NA	NA	1	0
**22**	NA	NA	NA	NA	4	0	NA	NA
**23**	NA	NA	11	0	NA	NA	NA	NA
**24**	NA	NA	4	1	16	0	NA	NA

Repeatability across results for the Bayesian sparse linear mixed model (BSLMM) using the max gamma values from 10 million (M) iterations with seeds 1–10, 10 M iterations with seeds 11–20, 20 M iterations with seeds 1–10, and 30 M iterations with seeds 1–10 for adiponectin concentrations in the Morgan horses. Regions which are highlighted in yellow indicate those which would have been identified as a region of interest (contained a minimum of five SNPs exceeding the suggestive threshold, with at least one SNP exceeding the threshold for genome wide significance). *Abbreviations*: Sugg (total number of SNPs which exceeded the suggested threshold for genome-wide significance), Sign (total number of SNPs which exceed the threshold for genome-wide significance).
