# Supplementary material for: Genome-Wide Association Analyses of Equine Metabolic Syndrome Phenotypes in Welsh Ponies and Morgan Horses
Source: Genes (Basel). 2019 Nov 6;10(11):893. doi: 10.3390/genes10110893 (PMC6895807; doi:10.3390/genes10110893)
Supplement: Supplementary file 1 [file genes-10-00893-s001.zip › Norton_EMS_GWA_Supplementary_Tables.docx]

| **Table S1:** Summary table for GWA regions for each of the 12 EMS traits in Welsh ponies and Morgan horses | | | | | | | | |
| --- | --- | --- | --- | --- | --- | --- | --- | --- |
|  | **All Welsh ponies** | | **Section ABCD** | | **Section AB** | | **Morgan Horses** | |
| **Trait** | **Regions** | **ROI** | **Regions** | **ROI** | **Regions** | **ROI** | **Regions** | **ROI** |
| Insulin | 15 | 5 | 10 | 2 | 10 | 2 | 12 | 1 |
| Insulin-OST | 7 | 0 | 7 | 0 | 6 | 1 | 10 | 1 |
| Glucose | 8 | 0 | 7 | 0 | 8 | 3 | 6 | 2 |
| Glucose-OST | 5 | 1 | 4 | 0 | 3 | 1 | 6 | 3 |
| NEFA | 10 | 0 | 10 | 2 | 11 | 1 | 13 | 4 |
| TG | 3 | 0 | 5 | 0 | 6 | 1 | 4 | 0 |
| Adiponectin | 2 | 1 | 6 | 1 | 4 | 0 | 17 | 4 |
| Leptin | 7 | 2 | 16 | 1 | 3 | 0 | 8 | 3 |
| ACTH | 10 | 2 | 10 | 0 | 6 | 1 | 18 | 3 |
| NH | 22 | 9 | 22 | 5 | 6 | 1 | 16 | 5 |
| GH | 23 | 8 | 30 | 9 | 9 | 0 | 14 | 4 |
| LAM | 18 | 5 | 12 | 3 | 10 | 2 | 18 | 7 |
| **Total** | **130** | **33** | **139** | **23** | **82** | **13** | **142** | **37** |

The column listed as at least one ROI (region of interest) indicates that total number of shared regions where at least one region met the criteria to be considered an ROI (minimum of five SNPs with at least one SNP exceeding the threshold for genome wide significance). Abbreviations: OST: oral sugar test, NH: neck-to-height ratio, GH: girth-to-height ratio, LAM: laminitis status.

| **Table S2:** Summary table of the shared regions across two or three cohorts for each of the 12 EMS traits from the Welsh pony GWA | | | | | |
| --- | --- | --- | --- | --- | --- |
| **Trait** | **All WP &**  **Section ABCD** | **All WP &**  **Section AB** | **Section ABCD &**  **Section AB** | **All Three** | **At least one**  **ROI** |
| Insulin | 1 | 1 | 1 | 0 | 3 |
| Insulin-OST | 0 | 1 | 3 | 0 | 0 |
| Glucose | 1 | 1 | 0 | 0 | 1 |
| Glucose-OST | 1 | 0 | 2 | 0 | 1 |
| NEFA | 1 | 0 | 5 | 1 | 2 |
| TG | 1 | 0 | 1 | 0 | 0 |
| Adiponectin | 0 | 0 | 1 | 0 | 0 |
| Leptin | 1 | 0 | 0 | 1 | 1 |
| ACTH | 1 | 0 | 2 | 2 | 2 |
| NH | 4 | 0 | 0 | 1 | 2 |
| GH | 5 | 0 | 0 | 0 | 2 |
| LAM | 2 | 2 | 0 | 0 | 1 |
| **Total** | **18** | **5** | **15** | **5** | **15** |

Shared regions across two or three cohorts for each of the 12 EMS traits from the Welsh pony (WP) genome-wide association analyses (GWA) including the full cohort (n=264), individuals identified by pedigree as section A, B, C or D (n=238), individuals identified by pedigree as section A or B (n=220). The column listed as at least one ROI (region of interest) indicates that total number of shared regions where at least one region met the criteria to be considered an ROI (minimum of five SNPs exceeding the suggestive threshold with at least one SNP exceeding the threshold for genome wide significance).

| **Table S3:** Shared regions from the Welsh pony genome-wide association analyses | | | | | | | | |
| --- | --- | --- | --- | --- | --- | --- | --- | --- |
|  |  |  | **All WP** | | **Section ABCD WP** | | **Section AB WP** | |
| **Trait** | **Chr** | **Total**  **GWAS** | **Min_SNP** | **Max_SNP** | **Min_SNP** | **Max_SNP** | **Min_SNP** | **Max_SNP** |
| **Insulin** | 6 | 2 | **81074650** | **81566120** | **81421330** | **82660343** | - | - |
|  | 9 | 2 | - | - | **58976739** | **59099678** | **58477773** | **60003081** |
|  | 15 | 2 | **5887873** | **6278651** | - | - | 5899834 | NA |
| **Insulin-OST** | 3 | 2 | - | - | 65320573 | NA | 65980441 | NA |
|  | 6 | 2 | - | - | 15393073 | 15402993 | 15393073 | NA |
|  | 8 | 2 | 69942950 | 69982846 | - | - | 69942980 | 69982846 |
|  | 10 | 2 | - | - | 72158447 | 72240841 | 72238960 | NA |
| **Glucose** | 8 | 2 | 81424426 | 81518794 | 81284977 | 81428684 | - | - |
|  | 29 | 2 | 21472582 | 21475253 | - | - | **21440455** | **22135257** |
| **Glucose-OST** | 5 | 2 | - | - | 66212381 | 66719700 | 66618266 | 66719700 |
|  | 23 | 2 | 10907371 | 10951165 | 10942382 | 10951165 | - | - |
|  | 28 | 2 | - | - | 33915296 | NA | **33387547** | **33915296** |
| **NEFA** | 6 | 2 | 76161874 | NA | 76161874 | NA | - | - |
|  | 7 | 2 | - | - | 7268673 | 7382898 | 7268673 | NA |
|  | 7 | 2 | - | - | 8181330 | 8243021 | 7268673 | NA |
|  | 9 | 2 | - | - | **47030376** | **48595497** | **47219472** | **48722431** |
|  | 14 | 2 | - | - | 33871722 | NA | 33829080 | 33974280 |
|  | 22 | 3 | 18575108 | NA | 18575108 | NA | 18575108 | 18594384 |
|  | 28 | 2 | - | - | **33731242** | **34441427** | 33819949 | 33831231 |
| **TG** | 12 | 2 | 32054230 | 32083040 | 32072315 | 32083040 | - | - |
|  | 20 | 2 | - | - | 55609506 | 55705820 | 55239314 | NA |
| **Adiponectin** | 22 | 2 | - | - | 36975989 | 37058774 | 37058774 | NA |
| **Leptin** | 7 | 2 | **65773875** | **65782930** | 65773875 | 65782930 | - | - |
|  | 10 | 3 | 865540 | 883471 | 856640 | 883471 | 871456 | NA |
| **ACTH** | 1 | 2 | - | - | 44050526 | 44285580 | **43943376** | **44773532** |
|  | 5 | 3 | **19628265** | **20107907** | 19859591 | 20010745 | 19859591 | 20010745 |
|  | 10 | 3 | 78846710 | NA | 78845710 | NA | 78703637 | 78846710 |
|  | 19 | 2 | 21867680 | 21871015 | 21867680 | 21871015 | - | - |
|  | 24 | 2 | - | - | 39069140 | NA | 38145005 | 39231598 |
| **NH** | 4 | 3 | 67875816 | 68337160 | **68879163** | **69478180** | **67379332** | **69246252** |
|  | 4 | 2 | 77152103 | NA | 76199121 | 77653150 | - | - |
|  | 4 | 2 | 78075875 | 78460889 | 76199121 | 77653150 | - | - |
|  | 8 | 2 | 61139637 | 61236848 | 61139637 | 61177365 | - | - |
|  | 21 | 2 | **20193411** | **21497651** | 20193411 | 21059497 | - | - |
| **GH** | 1 | 2 | 119770589 | NA | 119519666 | 119549672 | - | - |
|  | 4 | 2 | 68337160 | NA | 69000484 | 69423480 | - | - |
|  | 4 | 2 | **84181768** | **85381459** | **83940435** | **85259515** | - | - |
|  | 20 | 2 | **29233068** | **29537740** | 29233068 | 29252036 | - | - |
|  | 21 | 2 | 21387986 | 21398724 | 20919577 | 20922494 | - | - |
| **LAM** | 1 | 2 | 49077969 | NA | 49077969 | NA | - | - |
|  | 2 | 2 | **35906741** | **36414648** | 36104151 | 36414648 | - | - |
| **Table S3:** Shared regions from the Welsh pony genome-wide association analyses (cont.) | | | | | | | | |
|  |  |  | **All WP** | | **Section ABCD WP** | | **Section AB WP** | |
| **Trait** | **Chr** | **Total**  **GWAS** | **Min_SNP** | **Max_SNP** | **Min_SNP** | **Max_SNP** | **Min_SNP** | **Max_SNP** |
| **LAM (cont.)** | 15 | 2 | 49986709 | 50013578 | - | - | 49986709 | 50013578 |
|  | 16 | 2 | 65111190 | NA | - | - | 64888181 | 64938437 |

Shared regions from the Welsh pony (WP) genome-wide association analyses (GWA) including the full cohort (n=264), individuals identified by pedigree as section A, B, C or D (n=238) and individuals identified by pedigree as section A or B (n=220). Bolded values are regions which met the criteria for being considered a region of interest (ROI). Values indicated as NA for the maximum SNP are those in which a single SNP exceeded suggestive or genome-wide significant threshold; values indicated as a dash (-) for both minimum and maximum SNP are those in which GWA did not identify the region as shared in that cohort. Highlighted chromosomes (Chr) indicate regions which were shared with several traits.

| **Table S4:** Prioritization of the GWA results of the full Welsh pony cohort based on fixed-size regions | | | | | | | | | |
| --- | --- | --- | --- | --- | --- | --- | --- | --- | --- |
| **Trait** | **Chr** | **Summary** | **Min_SNP** | **Max_SNP** | **Sugg_SNPs** | **Sign_SNPs** | **Min_Region** | **Max_Region** | **Total_Genes** |
| **Insulin** | 1 |  | 46119989 | NA | 1 | 0 | 45619989 | 46619989 | 3 |
|  | 5 | **H** | 40632818 | 41895313 | 86 | 4 | 40132818 | 42395313 | 76 |
|  | 6 | **A** | 82238815 | 82729921 | 11 | 0 | 81738815 | 83229921 | 17 |
|  | 8 |  | 75410291 | 75771110 | 6 | 0 | 74910291 | 76271110 | 14 |
|  | 9 |  | 83981022 | 84014912 | 6 | 0 | 83481022 | 84514912 | 49 |
|  | 13 |  | 14234078 | 14849603 | 7 | 0 | 13734078 | 15349603 | 9 |
|  | 13 |  | 37700109 | 37723843 | 3 | 1 | 37200109 | 38223843 | 1 |
|  | 14 |  | 31226680 | 31583686 | 4 | 0 | 30726680 | 32083686 | 14 |
|  | 15 | **A H** | 5748638 | 6140956 | 28 | 5 | 5248638 | 6640956 | 2 |
|  | 15 |  | 54081224 | 54559632 | 5 | 1 | 53581224 | 55059632 | 19 |
|  | 19 |  | 9606463 | 9637331 | 4 | 3 | 9106463 | 10137331 | 3 |
|  | 23 |  | 46084858 | 46952228 | 6 | 3 | 45584858 | 47452228 | 6 |
|  | 24 | **H** | 28580621 | 29056428 | 15 | 7 | 28080621 | 29556428 | 6 |
|  | 34 |  | 38174280 | NA | 1 | 0 | 37674280 | 38674280 | 17 |
|  | 28 | **F** | 38543945 | NA | 1 | 0 | 38043945 | 39043945 | 23 |
| **Insulin-OST** | 1 |  | 119102659 | 119140428 | 6 | 0 | 118602659 | 119640428 | 13 |
|  | 8 | **A** | 73418239 | 73458142 | 4 | 0 | 72918239 | 73958142 | 5 |
|  | 9 |  | 27879884 | NA | 1 | 0 | 27379884 | 28379884 | 13 |
|  | 9 |  | 28468074 | NA | 1 | 0 | 27968074 | 28968074 | 15 |
|  | 20 |  | 59181583 | 59182258 | 2 | 0 | 58681583 | 59682258 | 5 |
|  | 28 | **F H** | 39385975 | 39462810 | 4 | 2 | 38885975 | 39962810 | 44 |
|  | 31 |  | 8856537 | 8855069 | 2 | 0 | 8356537 | 9355069 | 18 |
| **Glucose** | 3 |  | 32093888 | 32098312 | 4 | 0 | 31593888 | 32598312 | 21 |
|  | 8 | **C** | 84913969 | 85008392 | 2 | 2 | 84413969 | 85508392 | 3 |
|  | 15 |  | 83778178 | NA | 1 | 1 | 83278178 | 84278178 | 14 |
|  | 16 |  | 86563618 | 86743699 | 2 | 0 | 86063618 | 87243699 | 6 |
|  | 22 |  | 42270349 | 42320092 | 2 | 0 | 41770349 | 42820092 | 10 |
|  | 28 |  | 14969841 | 15045427 | 2 | 1 | 14469841 | 15545427 | 6 |
|  | 28 |  | 33576312 | NA | 1 | 0 | 33076312 | 34076312 | 10 |
| **Table S4:** Prioritization of the GWA results of the full Welsh pony cohort based on fixed-size regions (cont.) | | | | | | | | | |
| **Trait** | **Chr** | **Summary** | **Min_SNP** | **Max_SNP** | **Sugg_SNPs** | **Sign_SNPs** | **Min_Region** | **Max_Region** | **Total_Genes** |
| **Glucose (cont.)** | 29 | **A** | 22555245 | 22557916 | 2 | 0 | 22055245 | 23057916 | 15 |
| **Glucose-OST** | 1 |  | 185361795 | NA | 1 | 0 | 184861795 | 185861795 | 9 |
|  | 4 |  | 8502301 | 8522723 | 4 | 0 | 8002301 | 9022723 | 10 |
|  | 4 |  | 40150197 | 40352671 | 11 | 4 | 39650197 | 40852671 | 12 |
|  | 7 |  | 76975314 | NA | 1 | 0 | 76475314 | 77475314 | 41 |
|  | 23 | **C** | 10182647 | 10226427 | 4 | 2 | 9682647 | 10726427 | 5 |
| **NEFA** | 4 |  | 14831152 | 14840371 | 4 | 0 | 14331152 | 15340371 | 24 |
|  | 6 |  | 68206430 | 68512033 | 2 | 0 | 67706430 | 69012033 | 36 |
|  | 6 | **C** | 77102911 | NA | 1 | 0 | 76602911 | 77602911 | 6 |
|  | 8 |  | 11128642 | 11158885 | 2 | 0 | 10628642 | 11658885 | 12 |
|  | 8 |  | 69737476 | NA | 1 | 0 | 69237476 | 70237476 | 11 |
|  | 19 |  | 1055718 | NA | 1 | 1 | 555718 | 1555718 | 11 |
|  | 20 |  | 8830210 | NA | 1 | 0 | 8330210 | 9330210 | 9 |
|  | 20 |  | 26078001 | NA | 1 | 0 | 25578001 | 26578001 | 31 |
|  | 22 | **C** | 19009107 | NA | 1 | 0 | 18509107 | 19509107 | 13 |
|  | 31 |  | 13902942 | NA | 1 | 0 | 13402942 | 14402942 | 6 |
| **TG** | 1 |  | 153409995 | 153700953 | 2 | 0 | 152909995 | 154200953 | 6 |
|  | 4 |  | 93870436 | NA | 1 | 0 | 93370436 | 94370436 | 16 |
|  | 12 | **C** | 35927778 | 35956541 | 4 | 1 | 35427778 | 36456541 | 45 |
| **Adiponectin** | 17 |  | 61546409 | 61552964 | 3 | 0 | 61046409 | 62052964 | 9 |
|  | 18 | **H** | 60290699 | 60393507 | 10 | 5 | 59790699 | 60893507 | 12 |
| **Leptin** | 1 |  | 72370796 | 73160541 | 31 | 1 | 71870796 | 73660541 | 12 |
|  | 5 |  | 43015591 | 43412260 | 24 | 0 | 42515591 | 43912260 | 56 |
|  | 7 | **A H** | 67955613 | 67964668 | 5 | 4 | 67455613 | 68464668 | 4 |
|  | 10 | **C H** | 866333 | 884264 | 3 | 1 | 366333 | 1384264 | 14 |
|  | 21 | **H** | 22944751 | 23022779 | 2 | 1 | 22444751 | 23522779 | 1 |
|  | 26 |  | 11291558 | NA | 1 | 0 | 10791558 | 11791558 | 36 |
|  | 28 |  | 36456338 | 36459615 | 3 | 0 | 35956338 | 36959615 | 21 |
| **ACTH** | 1 | **A H** | 70266479 | 70832972 | 20 | 1 | 69766479 | 71332972 | 25 |
| **Table S4:** Prioritization of the GWA results of the full Welsh pony cohort based on fixed-size regions (cont.) | | | | | | | | | |
| **Trait** | **Chr** | **Summary** | **Min_SNP** | **Max_SNP** | **Sugg_SNPs** | **Sign_SNPs** | **Min_Region** | **Max_Region** | **Total_Genes** |
| **ACTH (cont.)** | 5 | **A** | 16869826 | 17349383 | 30 | 4 | 16369826 | 17849383 | 3 |
|  | 10 | **H** | 55658306 | 56077011 | 2 | 1 | 55158306 | 56577011 | 27 |
|  | 10 | **C H** | 80023665 | NA | 1 | 0 | 79523665 | 80523665 | 6 |
|  | 18 |  | 4529063 | NA | 1 | 0 | 4029063 | 5029063 | 18 |
|  | 19 | **C** | 24243287 | 24246621 | 2 | 0 | 23743287 | 24746621 | 20 |
|  | 19 |  | 33315383 | 33342063 | 4 | 0 | 32815383 | 33842063 | 11 |
|  | 20 |  | 63684506 | NA | 1 | 0 | 63184506 | 64184506 | 93 |
|  | 21 |  | 264658 | 2467359 | 12 | 0 | -235342 | 2967359 | 25 |
|  | 25 |  | 26250218 | NA | 1 | 0 | 25750218 | 26750218 | 22 |
| **NH** | 1 |  | 91537471 | 91969415 | 11 | 0 | 91037471 | 92469415 | 14 |
|  | 4 |  | 62017772 | 62060721 | 2 | 2 | 61517772 | 62560721 | 6 |
|  | 4 | **C F G** | 68114618 | 68576476 | 2 | 0 | 67614618 | 69076476 | 6 |
|  | 4 | **C** | 77390519 | NA | 1 | 0 | 76890519 | 77890519 | 14 |
|  | 4 | **C** | 78314683 | 78699729 | 3 | 1 | 77814683 | 79199729 | 22 |
|  | 4 |  | 79698145 | 80390074 | 14 | 7 | 79198145 | 80890074 | 8 |
|  | 6 | **F** | 1019810 | 1033178 | 3 | 0 | 519810 | 1533178 | 7 |
|  | 7 | **E** | 93233594 | 93628623 | 10 | 6 | 92733594 | 94128623 | 9 |
|  | 8 | **C** | 64510733 | 64609130 | 2 | 1 | 64010733 | 65109130 | 6 |
|  | 8 |  | 88125499 | 88327659 | 2 | 1 | 87625499 | 88827659 | 13 |
|  | 9 | **H** | 33913440 | 35808721 | 39 | 9 | 33413440 | 36308721 | 55 |
|  | 11 | **E H** | 19050799 | 19240093 | 11 | 8 | 18550799 | 19740093 | 5 |
|  | 12 |  | 7654801 | 7676262 | 2 | 0 | 7154801 | 8176262 | 18 |
|  | 12 |  | 15601877 | NA | 1 | 0 | 15101877 | 16101877 | 7 |
|  | 14 | **H** | 63736228 | 63834285 | 7 | 3 | 63236228 | 64334285 | 25 |
|  | 20 | **H** | 40661395 | 41066022 | 10 | 4 | 40161395 | 41566022 | 3 |
|  | 20 | **E H** | 60832063 | 61575820 | 11 | 2 | 60332063 | 62075820 | 23 |
|  | 21 | **A E H** | 20812917 | 22117426 | 22 | 13 | 20312917 | 22617426 | 16 |
|  | 24 |  | 21429112 | 21604747 | 3 | 0 | 20929112 | 22104747 | 29 |
|  | 24 | **H** | 33796794 | 35472785 | 62 | 33 | 33296794 | 35972785 | 11 |
| **Table S4:** Prioritization of the GWA results of the full Welsh pony cohort based on fixed-size regions (cont.) | | | | | | | | | |
| **Trait** | **Chr** | **Summary** | **Min_SNP** | **Max_SNP** | **Sugg_SNPs** | **Sign_SNPs** | **Min_Region** | **Max_Region** | **Total_Genes** |
| **NH (cont.)** | 27 |  | 14461113 | 14463955 | 2 | 0 | 13961113 | 14963955 | 7 |
|  | 29 |  | 33232105 | 33233161 | 2 | 0 | 32732105 | 33733161 | 20 |
| **GH** | 1 | **C** | 120905261 | NA | 1 | 0 | 120405261 | 121405261 | 25 |
|  | 1 | **H** | 132203667 | 133711337 | 30 | 14 | 131703667 | 134211337 | 12 |
|  | 1 |  | 150735268 | NA | 1 | 0 | 150235268 | 151235268 | 8 |
|  | 4 |  | 67153317 | 67163513 | 2 | 0 | 66653317 | 67663513 | 5 |
|  | 4 | **C F G** | 68576476 | NA | 1 | 0 | 68076476 | 69076476 | 49 |
|  | 4 | **A H** | 84285316 | 85497218 | 63 | 33 | 83785316 | 85997218 | 9 |
|  | 6 | **F** | 1019810 | 1154034 | 4 | 3 | 519810 | 1654034 | 6 |
|  | 7 | **E** | 93233594 | 93580126 | 7 | 4 | 92733594 | 94080126 | 13 |
|  | 9 |  | 55626969 | 55685330 | 2 | 0 | 55126969 | 56185330 | 17 |
|  | 10 |  | 3673095 | 3673552 | 2 | 0 | 3173095 | 4173552 | 3 |
|  | 10 |  | 32529022 | 32559811 | 4 | 0 | 32029022 | 33059811 | 59 |
|  | 11 | **E H** | 18827291 | 19240093 | 14 | 9 | 18327291 | 19740093 | 29 |
|  | 12 |  | 25641997 | NA | 1 | 0 | 25141997 | 26141997 | 24 |
|  | 15 |  | 15062753 | 15656836 | 12 | 4 | 14562753 | 16156836 | 4 |
|  | 19 | **H** | 31283482 | 31445588 | 9 | 1 | 30783482 | 31945588 | 53 |
|  | 20 | **A** | 30141925 | 30449510 | 12 | 4 | 29641925 | 30949510 | 6 |
|  | 20 | **H** | 64731849 | 64861251 | 12 | 7 | 64231849 | 65361251 | 7 |
|  | 21 | **C E** | 22007711 | NA | 2 | 0 | 21507711 | 22507711 | 5 |
|  | 22 | **H** | 41033715 | 41065262 | 4 | 4 | 40533715 | 41565262 | 6 |
|  | 24 |  | 18072215 | 18172937 | 4 | 0 | 17572215 | 18672937 | 17 |
|  | 25 |  | 19485041 | NA | 1 | 1 | 18985041 | 19985041 | 7 |
|  | 31 |  | 17898824 | 17912707 | 4 | 0 | 17398824 | 18412707 | 4 |
|  | 31 |  | 18700158 | 18819670 | 4 | 0 | 18200158 | 19319670 | 22 |
| **LAM** | 1 | **C H** | 49441032 | NA | 1 | 1 | 48941032 | 49941032 | 27 |
|  | 2 | **A H** | 36123836 | 36633565 | 14 | 11 | 35623836 | 37133565 | 3 |
|  | 5 |  | 79658109 | NA | 1 | 0 | 79158109 | 80158109 | 7 |
|  | 7 |  | 97437120 | 97439429 | 2 | 0 | 96937120 | 97939429 | 10 |
| **Table S4:** Prioritization of the GWA results of the full Welsh pony cohort based on fixed-size regions (cont.) | | | | | | | | | |
| **Trait** | **Chr** | **Summary** | **Min_SNP** | **Max_SNP** | **Sugg_SNPs** | **Sign_SNPs** | **Min_Region** | **Max_Region** | **Total_Genes** |
| **LAM (cont.)** | 10 |  | 64224504 | 64281425 | 2 | 0 | 63724504 | 64781425 | 16 |
|  | 13 |  | 27143211 | 27035221 | 5 | 0 | 26643211 | 27535221 | 8 |
|  | 14 |  | 52578019 | 52579053 | 2 | 0 | 52078019 | 53079053 | 3 |
|  | 15 | **C H** | 50978261 | 51005138 | 3 | 1 | 50478261 | 51505138 | 7 |
|  | 15 |  | 64654206 | 64769743 | 12 | 0 | 64154206 | 65269743 | 9 |
|  | 16 |  | 35123235 | 35595543 | 3 | 0 | 34623235 | 36095543 | 8 |
|  | 16 | **C** | 66694166 | NA | 1 | 0 | 66194166 | 67194166 | 24 |
|  | 19 |  | 39125743 | 39626653 | 11 | 1 | 38625743 | 40126653 | 24 |
|  | 19 | **H** | 59885237 | 61849890 | 78 | 35 | 59385237 | 62349890 | 4 |
|  | 20 | **E** | 62018962 | 62085163 | 4 | 3 | 61518962 | 62585163 | 10 |
|  | 22 | **B** | 3551367 | NA | 1 | 0 | 3051367 | 4051367 | 37 |
|  | 25 |  | 32816803 | 32852556 | 2 | 0 | 32316803 | 33352556 | 5 |
|  | 28 | **H** | 10461982 | 10666731 | 19 | 7 | 9961982 | 11166731 | 8 |
|  | 31 |  | 10611327 | 10509324 | 5 | 3 | 10111327 | 11009324 | 3 |

Provided in the table is the base pair position of the lowest (Min_SNP) and highest (Max_SNP) SNP in the region, the min (Min_Region) and maximum (Max_Region) boundaries of the region based on a fixed value of 500Kb 5’ of the Min_SNP and 500Kb 3’ of the Max_SNP, as well as the number of SNPs per region which exceeded the suggestive (Sugg_SNPs) and genome-wide significance (Sign_SNPs) threshold. Total_Genes includes all protein-coding, pseudogenes, and RNA genes identified for the region based on EquCab3. A black box in the summary column indicates the region did not meet the criteria to be considered an ROI and was not shared with another GWA cohort or trait. Letters in the summary column represent: (A) region was shared with another Welsh pony cohort and at least one region was considered an ROI, (B) region was shared with the Morgans and at least one region was considered an ROI, (C) region was shared with another Welsh pony cohort but neither regions met the criteria for an ROI, (D) region was shared with Morgan but neither regions met the criteria for an ROI, (E) region was shared with another trait in this cohort and at least one region was considered an ROI, (F) region was shared with another trait in this cohort but no regions met the criteria for an ROI, (G) region was shared across multiple traits in another cohort, (H) region was identified as shared across breeds on metanalysis and was considered an MA-ROI. Highlighted chromosomes (Chr) indicate regions which were shared with several traits.

| **Table S5:** Prioritization of the GWA results of the section A, B, C and D Welsh ponies based on fixed-size regions | | | | | | | | | |
| --- | --- | --- | --- | --- | --- | --- | --- | --- | --- |
| **Trait** | **Chr** | **Summary** | **Min_SNP** | **Max_SNP** | **Sugg_SNPs** | **Sign_SNPs** | **Min_Region** | **Max_Region** | **Total_Genes** |
| **Insulin** | 1 |  | 88317885 | NA | 1 | 0 | 87817885 | 88817885 | 2 |
|  | 4 |  | 16025941 | 16029966 | 4 | 0 | 15525941 | 16529966 | 17 |
|  | 6 | **A** | 82585066 | 83826234 | 32 | 2 | 82085066 | 84326234 | 26 |
|  | 9 | **A** | 61021946 | 61144885 | 13 | 3 | 60521946 | 61644885 | 7 |
|  | 12 |  | 859517 | NA | 1 | 0 | 359517 | 1359517 | 14 |
|  | 12 |  | 5811391 | 5812573 | 3 | 0 | 5311391 | 6312573 | 3 |
|  | 17 |  | 11193396 | 11343933 | 4 | 0 | 10693396 | 11843933 | 9 |
|  | 18 |  | 79126354 | 79216656 | 3 | 1 | 78626354 | 79716656 | 10 |
|  | 21 |  | 34649027 | NA | 1 | 0 | 34149027 | 35149027 | 3 |
|  | 24 |  | 38174280 | NA | 1 | 0 | 37674280 | 38674280 | 17 |
| **Insulin-OST** | 1 |  | 176823704 | NA | 1 | 0 | 176323704 | 177323704 | 18 |
|  | 1 |  | 181205641 | NA | 1 | 0 | 180705641 | 181705641 | 3 |
|  | 3 | **C** | 67119398 | NA | 1 | 0 | 66619398 | 67619398 | 14 |
|  | 5 |  | 89878763 | NA | 1 | 0 | 89378763 | 90378763 | 9 |
|  | 6 | **C F G** | 15257536 | 15267456 | 3 | 1 | 14757536 | 15767456 | 12 |
|  | 10 | **B C H** | 73334761 | 73417042 | 3 | 0 | 72834761 | 73917042 | 6 |
|  | 31 |  | 5253579 | NA | 1 | 0 | 4753579 | 5753579 | 7 |
| **Glucose** | 2 |  | 88732913 | 88775982 | 2 | 0 | 88232913 | 89275982 | 6 |
|  | 4 |  | 57433023 | 57463516 | 2 | 1 | 56933023 | 57963516 | 20 |
|  | 8 | **C** | 84774486 | 84918226 | 5 | 0 | 84274486 | 85418226 | 3 |
|  | 8 |  | 92368897 | NA | 1 | 0 | 91868897 | 92868897 | 12 |
|  | 15 |  | 71112709 | NA | 1 | 0 | 70612709 | 71612709 | 10 |
|  | 19 |  | 8738837 | 8781089 | 2 | 0 | 8238837 | 9281089 | 6 |
|  | 29 |  | 4401376 | 4855454 | 4 | 1 | 3901376 | 5355454 | 7 |
| **Glucose-OST** | 5 | **C** | 63041759 | 63549216 | 12 | 0 | 62541759 | 64049216 | 16 |
|  | 23 | **C** | 10217644 | 10226427 | 2 | 0 | 9717644 | 10726427 | 4 |
|  | 28 |  | 14969841 | NA | 1 | 0 | 14469841 | 15469841 | 6 |
|  | 28 | **A E G H** | 34960948 | NA | 1 | 1 | 34460948 | 35460948 | 23 |
| **NEFA** | 6 | **C** | 77102911 | NA | 1 | 0 | 76602911 | 77602911 | 6 |
| **Table S5:** Prioritization of the GWA results of the section A, B, C and D Welsh ponies based on fixed-size regions (cont.) | | | | | | | | | |
| **Trait** | **Chr** | **Summary** | **Min_SNP** | **Max_SNP** | **Sugg_SNPs** | **Sign_SNPs** | **Min_Region** | **Max_Region** | **Total_Genes** |
| **NEFA (cont.)** | 7 | **C** | 7744001 | 7858148 | 2 | 0 | 7244001 | 8358148 | 9 |
|  | 7 | **C** | 8657121 | 8718823 | 3 | 0 | 8157121 | 9218823 | 3 |
|  | 9 | **A** | 48687570 | 50301924 | 74 | 5 | 48187570 | 50801924 | 32 |
|  | 13 |  | 3866723 | NA | 1 | 0 | 3366723 | 4366723 | 14 |
|  | 14 | **C** | 33187338 | NA | 1 | 0 | 32687338 | 33687338 | 7 |
|  | 22 | **C** | 19009107 | NA | 1 | 0 | 18509107 | 19509107 | 13 |
|  | 28 | **A E G** | 34777499 | 35488520 | 25 | 6 | 34277499 | 35988520 | 43 |
|  | 31 |  | 9275456 | 8325401 | 9 | 0 | 8775456 | 8825401 | 0 |
|  | 32 |  | 21391267 | 21497776 | 2 | 0 | 20891267 | 21997776 | 0 |
| **TG** | 7 |  | 28031826 | 28039745 | 2 | 0 | 27531826 | 28539745 | 22 |
|  | 12 | **C** | 35945816 | 35956541 | 2 | 0 | 35445816 | 36456541 | 45 |
|  | 17 |  | 17532266 | NA | 2 | 0 | 17032266 | 18032266 | 4 |
|  | 17 |  | 33912651 | NA | 1 | 0 | 33412651 | 34412651 | 5 |
|  | 20 | **C** | 56719186 | 56815453 | 4 | 1 | 56219186 | 57315453 | 2 |
| **Adiponectin** | 1 |  | 175782149 | 177072407 | 32 | 1 | 175282149 | 177572407 | 23 |
|  | 7 |  | 75100837 | NA | 1 | 0 | 74600837 | 75600837 | 54 |
|  | 20 |  | 8415408 | 9139191 | 5 | 0 | 7915408 | 9639191 | 19 |
|  | 22 | **C** | 37875269 | 37957795 | 3 | 0 | 37375269 | 38457795 | 20 |
|  | 25 |  | 9125953 | NA | 1 | 0 | 8625953 | 9625953 | 6 |
|  | 28 | **F** | 41052952 | NA | 1 | 0 | 40552952 | 41552952 | 18 |
| **Leptin** | 2 |  | 87434404 | NA | 1 | 0 | 86934404 | 87934404 | 13 |
|  | 4 | **F** | 48014169 | 48031048 | 6 | 0 | 47514169 | 48531048 | 7 |
|  | 6 |  | 2348093 | 2376386 | 6 | 1 | 1848093 | 2876386 | 3 |
|  | 6 |  | 21686436 | 22141052 | 9 | 0 | 21186436 | 22641052 | 10 |
|  | 7 | **A H** | 67955613 | 67964668 | 2 | 0 | 67455613 | 68464668 | 4 |
|  | 8 |  | 87412707 | NA | 1 | 0 | 86912707 | 87912707 | 6 |
|  | 10 | **C H** | 857433 | 884264 | 4 | 0 | 357433 | 1384264 | 14 |
|  | 10 |  | 83363991 | NA | 1 | 0 | 82863991 | 83863991 | 15 |
|  | 10 |  | 84395615 | NA | 1 | 0 | 83895615 | 84895615 | 8 |
| **Table S5:** Prioritization of the GWA results of the section A, B, C and D Welsh ponies based on fixed-size regions (cont.) | | | | | | | | | |
| **Trait** | **Chr** | **Summary** | **Min_SNP** | **Max_SNP** | **Sugg_SNPs** | **Sign_SNPs** | **Min_Region** | **Max_Region** | **Total_Genes** |
| **Leptin (cont.)** | 11 |  | 33592865 | NA | 1 | 0 | 33092865 | 34092865 | 40 |
|  | 12 |  | 25937470 | 25914980 | 2 | 0 | 25437470 | 26414980 | 28 |
|  | 13 |  | 7690179 | NA | 1 | 0 | 7190179 | 8190179 | 30 |
|  | 15 |  | 24814175 | 24816607 | 2 | 0 | 24314175 | 25316607 | 3 |
|  | 16 |  | 42665270 | NA | 1 | 0 | 42165270 | 43165270 | 14 |
|  | 26 |  | 11291558 | 11425566 | 4 | 1 | 10791558 | 11925566 | 2 |
|  | 28 | **F** | 40504716 | NA | 1 | 0 | 40004716 | 41004716 | 23 |
| **ACTH** | 1 | **A F** | 44391917 | 44627074 | 13 | 0 | 43891917 | 45127074 | 2 |
|  | 3 | **D** | 44073772 | 44105888 | 8 | 0 | 43573772 | 44605888 | 6 |
|  | 5 | **A** | 17101043 | 17252354 | 9 | 0 | 16601043 | 17752354 | 19 |
|  | 10 | **C H** | 78845710 | NA | 1 | 1 | 78345710 | 79345710 | 10 |
|  | 15 |  | 13711487 | NA | 1 | 0 | 13211487 | 14211487 | 11 |
|  | 19 | **C** | 24243287 | 24246621 | 2 | 0 | 23743287 | 24746621 | 18 |
|  | 19 | **E** | 37642432 | NA | 1 | 0 | 37142432 | 38142432 | 8 |
|  | 20 | **E** | 60431850 | NA | 3 | 0 | 59931850 | 60931850 | 0 |
|  | 24 | **C G** | 39497717 | NA | 1 | 0 | 38997717 | 39997717 | 14 |
|  | 30 |  | 1302176 | 1304866 | 4 | 0 | 802176 | 1804866 | 13 |
| **NH** | 1 | **F** | 44398249 | NA | 1 | 0 | 43898249 | 44898249 | 2 |
|  | 3 |  | 109783963 | NA | 1 | 0 | 109283963 | 110283963 | 11 |
|  | 4 | **A E G** | 69118549 | 69714717 | 17 | 14 | 68618549 | 70214717 | 7 |
|  | 4 |  | 72715285 | 73055056 | 2 | 0 | 72215285 | 73555056 | 5 |
|  | 4 | **C G** | 76437287 | 77891737 | 17 | 0 | 75937287 | 78391737 | 14 |
|  | 4 | **E** | 83194842 | 85546563 | 54 | 38 | 82694842 | 86046563 | 67 |
|  | 8 | **C** | 64510733 | 64548459 | 2 | 0 | 64010733 | 65048459 | 9 |
|  | 14 |  | 57777287 | NA | 1 | 0 | 57277287 | 58277287 | 6 |
|  | 15 |  | 44638315 | NA | 1 | 3 | 44138315 | 45138315 | 1 |
|  | 15 |  | 73207370 | 73338081 | 5 | 3 | 72707370 | 73838081 | 13 |
|  | 16 |  | 19756213 | 20195426 | 10 | 0 | 19256213 | 20695426 | 12 |
|  | 16 |  | 26012920 | NA | 1 | 0 | 25512920 | 26512920 | 8 |
| **Table S5:** Prioritization of the GWA results of the section A, B, C and D Welsh ponies based on fixed-size regions (cont.) | | | | | | | | | |
| **Trait** | **Chr** | **Summary** | **Min_SNP** | **Max_SNP** | **Sugg_SNPs** | **Sign_SNPs** | **Min_Region** | **Max_Region** | **Total_Genes** |
| **NH (cont.)** | 17 |  | 36798348 | 36856368 | 9 | 9 | 36298348 | 37356368 | 5 |
|  | 18 |  | 68917978 | NA | 1 | 1 | 68417978 | 69417978 | 5 |
|  | 20 | **F** | 30160893 | NA | 1 | 1 | 29660893 | 30660893 | 42 |
|  | 21 |  | 6664894 | 6835706 | 5 | 2 | 6164894 | 7335706 | 11 |
|  | 21 | **A F G H** | 20812917 | 21679286 | 4 | 0 | 20312917 | 22179286 | 19 |
|  | 24 |  | 8561717 | 11119679 | 17 | 0 | 8061717 | 11619679 | 43 |
|  | 24 |  | 30419181 | 30419482 | 2 | 0 | 29919181 | 30919482 | 2 |
|  | 26 |  | 13669849 | NA | 1 | 0 | 13169849 | 14169849 | 7 |
|  | 29 |  | 12571950 | 12620905 | 5 | 0 | 12071950 | 13120905 | 11 |
|  | 30 |  | 30980395 | NA | 1 | 0 | 30480395 | 31480395 | 26 |
| **GH** | 1 |  | 73428660 | 73434597 | 2 | 0 | 72928660 | 73934597 | 2 |
|  | 1 | **C** | 120654637 | 120684527 | 5 | 0 | 120154637 | 121184527 | 22 |
|  | 4 | **F** | 46793329 | 47851529 | 10 | 0 | 46293329 | 48351529 | 10 |
|  | 4 |  | 52253860 | NA | 1 | 0 | 51753860 | 52753860 | 9 |
|  | 4 |  | 61808230 | 62195564 | 3 | 0 | 61308230 | 62695564 | 17 |
|  | 4 | **A E G** | 69236860 | 69660028 | 5 | 0 | 68736860 | 70160028 | 6 |
|  | 4 |  | 74331018 | 76480368 | 41 | 7 | 73831018 | 76980368 | 25 |
|  | 4 |  | 79807650 | 80390074 | 12 | 8 | 79307650 | 80890074 | 22 |
|  | 4 | **A E H** | 84044345 | 85375688 | 34 | 18 | 83544345 | 85875688 | 56 |
|  | 5 |  | 20984165 | NA | 1 | 0 | 20484165 | 21484165 | 8 |
|  | 9 |  | 75259988 | 75263736 | 2 | 1 | 74759988 | 75763736 | 10 |
|  | 10 |  | 51267938 | NA | 1 | 0 | 50767938 | 51767938 | 9 |
|  | 11 |  | 15497051 | 16248131 | 36 | 26 | 14997051 | 16748131 | 40 |
|  | 13 |  | 1235274 | 531380 | 29 | 19 | 735274 | 1031380 | 8 |
|  | 14 |  | 5739617 | 6265883 | 11 | 0 | 5239617 | 6765883 | 20 |
|  | 15 |  | 85423316 | NA | 1 | 0 | 84923316 | 85923316 | 27 |
|  | 16 |  | 27103995 | 28231661 | 41 | 11 | 26603995 | 28731661 | 17 |
|  | 16 |  | 88165628 | 88202104 | 5 | 0 | 87665628 | 88702104 | 10 |
|  | 17 |  | 167021 | NA | 1 | 0 | -332979 | 667021 | 12 |
| **Table S5:** Prioritization of the GWA results of the section A, B, C and D Welsh ponies based on fixed-size regions (cont.) | | | | | | | | | |
| **Trait** | **Chr** | **Summary** | **Min_SNP** | **Max_SNP** | **Sugg_SNPs** | **Sign_SNPs** | **Min_Region** | **Max_Region** | **Total_Genes** |
| **GH (cont.)** | 17 |  | 57101997 | NA | 1 | 0 | 56601997 | 57601997 | 8 |
|  | 18 |  | 70918093 | NA | 1 | 0 | 70418093 | 71418093 | 4 |
|  | 18 |  | 75058371 | 76075236 | 26 | 6 | 74558371 | 76575236 | 31 |
|  | 18 |  | 80391110 | 81050756 | 27 | 1 | 79891110 | 81550756 | 25 |
|  | 20 | **A F** | 30141925 | 30160893 | 3 | 1 | 29641925 | 30660893 | 42 |
|  | 20 | **E** | 60935600 | 61788330 | 8 | 1 | 60435600 | 62288330 | 3 |
|  | 21 |  | 18238312 | 18263289 | 2 | 0 | 17738312 | 18763289 | 15 |
|  | 21 | **C F G** | 21539433 | 21542349 | 2 | 0 | 21039433 | 22042349 | 9 |
|  | 22 |  | 43609456 | NA | 1 | 0 | 43109456 | 44109456 | 6 |
|  | 24 |  | 22090203 | 22552582 | 9 | 0 | 21590203 | 23052582 | 32 |
|  | 25 |  | 25896326 | 25897963 | 2 | 0 | 25396326 | 26397963 | 16 |
| **LAM** | 1 | **C H** | 49441032 | NA | 1 | 0 | 48941032 | 49941032 | 22 |
|  | 2 |  | 29737934 | 29777141 | 17 | 7 | 29237934 | 30277141 | 33 |
|  | 2 | **A H** | 36322824 | 36633565 | 8 | 0 | 35822824 | 37133565 | 27 |
|  | 11 |  | 37530491 | 37555597 | 2 | 0 | 37030491 | 38055597 | 33 |
|  | 16 |  | 9221468 | NA | 1 | 0 | 8721468 | 9721468 | 10 |
|  | 17 |  | 46013130 | 46020667 | 3 | 0 | 45513130 | 46520667 | 10 |
|  | 18 |  | 26676637 | NA | 1 | 0 | 26176637 | 27176637 | 2 |
|  | 19 | **E** | 37272294 | 37328619 | 15 | 3 | 36772294 | 37828619 | 10 |
|  | 20 |  | 48609221 | NA | 1 | 0 | 48109221 | 49109221 | 15 |
|  | 23 |  | 4297964 | 4341498 | 5 | 0 | 3797964 | 4841498 | 8 |
|  | 27 |  | 4129106 | NA | 1 | 0 | 3629106 | 4629106 | 10 |
|  | 30 |  | 12607858 | 13128439 | 15 | 9 | 12107858 | 13628439 | 15 |

Provided in the table is the base pair position of the lowest (Min_SNP) and highest (Max_SNP) SNP in the region, the min (Min_Region) and maximum (Max_Region) boundaries of the region based on a fixed value of 500Kb 5’ of the Min_SNP and 500Kb 3’ of the Max_SNP, as well as the number of SNPs per region which exceeded the suggestive (Sugg_SNPs) and genome-wide significance (Sign_SNPs) threshold. Total_Genes includes all protein-coding, pseudogenes, and RNA genes identified for the region based on EquCab3. A black box in the summary column indicates the region did not meet the criteria to be considered an ROI and was not shared with another GWA cohort or trait. Letters in the summary column represent: (A) region was shared with another Welsh pony cohort and at least one region was considered an ROI, (B) region was shared with the Morgans and at least one region was considered an ROI, (C) region was shared with another Welsh pony cohort but neither regions met the criteria for an ROI, (D) region was shared with Morgan but neither regions met the criteria for an ROI, (E) region was shared with another trait in this cohort and at least one region was considered an ROI, (F) region was shared with another trait in this cohort but no regions met the criteria for an ROI, (G) region was shared across multiple traits in another cohort, (H) region was identified as shared across breeds on metanalysis and was considered an MA-ROI. Highlighted chromosomes (Chr) indicate regions which were shared with several traits.

| **Table S6:** Prioritization of the GWA results of the section A and B Welsh ponies based on a fixed-size region | | | | | | | | | |
| --- | --- | --- | --- | --- | --- | --- | --- | --- | --- |
| **Trait** | **Chr** | **Summary** | **Min_SNP** | **Max_SNP** | **Sugg_SNPs** | **Sign_SNPs** | **Min_Region** | **Max_Region** | **Total_Genes** |
| **Insulin** | 4 |  | 4672416 | NA | 1 | 0 | 4172416 | 5172416 | 4 |
|  | 8 | **E** | 71911322 | 73187367 | 71 | 48 | 71411322 | 73687367 | 30 |
|  | 9 | **A** | 60513208 | 62048722 | 21 | 6 | 60013208 | 62548722 | 14 |
|  | 10 |  | 72007185 | 72019609 | 2 | 0 | 71507185 | 72519609 | 8 |
|  | 11 |  | 20890216 | NA | 1 | 0 | 20390216 | 21390216 | 53 |
|  | 14 |  | 44171754 | NA | 1 | 0 | 43671754 | 44671754 | 9 |
|  | 15 | **A H** | 5760603 | NA | 1 | 0 | 5260603 | 6260603 | 2 |
|  | 16 |  | 85072895 | 85226499 | 2 | 0 | 84572895 | 85726499 | 23 |
|  | 18 |  | 27406051 | 27434219 | 3 | 0 | 26906051 | 27934219 | 12 |
|  | 21 |  | 37151683 | NA | 1 | 0 | 36651683 | 37651683 | 4 |
| **Insulin-OST** | 3 | **C** | 67827963 | NA | 1 | 0 | 67327963 | 68327963 | 15 |
|  | 6 | **C** | 15257536 | NA | 1 | 0 | 14757536 | 15757536 | 12 |
|  | 8 | **E** | 73418276 | 73458142 | 3 | 0 | 72918276 | 73958142 | 5 |
|  | 9 |  | 51694853 | 52360209 | 26 | 1 | 51194853 | 52860209 | 7 |
|  | 10 | **B C H** | 73415161 | NA | 1 | 0 | 72915161 | 73915161 | 6 |
|  | 23 |  | 33075103 | NA | 2 | 0 | 32575103 | 33575103 | 8 |
| **Glucose** | 2 |  | 78104573 | NA | 1 | 0 | 77604573 | 78604573 | 13 |
|  | 4 |  | 91598735 | 91632300 | 3 | 0 | 91098735 | 92132300 | 6 |
|  | 5 |  | 60653615 | 61066511 | 3 | 1 | 60153615 | 61566511 | 5 |
|  | 8 |  | 89457249 | 90171577 | 19 | 3 | 88957249 | 90671577 | 5 |
|  | 14 |  | 6606837 | 6628311 | 4 | 3 | 6106837 | 7128311 | 13 |
|  | 17 |  | 79020897 | 79997119 | 21 | 4 | 78520897 | 80497119 | 33 |
|  | 29 | **A** | 22523122 | 23217070 | 6 | 5 | 22023122 | 23717070 | 22 |
|  | 31 |  | 7659497 | 7654406 | 3 | 0 | 7159497 | 8154406 | 7 |
| **Glucose-OST** | 5 | **C** | 63447777 | 63549216 | 3 | 0 | 62947777 | 64049216 | 8 |
|  | 16 |  | 86361940 | NA | 1 | 1 | 85861940 | 86861940 | 6 |
|  | 28 | **A E H** | 34434081 | 34960948 | 5 | 1 | 33934081 | 35460948 | 32 |
| **NEFA** | 5 |  | 17682899 | NA | 1 | 0 | 17182899 | 18182899 | 12 |
|  | 6 |  | 74667806 | 74721945 | 4 | 0 | 74167806 | 75221945 | 61 |
| **Table S6:** Prioritization of the GWA results of the section A and B Welsh ponies based on a fixed-size region (cont.) | | | | | | | | | |
| **Trait** | **Chr** | **Summary** | **Min_SNP** | **Max_SNP** | **Sugg_SNPs** | **Sign_SNPs** | **Min_Region** | **Max_Region** | **Total_Genes** |
| **NEFA (cont.)** | 7 |  | 5590146 | NA | 1 | 0 | 5090146 | 6090146 | 42 |
|  | 7 | **C** | 7744001 | NA | 1 | 0 | 7244001 | 8244001 | 8 |
|  | 7 |  | 90384141 | 90387298 | 2 | 0 | 89884141 | 90887298 | 9 |
|  | 9 | **A** | 48876850 | 50428786 | 66 | 10 | 48376850 | 50928786 | 29 |
|  | 14 | **C** | 33144705 | 33289979 | 13 | 0 | 32644705 | 33789979 | 8 |
|  | 18 |  | 21325941 | 22264264 | 2 | 0 | 20825941 | 22764264 | 10 |
|  | 20 |  | 31639261 | NA | 1 | 0 | 31139261 | 32139261 | 20 |
|  | 22 | **C** | 19009107 | 19028315 | 2 | 0 | 18509107 | 19528315 | 14 |
|  | 28 | **A E G** | 34865969 | 34877252 | 7 | 0 | 34365969 | 35377252 | 16 |
| **TG** | 1 |  | 47645272 | NA | 1 | 0 | 47145272 | 48145272 | 1 |
|  | 2 |  | 98328483 | NA | 1 | 0 | 97828483 | 98828483 | 3 |
|  | 4 |  | 88686448 | NA | 1 | 0 | 88186448 | 89186448 | 12 |
|  | 7 |  | 26533379 | 26635921 | 7 | 1 | 26033379 | 27135921 | 26 |
|  | 9 |  | 73409149 | 73438018 | 4 | 0 | 72909149 | 73938018 | 11 |
|  | 20 | **C** | 56347955 | NA | 1 | 0 | 55847955 | 56847955 | 4 |
| **Adiponectin** | 8 |  | 5894342 | NA | 1 | 0 | 5394342 | 6394342 | 20 |
|  | 18 |  | 39196722 | NA | 1 | 0 | 38696722 | 39696722 | 9 |
|  | 20 |  | 26633993 | NA | 1 | 0 | 26133993 | 27133993 | 41 |
|  | 22 | **C** | 37957795 | NA | 1 | 1 | 37457795 | 38457795 | 19 |
| **Leptin** | 10 | **C H** | 872249 | NA | 1 | 0 | 372249 | 1372249 | 14 |
|  | 14 | **F** | 60295756 | NA | 1 | 0 | 59795756 | 60795756 | 6 |
|  | 17 |  | 5633648 | NA | 1 | 1 | 5133648 | 6133648 | 6 |
| **ACTH** | 1 | **A** | 44284734 | 45133993 | 30 | 1 | 43784734 | 45633993 | 5 |
|  | 5 |  | 17101043 | 17252354 | 4 | 0 | 16601043 | 17752354 | 19 |
|  | 10 | **C H** | 79880592 | 80023665 | 2 | 1 | 79380592 | 80523665 | 27 |
|  | 11 |  | 58160240 | NA | 1 | 0 | 57660240 | 58660240 | 29 |
|  | 20 |  | 8331002 | 8355327 | 7 | 0 | 7831002 | 8855327 | 16 |
|  | 24 | **C G** | 38516095 | 39660384 | 23 | 0 | 38016095 | 40160384 | 34 |
| **NH** | 1 |  | 9347701 | NA | 1 | 0 | 8847701 | 9847701 | 13 |
| **Table S6:** Prioritization of the GWA results of the section A and B Welsh ponies based on a fixed-size region (cont.) | | | | | | | | | |
| **Trait** | **Chr** | **Summary** | **Min_SNP** | **Max_SNP** | **Sugg_SNPs** | **Sign_SNPs** | **Min_Region** | **Max_Region** | **Total_Genes** |
| **NH (cont.)** | 3 |  | 69624972 | NA | 1 | 0 | 69124972 | 70124972 | 6 |
|  | 4 | **A G** | 67618110 | 69482711 | 14 | 3 | 67118110 | 69982711 | 16 |
|  | 10 | **F** | 10827320 | NA | 1 | 0 | 10327320 | 11327320 | 47 |
|  | 14 |  | 73473354 | NA | 1 | 0 | 72973354 | 73973354 | 11 |
|  | 21 | **F** | 23990259 | 24995726 | 2 | 0 | 23490259 | 25495726 | 19 |
| **GH** | 1 |  | 166271712 | NA | 1 | 0 | 165771712 | 166771712 | 7 |
|  | 10 | **F** | 11229405 | NA | 1 | 0 | 10729405 | 11729405 | 37 |
|  | 10 |  | 70502635 | 70536766 | 2 | 1 | 70002635 | 71036766 | 13 |
|  | 12 |  | 20064456 | NA | 1 | 0 | 19564456 | 20564456 | 19 |
|  | 17 |  | 27064422 | NA | 1 | 0 | 26564422 | 27564422 | 8 |
|  | 21 | **F** | 23776930 | 23991948 | 5 | 0 | 23276930 | 24491948 | 14 |
|  | 24 |  | 49764166 | NA | 1 | 0 | 49264166 | 50264166 | 0 |
|  | 22 |  | 23930066 | NA | 1 | 0 | 23430066 | 24430066 | 26 |
|  | 25 |  | 15030393 | NA | 1 | 0 | 14530393 | 15530393 | 13 |
| **LAM** | 3 |  | 77977500 | NA | 1 | 0 | 77477500 | 78477500 | 21 |
|  | 8 |  | 45552432 | NA | 1 | 0 | 45052432 | 46052432 | 4 |
|  | 10 |  | 15374259 | 15988198 | 15 | 2 | 14874259 | 16488198 | 73 |
|  | 13 |  | 24882636 | 25740597 | 8 | 2 | 24382636 | 26240597 | 32 |
|  | 14 | **F** | 58930834 | 59667233 | 4 | 0 | 58430834 | 60167233 | 15 |
|  | 15 | **C H** | 50978261 | 51005138 | 3 | 1 | 50478261 | 51505138 | 3 |
|  | 16 | **C** | 66471008 | 66521264 | 2 | 0 | 65971008 | 67021264 | 9 |
|  | 18 |  | 15365144 | NA | 1 | 0 | 14865144 | 15865144 | 4 |
|  | 19 |  | 54249861 | 54263396 | 4 | 0 | 53749861 | 54763396 | 5 |
|  | 20 |  | 43136147 | 43150142 | 3 | 0 | 42636147 | 43650142 | 39 |

Provided in the table is the base pair position of the lowest (Min_SNP) and highest (Max_SNP) SNP in the region, the min (Min_ROI) and maximum (Max_ROI) boundaries of the region based on a fixed value of 500Kb 5’ of the Min_SNP and 500Kb 3’ of the Max_SNP, as well as the number of SNPs per region which exceeded the suggestive (Sugg_SNPs) and genome-wide significance (Sign_SNPs) threshold. The total number of genes includes all protein-coding genes, pseudogenes, and RNA genes identified for region based on EquCab3. A black box in the summary column indicates the region did not meet the criteria to be considered an ROI and was not shared with another GWA cohort or trait. Letters in the summary column represent: (A) region was shared with another Welsh pony cohort GWA and at least one region was considered an ROI, (B) region was shared with the Morgan GWA and at least one region was considered an ROI, (C) region was shared with another Welsh pony cohort GWA but no regions met the criteria for an ROI, (D) region was shared with Morgan GWA but no regions met the criteria for an ROI, (E) region was shared with another trait in this GWA cohort and at least one region was considered an ROI, (F) region was shared with another trait in this GWA cohort but no regions met the criteria for an ROI, (G) region was shared across multiple traits in another GWA cohort, (H) region was identified as shared across breeds on metanalysis and was considered a MA-ROI. Highlighted chromosomes (Chr) indicate regions which were shared with several traits.

| **Table S7:** Final prioritization of GWA results based on a fixed sized region for the Welsh ponies | | | | | | | | | | | | | | | | | | | | |
| --- | --- | --- | --- | --- | --- | --- | --- | --- | --- | --- | --- | --- | --- | --- | --- | --- | --- | --- | --- | --- |
| **High Priority Regions Welsh Ponies Fixed Boundaries** | | | | | | | | | | | | | | | | | | | | |
| **Trait** | | **Chr** | | **Min_Region** | | | **Max_Region** | | | **Protein_Coding** | | | **Pseudogenes** | | | **RNA_Genes** | | | **Total_Genes** | |
| **Insulin** | | 5 | | 40132818 | | | 42395313 | | | 67 | | | 0 | | | 9 | | | 76 | |
|  | | 8 | | 71411322 | | | 73687367 | | | 16 | | | 0 | | | 14 | | | 30 | |
|  | | 15 | | 5248638 | | | 6640956 | | | 0 | | | 0 | | | 2 | | | 2 | |
|  | | 24 | | 28080621 | | | 29556428 | | | 0 | | | 0 | | | 6 | | | 6 | |
| **Insulin-OST** | | 8 | | 72918276 | | | 73958142 | | | 1 | | | 0 | | | 4 | | | 5 | |
|  | | 10 | | 72834761 | | | 73917042 | | | 5 | | | 1 | | | 0 | | | 6 | |
|  | | 28 | | 38885975 | | | 39962810 | | | 42 | | | 0 | | | 2 | | | 44 | |
| **Glucose-OST** | | 28 | | 33934081 | | | 35460948 | | | 26 | | | 0 | | | 6 | | | 32 | |
| **NEFA** | | 9 | | 48187570 | | | 50801924 | | | 19 | | | 0 | | | 13 | | | 32 | |
|  | | 28 | | 34277499 | | | 35988520 | | | 34 | | | 0 | | | 9 | | | 43 | |
| **Adiponectin** | | 18 | | 59790699 | | | 60893507 | | | 8 | | | 0 | | | 4 | | | 12 | |
| **Leptin** | | 7 | | 67455613 | | | 68464668 | | | 4 | | | 0 | | | 0 | | | 4 | |
|  | | 10 | | 366333 | | | 1384264 | | | 2 | | | 0 | | | 12 | | | 14 | |
|  | | 21 | | 22444751 | | | 23522779 | | | 2 | | | 0 | | | 5 | | | 7 | |
| **ACTH** | | 1 | | 69766479 | | | 71332972 | | | 10 | | | 0 | | | 11 | | | 21 | |
|  | | 3 | | 43573772 | | | 44605888 | | | 2 | | | 1 | | | 3 | | | 6 | |
|  | | 10 | | 55158306 | | | 56577011 | | | 2 | | | 0 | | | 1 | | | 3 | |
|  | | 10 | | 79380592 | | | 80523665 | | | 20 | | | 0 | | | 7 | | | 27 | |
|  | | 19 | | 37142432 | | | 38142432 | | | 7 | | | 0 | | | 1 | | | 8 | |
|  | | 20 | | 59931850 | | | 60931850 | | | 0 | | | 0 | | | 0 | | | 0 | |
| **NH** | | 4 | | 68618549 | | | 70214717 | | | 5 | | | 0 | | | 2 | | | 7 | |
|  | | 4 | | 82694842 | | | 86046563 | | | 47 | | | 1 | | | 19 | | | 67 | |
|  | | 7 | | 92733594 | | | 94128623 | | | 2 | | | 0 | | | 5 | | | 7 | |
|  | | 9 | | 33413440 | | | 36308721 | | | 6 | | | 0 | | | 7 | | | 13 | |
|  | | 11 | | 18550799 | | | 19740093 | | | 51 | | | 1 | | | 3 | | | 55 | |
|  | | 14 | | 63236228 | | | 64334285 | | | 2 | | | 0 | | | 5 | | | 7 | |
|  | | 20 | | 40161395 | | | 41566022 | | | 14 | | | 0 | | | 11 | | | 25 | |
|  | | 20 | | 60332063 | | | 62075820 | | | 1 | | | 0 | | | 2 | | | 3 | |
|  | | 21 | | 20312917 | | | 22617426 | | | 4 | | | 0 | | | 19 | | | 23 | |
| **Table S7:** Final prioritization of GWA results based on a fixed sized region for the Welsh ponies (cont.) | | | | | | | | | | | | | | | | | | | | |
| **High Priority Regions Welsh Ponies Fixed Boundaries (cont.)** | | | | | | | | | | | | | | | | | | | | |
| **Trait** | | **Chr** | | **Min_Region** | | | **Max_Region** | | | **Protein_Coding** | | | **Pseudogenes** | | | **RNA_Genes** | | | **Total_Genes** | |
| **NH (cont.)** | | 24 | | 33296794 | | | 35972785 | | | 26 | | | 0 | | | 3 | | | 29 | |
| **GH** | | 1 | | 131703667 | | | 134211337 | | | 17 | | | 0 | | | 8 | | | 25 | |
|  | | 4 | | 68736860 | | | 70160028 | | | 5 | | | 0 | | | 1 | | | 6 | |
|  | | 4 | | 83544345 | | | 85997218 | | | 39 | | | 1 | | | 17 | | | 57 | |
|  | | 7 | | 92733594 | | | 94080126 | | | 2 | | | 0 | | | 4 | | | 6 | |
|  | | 11 | | 18327291 | | | 19740093 | | | 55 | | | 1 | | | 3 | | | 59 | |
|  | | 19 | | 30783482 | | | 31945588 | | | 4 | | | 0 | | | 0 | | | 4 | |
|  | | 20 | | 60435600 | | | 62288330 | | | 1 | | | 0 | | | 2 | | | 3 | |
|  | | 20 | | 64231849 | | | 65361251 | | | 3 | | | 0 | | | 3 | | | 6 | |
|  | | 22 | | 40533715 | | | 41565262 | | | 1 | | | 0 | | | 4 | | | 5 | |
| **LAM** | | 1 | | 48941032 | | | 49941032 | | | 6 | | | 0 | | | 16 | | | 22 | |
|  | | 2 | | 35623836 | | | 37133565 | | | 20 | | | 0 | | | 7 | | | 27 | |
|  | | 15 | | 50478261 | | | 51505138 | | | 2 | | | 0 | | | 1 | | | 3 | |
|  | | 19 | | 36772294 | | | 37828619 | | | 10 | | | 0 | | | 0 | | | 10 | |
|  | | 19 | | 59385237 | | | 62349890 | | | 17 | | | 1 | | | 6 | | | 24 | |
|  | | 20 | | 61518962 | | | 62585163 | | | 2 | | | 0 | | | 2 | | | 4 | |
|  | | 22 | | 3051367 | | | 4051367 | | | 5 | | | 0 | | | 5 | | | 10 | |
|  | | 28 | | 9961982 | | | 11166731 | | | 4 | | | 0 | | | 1 | | | 5 | |
| **Total** | |  | |  | | |  | | | **618** | | | **7** | | | **265** | | | **890** | |
|  |  | |  | | |  | | |  | | |  | | |  | | |  | | |
| **Medium Priority Regions Welsh Ponies Fixed Boundaries** | | | | | | | | | | | | | | | | | | | | |
| **Trait** | | **Chr** | | | **Min_Region** | | | **Max_Region** | | | **Protein_Coding** | | | **Pseudogenes** | | | **RNA_Genes** | | | **Total_Genes** |
| **Insulin** | | 6 | | | 82085066 | | | 84326234 | | | 11 | | | 0 | | | 15 | | | 26 |
|  | | 9 | | | 60013208 | | | 62548722 | | | 4 | | | 0 | | | 10 | | | 14 |
|  | | 15 | | | 53581224 | | | 55059632 | | | 7 | | | 0 | | | 12 | | | 19 |
|  | | 23 | | | 45584858 | | | 47452228 | | | 1 | | | 0 | | | 5 | | | 6 |
| **Insulin-OST** | | 9 | | | 51194853 | | | 52860209 | | | 4 | | | 0 | | | 3 | | | 7 |
| **Glucose** | | 8 | | | 88957249 | | | 90671577 | | | 2 | | | 0 | | | 3 | | | 5 |
|  | | 17 | | | 78520897 | | | 80497119 | | | 21 | | | 0 | | | 12 | | | 33 |
| **Table S7:** Final prioritization of GWA results based on a fixed sized region for the Welsh ponies (cont.) | | | | | | | | | | | | | | | | | | | | |
| **Medium Priority Regions Welsh Ponies Fixed Boundaries (cont.)** | | | | | | | | | | | | | | | | | | | | |
| **Trait** | | **Chr** | | | **Min_Region** | | | **Max_Region** | | | **Protein_Coding** | | | **Pseudogenes** | | | **RNA_Genes** | | | **Total_Genes** |
| **Glucose (cont.)** | | 29 | | | 22023122 | | | 23717070 | | | 16 | | | 0 | | | 6 | | | 22 |
| **Glucose-OST** | | 4 | | | 39650197 | | | 40852671 | | | 6 | | | 0 | | | 6 | | | 12 |
| **TG** | | 7 | | | 26033379 | | | 27135921 | | | 24 | | | 0 | | | 2 | | | 26 |
| **Adiponectin** | | 1 | | | 175282149 | | | 177572407 | | | 12 | | | 0 | | | 11 | | | 23 |
| **Leptin** | | 1 | | | 71870796 | | | 73660541 | | | 9 | | | 0 | | | 3 | | | 12 |
|  | | 6 | | | 1848093 | | | 2876386 | | | 2 | | | 0 | | | 1 | | | 3 |
| **ACTH** | | 1 | | | 43784734 | | | 45633993 | | | 2 | | | 0 | | | 3 | | | 5 |
|  | | 5 | | | 16369826 | | | 17849383 | | | 19 | | | 0 | | | 6 | | | 25 |
| **NH** | | 4 | | | 79198145 | | | 80890074 | | | 14 | | | 0 | | | 8 | | | 22 |
|  | | 15 | | | 72707370 | | | 73838081 | | | 7 | | | 0 | | | 6 | | | 13 |
|  | | 17 | | | 36298348 | | | 37356368 | | | 1 | | | 0 | | | 4 | | | 5 |
|  | | 21 | | | 6164894 | | | 7335706 | | | 4 | | | 0 | | | 7 | | | 11 |
| **GH** | | 4 | | | 73831018 | | | 76980368 | | | 13 | | | 0 | | | 12 | | | 25 |
|  | | 4 | | | 79307650 | | | 80890074 | | | 14 | | | 0 | | | 8 | | | 22 |
|  | | 11 | | | 14997051 | | | 16748131 | | | 34 | | | 0 | | | 6 | | | 40 |
|  | | 13 | | | 735274 | | | 1031380 | | | 6 | | | 0 | | | 2 | | | 8 |
|  | | 15 | | | 14562753 | | | 16156836 | | | 15 | | | 0 | | | 9 | | | 24 |
|  | | 16 | | | 26603995 | | | 28731661 | | | 14 | | | 0 | | | 3 | | | 17 |
|  | | 18 | | | 74558371 | | | 76575236 | | | 26 | | | 0 | | | 5 | | | 31 |
|  | | 18 | | | 79891110 | | | 81550756 | | | 13 | | | 0 | | | 12 | | | 25 |
|  | | 20 | | | 29641925 | | | 30949510 | | | 45 | | | 0 | | | 8 | | | 53 |
| **LAM** | | 2 | | | 29237934 | | | 30277141 | | | 30 | | | 0 | | | 3 | | | 33 |
|  | | 10 | | | 14874259 | | | 16488198 | | | 64 | | | 0 | | | 9 | | | 73 |
|  | | 13 | | | 24382636 | | | 26240597 | | | 29 | | | 0 | | | 3 | | | 32 |
|  | | 19 | | | 38625743 | | | 40126653 | | | 23 | | | 0 | | | 1 | | | 24 |
|  | | 30 | | | 12107858 | | | 13628439 | | | 7 | | | 0 | | | 8 | | | 15 |
|  | | 31 | | | 10111327 | | | 11009324 | | | 4 | | | 0 | | | 4 | | | 8 |
| **Total** | |  | | |  | | |  | | | **503** | | | **0** | | | **216** | | | **719** |
|  |  | |  | | |  | | |  | | |  | | |  | | |  | | |
| **Table S7:** Final prioritization of GWA results based on a fixed sized region for the Welsh ponies (cont.) | | | | | | | | | | | | | | | | | | | | |
| **Low Priority Regions Welsh Ponies Fixed Boundaries** | | | | | | | | | | | | | | | | | | | | |
| **Trait** | **Chr** | | **Min_Region** | | | **Max_Region** | | | **Protein_Coding** | | | **Pseudogenes** | | | **RNA_Genes** | | | **Total_Genes** | | |
| **Insulin** | 28 | | 38043945 | | | 39043945 | | | 18 | | | 0 | | | 5 | | | 23 | | |
| **Adiponectin** | 28 | | 40552952 | | | 41552952 | | | 14 | | | 0 | | | 4 | | | 18 | | |
| **Leptin** | 4 | | 47514169 | | | 48531048 | | | 3 | | | 0 | | | 4 | | | 7 | | |
|  | 14 | | 59795756 | | | 60795756 | | | 5 | | | 0 | | | 1 | | | 6 | | |
|  | 28 | | 40004716 | | | 41004716 | | | 17 | | | 0 | | | 6 | | | 23 | | |
| **NH** | 1 | | 43898249 | | | 44898249 | | | 1 | | | 0 | | | 1 | | | 2 | | |
|  | 6 | | 519810 | | | 1533178 | | | 6 | | | 0 | | | 2 | | | 8 | | |
|  | 10 | | 10327320 | | | 11327320 | | | 42 | | | 2 | | | 3 | | | 47 | | |
|  | 20 | | 29660893 | | | 30660893 | | | 36 | | | 0 | | | 6 | | | 42 | | |
|  | 21 | | 23490259 | | | 25495726 | | | 16 | | | 0 | | | 3 | | | 19 | | |
| **GH** | 4 | | 46293329 | | | 48351529 | | | 3 | | | 0 | | | 7 | | | 10 | | |
|  | 6 | | 519810 | | | 1654034 | | | 6 | | | 0 | | | 3 | | | 9 | | |
|  | 10 | | 10729405 | | | 11729405 | | | 34 | | | 0 | | | 3 | | | 37 | | |
|  | 21 | | 21039433 | | | 22042349 | | | 3 | | | 0 | | | 6 | | | 9 | | |
|  | 21 | | 23276930 | | | 24491948 | | | 12 | | | 0 | | | 2 | | | 14 | | |
| **LAM** | 14 | | 58430834 | | | 60167233 | | | 9 | | | 0 | | | 6 | | | 15 | | |
| **Total** |  | |  | | |  | | | **225** | | | **2** | | | **62** | | | **289** | | |

Regions were categorized as high priority (regions found on metanalysis or was shared with another trait and considered an ROI), medium priority (region was identified as an ROI in at least one GWA), or low priority (region was shared with across traits but region was not an ROI). Final region boundaries of the region were defined as 500Kb 5’ of the lowest SNP (Min_ROI) and 500Kb 3’ of the highest (Max_ROI) SNP across relevant GWA data. The total number of genes includes all protein-coding genes, pseudogenes, and RNA genes identified for region based on EquCab3. Shared regions across prioritized traits are indicated by highlighted chromosomes.

| **Table S8:** Prioritization of the GWA results of Morgan horses for a fixed-size region | | | | | | | | | |
| --- | --- | --- | --- | --- | --- | --- | --- | --- | --- |
| **Trait** | **Chr** | **Summary** | **Min_SNP** | **Max_SNP** | **Sugg_SNPs** | **Sign_SNPs** | **Min_Region** | **Max_Region** | **Total_Genes** |
| **Insulin** | 2 |  | 117366086 | 117410894 | 5 | 1 | 116866086 | 117910894 | 9 |
|  | 3 |  | 115316619 | 115326166 | 4 | 0 | 114816619 | 115826166 | 12 |
|  | 4 |  | 97370223 | NA | 1 | 0 | 96870223 | 97870223 | 30 |
|  | 5 |  | 88722709 | NA | 2 | 0 | 88222709 | 89222709 | 6 |
|  | 8 |  | 36946690 | NA | 1 | 0 | 36446690 | 37446690 | 13 |
|  | 8 |  | 62414695 | 62422169 | 3 | 0 | 61914695 | 62922169 | 5 |
|  | 10 |  | 54997568 | 55022644 | 3 | 0 | 54497568 | 55522644 | 1 |
|  | 18 |  | 38197723 | NA | 1 | 0 | 37697723 | 38697723 | 7 |
|  | 19 |  | 20841248 | NA | 1 | 0 | 20341248 | 21341248 | 17 |
|  | 20 | **F** | 4635861 | 4702640 | 7 | 0 | 4135861 | 5202640 | 16 |
|  | 24 | **F H** | 21134897 | NA | 1 | 0 | 20634897 | 21634897 | 14 |
|  | 26 |  | 39653507 | NA | 1 | 0 | 39153507 | 40153507 | 22 |
| **Insulin-OST** | 2 |  | 22468309 | 22541921 | 4 | 1 | 21968309 | 23041921 | 16 |
|  | 2 |  | 51548258 | 51661415 | 7 | 0 | 51048258 | 52161415 | 33 |
|  | 4 | **E** | 28373202 | NA | 1 | 0 | 27873202 | 28873202 | 2 |
|  | 4 |  | 57780431 | 57786154 | 2 | 0 | 57280431 | 58286154 | 15 |
|  | 6 |  | 32931767 | 33694226 | 2 | 0 | 32431767 | 34194226 | 30 |
|  | 8 |  | 10116471 | NA | 1 | 0 | 9616471 | 10616471 | 17 |
|  | 10 | **B H** | 71996093 | 73613162 | 50 | 5 | 71496093 | 74113162 | 17 |
|  | 11 | **F** | 18848207 | 19009809 | 7 | 0 | 18348207 | 19509809 | 47 |
|  | 20 |  | 51914168 | NA | 1 | 0 | 51414168 | 52414168 | 22 |
|  | 21 |  | 20781491 | NA | 1 | 0 | 20281491 | 21281491 | 12 |
| **Glucose** | 4 | **E H** | 17981325 | 18477651 | 33 | 11 | 17481325 | 18977651 | 9 |
|  | 8 | **H** | 11530408 | 12159746 | 5 | 1 | 11030408 | 12659746 | 18 |
|  | 16 |  | 42711571 | NA | 1 | 0 | 42211571 | 43211571 | 15 |
|  | 28 |  | 36615983 | NA | 1 | 0 | 36115983 | 37115983 | 34 |
|  | 29 |  | 9494870 | NA | 1 | 0 | 8994870 | 9994870 | 14 |
|  | 31 |  | 21504871 | NA | 1 | 0 | 21004871 | 22004871 | 9 |
| **Glucose-OST** | 2 |  | 62607747 | NA | 1 | 0 | 62107747 | 63107747 | 11 |
|  | 3 | **H** | 56674808 | 58220254 | 85 | 53 | 56174808 | 58720254 | 19 |
| **Table S8:** Prioritization of the GWA results of Morgan horses for a fixed-size region (cont.) | | | | | | | | | |
| **Trait** | **Chr** | **Summary** | **Min_SNP** | **Max_SNP** | **Sugg_SNPs** | **Sign_SNPs** | **Min_Region** | **Max_Region** | **Total_Genes** |
| **Glucose-OST (cont.)** | 4 | **E H** | 27505119 | 28710128 | 39 | 4 | 27005119 | 29210128 | 8 |
|  | 14 |  | 28998387 | 29000329 | 2 | 0 | 28498387 | 29500329 | 24 |
|  | 25 |  | 18872032 | NA | 1 | 0 | 18372032 | 19372032 | 23 |
|  | 26 |  | 22407530 | 23379414 | 23 | 2 | 21907530 | 23879414 | 6 |
| **NEFA** | 1 |  | 166669064 | 166888483 | 3 | 0 | 166169064 | 167388483 | 8 |
|  | 1 | **H** | 185892360 | 186617146 | 25 | 15 | 185392360 | 187117146 | 37 |
|  | 2 |  | 106012533 | 106052266 | 6 | 1 | 105512533 | 106552266 | 16 |
|  | 7 |  | 86986401 | 87004808 | 3 | 0 | 86486401 | 87504808 | 4 |
|  | 9 |  | 76549280 | 76571642 | 3 | 0 | 76049280 | 77071642 | 13 |
|  | 15 |  | 66056425 | NA | 1 | 0 | 65556425 | 66556425 | 12 |
|  | 17 | **H** | 13427110 | 14189583 | 14 | 1 | 12927110 | 14689583 | 6 |
|  | 18 |  | 7685942 | 9565563 | 44 | 0 | 7185942 | 10065563 | 18 |
|  | 19 |  | 48235446 | NA | 1 | 0 | 47735446 | 48735446 | 21 |
|  | 24 | **F H** | 20381260 | 20888104 | 2 | 1 | 19881260 | 21388104 | 32 |
|  | 24 |  | 45325106 | 45675218 | 5 | 0 | 44825106 | 46175218 | 28 |
|  | 30 |  | 6239856 | 6258423 | 5 | 0 | 5739856 | 6758423 | 9 |
|  | 30 | **H** | 20974703 | 21044590 | 11 | 4 | 20474703 | 21544590 | 3 |
| **TG** | 1 |  | 126407798 | 127401777 | 6 | 0 | 125907798 | 127901777 | 32 |
|  | 10 |  | 65383517 | NA | 1 | 0 | 64883517 | 65883517 | 6 |
|  | 20 |  | 52368013 | 52589211 | 4 | 1 | 51868013 | 53089211 | 9 |
|  | 21 |  | 49201984 | 49202284 | 2 | 0 | 48701984 | 49702284 | 2 |
| **Adiponectin** | 1 |  | 129650721 | 129653375 | 2 | 0 | 129150721 | 130153375 | 18 |
|  | 1 |  | 138037003 | NA | 1 | 0 | 137537003 | 138537003 | 8 |
|  | 2 | **H** | 16747148 | 17739125 | 38 | 27 | 16247148 | 18239125 | 50 |
|  | 4 | **H** | 36557672 | 38544490 | 54 | 4 | 36057672 | 39044490 | 35 |
|  | 6 | **H** | 32601529 | 32727370 | 19 | 1 | 32101529 | 33227370 | 15 |
|  | 6 | **H** | 67997807 | 69847785 | 68 | 6 | 67497807 | 70347785 | 82 |
|  | 7 |  | 21524454 | 21986901 | 14 | 0 | 21024454 | 22486901 | 26 |
|  | 7 |  | 32963159 | 32963459 | 2 | 0 | 32463159 | 33463459 | 37 |
|  | 8 |  | 3347264 | 3419299 | 6 | 0 | 2847264 | 3919299 | 26 |
| **Table S8:** Prioritization of the GWA results of Morgan horses for a fixed-size region (cont.) | | | | | | | | | |
| **Trait** | **Chr** | **Summary** | **Min_SNP** | **Max_SNP** | **Sugg_SNPs** | **Sign_SNPs** | **Min_Region** | **Max_Region** | **Total_Genes** |
| **Adiponectin (cont.)** | 15 |  | 21830373 | 21834175 | 2 | 0 | 21330373 | 22334175 | 4 |
|  | 15 |  | 66865469 | 66893151 | 4 | 0 | 66365469 | 67393151 | 8 |
|  | 18 | **F H** | 41448414 | NA | 1 | 1 | 40948414 | 41948414 | 10 |
|  | 18 |  | 49705278 | 49893633 | 7 | 0 | 49205278 | 50393633 | 28 |
|  | 19 |  | 25833383 | 25859655 | 2 | 0 | 25333383 | 26359655 | 14 |
|  | 20 | **F H** | 3734902 | 3954772 | 12 | 0 | 3234902 | 4454772 | 16 |
|  | 20 |  | 1882774 | NA | 1 | 0 | 1382774 | 2382774 | 11 |
|  | 21 |  | 49478363 | NA | 1 | 0 | 48978363 | 49978363 | 1 |
| **Leptin** | 1 |  | 130957068 | 131062691 | 3 | 0 | 130457068 | 131562691 | 11 |
|  | 4 | **E** | 52373692 | 52614368 | 22 | 0 | 51873692 | 53114368 | 14 |
|  | 6 |  | 38446793 | NA | 1 | 0 | 37946793 | 38946793 | 21 |
|  | 8 |  | 8682147 | NA | 1 | 0 | 8182147 | 9182147 | 37 |
|  | 19 | **H** | 51360775 | 53132722 | 57 | 27 | 50860775 | 53632722 | 26 |
|  | 21 |  | 16547954 | 16608200 | 3 | 0 | 16047954 | 17108200 | 8 |
|  | 24 | **H** | 27275709 | 29038412 | 65 | 14 | 26775709 | 29538412 | 10 |
|  | 25 |  | 27438558 | 27907420 | 14 | 2 | 26938558 | 28407420 | 31 |
| **ACTH** | 1 | **E H** | 83546191 | 83734040 | 17 | 4 | 83046191 | 84234040 | 23 |
|  | 3 | **D H** | 43335201 | 44116411 | 13 | 0 | 42835201 | 44616411 | 10 |
|  | 3 | **H** | 103056163 | 103438726 | 49 | 34 | 102556163 | 103938726 | 8 |
|  | 5 | **H** | 25785666 | 27061038 | 32 | 10 | 25285666 | 27561038 | 26 |
|  | 10 |  | 67992633 | 67997136 | 2 | 0 | 67492633 | 68497136 | 12 |
|  | 10 |  | 70528773 | NA | 1 | 0 | 70028773 | 71028773 | 12 |
|  | 11 | **F** | 18728679 | 18904099 | 4 | 0 | 18228679 | 19404099 | 44 |
|  | 11 |  | 52897545 | 53669056 | 32 | 0 | 52397545 | 54169056 | 24 |
|  | 13 |  | 25806289 | NA | 1 | 0 | 25306289 | 26306289 | 15 |
|  | 16 |  | 31200001 | NA | 1 | 0 | 30700001 | 31700001 | 13 |
|  | 18 | **F** | 41392781 | NA | 1 | 0 | 40892781 | 41892781 | 10 |
|  | 20 |  | 29056288 | NA | 1 | 0 | 28556288 | 29556288 | 45 |
|  | 21 |  | 11112604 | NA | 1 | 0 | 10612604 | 11612604 | 11 |
|  | 21 |  | 24436227 | 24439739 | 3 | 0 | 23936227 | 24939739 | 12 |
| **Table S8:** Prioritization of the GWA results of Morgan horses for a fixed-size region (cont.) | | | | | | | | | |
| **Trait** | **Chr** | **Summary** | **Min_SNP** | **Max_SNP** | **Sugg_SNPs** | **Sign_SNPs** | **Min_Region** | **Max_Region** | **Total_Genes** |
| **ACTH (cont.)** | 25 |  | 13299542 | NA | 1 | 0 | 12799542 | 13799542 | 15 |
|  | 25 |  | 14989527 | NA | 1 | 0 | 14489527 | 15489527 | 13 |
|  | 31 |  | 16965044 | 17737242 | 4 | 0 | 16465044 | 18237242 | 30 |
| **NH** | 1 | **F** | 78493587 | 79782621 | 37 | 0 | 77993587 | 80282621 | 20 |
|  | 1 | **E** | 82958480 | 83232130 | 10 | 0 | 82458480 | 83732130 | 15 |
|  | 2 |  | 93824111 | 93833011 | 2 | 0 | 93324111 | 94333011 | 3 |
|  | 4 | **E H** | 52076906 | 53659651 | 149 | 110 | 51576906 | 54159651 | 22 |
|  | 5 |  | 59796357 | 60233277 | 10 | 0 | 59296357 | 60733277 | 7 |
|  | 5 |  | 65804297 | 65824216 | 3 | 0 | 65304297 | 66324216 | 3 |
|  | 6 | **H** | 64502443 | 65350057 | 44 | 12 | 64002443 | 65850057 | 17 |
|  | 8 |  | 29756282 | NA | 1 | 0 | 29256282 | 30256282 | 7 |
|  | 9 |  | 49062306 | 49078134 | 2 | 0 | 48562306 | 49578134 | 8 |
|  | 14 |  | 74532493 | NA | 1 | 0 | 74032493 | 75032493 | 9 |
|  | 18 |  | 2306238 | NA | 1 | 0 | 1806238 | 2806238 | 7 |
|  | 19 | **H** | 1188889 | 1197320 | 3 | 2 | 688889 | 1697320 | 10 |
|  | 19 | **H** | 34421059 | 36247260 | 102 | 23 | 33921059 | 36747260 | 48 |
|  | 19 |  | 46479290 | 47156982 | 5 | 1 | 45979290 | 47656982 | 25 |
|  | 21 |  | 4745903 | NA | 1 | 0 | 4245903 | 5245903 | 31 |
|  | 24 |  | 42026470 | 42450741 | 9 | 1 | 41526470 | 42950741 | 23 |
| **GH** | 1 | **F** | 79175507 | 79234421 | 5 | 0 | 78675507 | 79734421 | 9 |
|  | 1 |  | 109778420 | 109819993 | 4 | 0 | 109278420 | 110319993 | 13 |
|  | 1 | **H** | 122383349 | 123036781 | 71 | 31 | 121883349 | 123536781 | 20 |
|  | 2 |  | 85183513 | 86093522 | 21 | 10 | 84683513 | 86593522 | 14 |
|  | 4 |  | 3032922 | NA | 1 | 1 | 2532922 | 3532922 | 7 |
|  | 6 | **F** | 3139850 | 3453652 | 2 | 0 | 2639850 | 3953652 | 10 |
|  | 6 |  | 6272129 | 6335115 | 8 | 0 | 5772129 | 6835115 | 27 |
|  | 6 |  | 15729023 | 16202020 | 8 | 0 | 15229023 | 16702020 | 16 |
|  | 7 |  | 26684853 | 26701040 | 6 | 0 | 26184853 | 27201040 | 28 |
|  | 8 |  | 63557829 | NA | 1 | 0 | 63057829 | 64057829 | 9 |
|  | 17 | **H** | 32020513 | 33031579 | 39 | 2 | 31520513 | 33531579 | 6 |
| **Table S8:** Prioritization of the GWA results of Morgan horses for a fixed-size region (cont.) | | | | | | | | | |
| **Trait** | **Chr** | **Summary** | **Min_SNP** | **Max_SNP** | **Sugg_SNPs** | **Sign_SNPs** | **Min_Region** | **Max_Region** | **Total_Genes** |
| **GH (cont.)** | 18 |  | 2423391 | NA | 1 | 0 | 1923391 | 2923391 | 9 |
|  | 22 |  | 45719751 | 48733979 | 2 | 0 | 45219751 | 49233979 | 70 |
|  | 29 |  | 19108245 | 19432974 | 7 | 2 | 18608245 | 19932974 | 15 |
| **LAM** | 2 |  | 66192812 | NA | 1 | 1 | 65692812 | 66692812 | 11 |
|  | 3 |  | 3294278 | NA | 1 | 0 | 2794278 | 3794278 | 10 |
|  | 4 | **E H** | 17509325 | 19295909 | 52 | 4 | 17009325 | 19795909 | 18 |
|  | 6 | **F** | 3466933 | NA | 1 | 0 | 2966933 | 3966933 | 8 |
|  | 6 |  | 79661858 | NA | 1 | 0 | 79161858 | 80161858 | 4 |
|  | 8 |  | 59199626 | 60121756 | 24 | 0 | 58699626 | 60621756 | 21 |
|  | 12 | **H** | 33127411 | 34414133 | 53 | 27 | 32627411 | 34914133 | 54 |
|  | 14 |  | 66311023 | 66688404 | 15 | 0 | 65811023 | 67188404 | 7 |
|  | 14 | **H** | 88975206 | 90135630 | 48 | 9 | 88475206 | 90635630 | 37 |
|  | 16 |  | 64556111 | NA | 1 | 0 | 64056111 | 65056111 | 8 |
|  | 16 |  | 74667638 | NA | 1 | 0 | 74167638 | 75167638 | 21 |
|  | 18 | **H** | 31710749 | 33317633 | 65 | 33 | 31210749 | 33817633 | 18 |
|  | 19 | **H** | 30133826 | NA | 51 | 3 | 29633826 | 30633826 | 6 |
|  | 22 | **B H** | 3616445 | 4853827 | 75 | 45 | 3116445 | 5353827 | 21 |
|  | 22 |  | 13852015 | NA | 1 | 0 | 13352015 | 14352015 | 11 |
|  | 22 |  | 23806850 | NA | 1 | 0 | 23306850 | 24306850 | 25 |
|  | 23 | **H** | 11116499 | 12515439 | 51 | 46 | 10616499 | 13015439 | 19 |
|  | 31 |  | 6804894 | NA | 1 | 0 | 6304894 | 7304894 | 3 |

To be considered an ROI, at least five SNP had to exceed the suggestive threshold (1.0e-05) with one SNP exceeding the threshold for genome-wide significance (5.98e-08). Provided in the table is the base pair position of the lowest (Min_SNP) and highest (Max_SNP) SNP in the region, the min (Min_ROI) and maximum (Max_ROI) boundaries of the region based on a fixed value of 500Kb 5’ of the Min_SNP and 500Kb 3’ of the Max_SNP, as well as the number of SNPs per region which exceeded the suggestive (Sugg_SNPs) and genome-wide significance (Sign_SNPs) threshold. The total number of genes includes all protein-coding genes, pseudogenes, and RNA genes identified for region based on EquCab3. A black box in the summary column indicates the region did not meet the criteria to be considered an ROI and was not shared with one or more Welsh pony cohorts or trait. Letters in the summary column represent: (B) region was shared with one or more Welsh pony GWA cohorts and at least one region was considered an ROI, (D) region was shared with one or more Welsh pony GWA cohort but no regions met the criteria for an ROI, (E) region was shared with another trait in the Morgan GWA and at least one region was considered an ROI, (F) region was shared with another trait in the Morgan GWA but no regions met the criteria for an ROI, (H) region was identified as shared across breeds on metanalysis and was considered a MA-ROI. Highlighted chromosomes (Chr) indicate regions which were shared with several traits.

| **Table S9:** Final prioritization of the GWA results based on a fixed-size region for the Morgan horses | | | | | | | | | | | | | | | |
| --- | --- | --- | --- | --- | --- | --- | --- | --- | --- | --- | --- | --- | --- | --- | --- |
| **High Priority Regions Morgan Horses Fixed Boundaries** | | | | | | | | | | | | | | | |
| **Trait** | | **Chr** | | **Min_Region** | | **Max_Region** | | **Protein_Coding** | | **Pseudogenes** | | **RNA_Genes** | | **Total_Genes** | |
| **Insulin** | | 24 | | 20634897 | | 21634897 | | 11 | | 0 | | 3 | | 14 | |
| **Insulin-OST** | | 4 | | 27873202 | | 28873202 | | 2 | | 0 | | 0 | | 2 | |
|  | | 10 | | 71496093 | | 74113162 | | 11 | | 1 | | 5 | | 17 | |
| **Glucose** | | 4 | | 17481325 | | 18977651 | | 6 | | 0 | | 3 | | 9 | |
|  | | 8 | | 11030408 | | 12659746 | | 9 | | 0 | | 9 | | 18 | |
| **Glucose-OST** | | 3 | | 56174808 | | 58720254 | | 12 | | 0 | | 7 | | 19 | |
|  | | 4 | | 27005119 | | 29210128 | | 5 | | 0 | | 3 | | 8 | |
| **NEFA** | | 1 | | 185392360 | | 187117146 | | 24 | | 0 | | 13 | | 37 | |
|  | | 17 | | 12927110 | | 14689583 | | 4 | | 0 | | 2 | | 6 | |
|  | | 24 | | 19881260 | | 21388104 | | 28 | | 0 | | 4 | | 32 | |
|  | | 30 | | 20474703 | | 21544590 | | 1 | | 0 | | 2 | | 3 | |
| **Adiponectin** | | 2 | | 16247148 | | 18239125 | | 26 | | 1 | | 23 | | 50 | |
|  | | 4 | | 36057672 | | 39044490 | | 28 | | 0 | | 7 | | 35 | |
|  | | 6 | | 32101529 | | 33227370 | | 9 | | 0 | | 6 | | 15 | |
|  | | 6 | | 67497807 | | 70347785 | | 77 | | 0 | | 5 | | 82 | |
|  | | 18 | | 40948414 | | 41948414 | | 4 | | 0 | | 6 | | 10 | |
|  | | 20 | | 3234902 | | 4454772 | | 12 | | 0 | | 4 | | 16 | |
| **Leptin** | | 4 | | 51873692 | | 53114368 | | 6 | | 0 | | 8 | | 14 | |
|  | | 19 | | 50860775 | | 53632722 | | 13 | | 0 | | 13 | | 26 | |
|  | | 24 | | 26775709 | | 29538412 | | 0 | | 0 | | 10 | | 10 | |
| **ACTH** | | 1 | | 83046191 | | 84234040 | | 17 | | 1 | | 5 | | 23 | |
|  | | 3 | | 42835201 | | 44616411 | | 2 | | 1 | | 7 | | 10 | |
|  | | 3 | | 102556163 | | 103938726 | | 3 | | 0 | | 5 | | 8 | |
|  | | 5 | | 25285666 | | 27561038 | | 12 | | 0 | | 14 | | 26 | |
| **NH** | | 1 | | 82458480 | | 83732130 | | 10 | | 0 | | 5 | | 15 | |
|  | | 4 | | 51576906 | | 54159651 | | 10 | | 0 | | 12 | | 22 | |
|  | | 6 | | 64002443 | | 65850057 | | 9 | | 0 | | 8 | | 17 | |
|  | | 19 | | 688889 | | 1697320 | | 5 | | 1 | | 4 | | 10 | |
|  | | 19 | | 33921059 | | 36747260 | | 40 | | 0 | | 8 | | 48 | |
| **Table S9:** Final prioritization of the GWA results based on a fixed-size region for the Morgan horses (cont.) | | | | | | | | | | | | | | | |
| **High Priority Regions Morgan Horses Fixed Boundaries (cont.)** | | | | | | | | | | | | | | | |
| **Trait** | | **Chr** | | **Min_Region** | | **Max_Region** | | **Protein_Coding** | | **Pseudogenes** | | **RNA_Genes** | | **Total_Genes** | |
| **GH** | | 1 | | 121883349 | | 123536781 | | 19 | | 0 | | 1 | | 20 | |
|  | | 17 | | 31520513 | | 33531579 | | 2 | | 1 | | 3 | | 6 | |
| **LAM** | | 4 | | 17009325 | | 19795909 | | 8 | | 1 | | 9 | | 18 | |
|  | | 12 | | 32627411 | | 34914133 | | 31 | | 0 | | 23 | | 54 | |
|  | | 14 | | 88475206 | | 90635630 | | 23 | | 0 | | 14 | | 37 | |
|  | | 18 | | 31210749 | | 33817633 | | 11 | | 0 | | 7 | | 18 | |
|  | | 19 | | 29633826 | | 30633826 | | 6 | | 0 | | 0 | | 6 | |
|  | | 22 | | 3116445 | | 5353827 | | 12 | | 0 | | 9 | | 21 | |
|  | | 23 | | 10616499 | | 13015439 | | 4 | | 0 | | 15 | | 19 | |
| **Total** | |  | |  | |  | | **512** | | **7** | | **282** | | **801** | |
|  |  | |  | |  | |  | |  | |  | |  | |  |
| **Medium Priority Regions Morgan Horses Fixed Boundaries** | | | | | | | | | | | | | | | |
| **Trait** | | **Chr** | | **Min_Region** | | **Max_Region** | | **Protein_Coding** | | **Pseudogenes** | | **RNA_Genes** | | **Total_Genes** | |
| **Insulin** | | 2 | | 116866086 | | 117910894 | | 6 | | 0 | | 3 | | 9 | |
| **Glucose-OST** | | 26 | | 21907530 | | 23879414 | | 0 | | 0 | | 6 | | 6 | |
| **NEFA** | | 2 | | 105512533 | | 106552266 | | 13 | | 0 | | 3 | | 16 | |
| **Leptin** | | 25 | | 26938558 | | 28407420 | | 29 | | 1 | | 1 | | 31 | |
| **NH** | | 19 | | 45979290 | | 47656982 | | 22 | | 0 | | 3 | | 25 | |
|  | | 24 | | 41526470 | | 42950741 | | 15 | | 0 | | 8 | | 23 | |
| **GH** | | 2 | | 84683513 | | 86593522 | | 8 | | 0 | | 6 | | 14 | |
|  | | 29 | | 18608245 | | 19932974 | | 13 | | 0 | | 2 | | 15 | |
| **Total** | |  | |  | |  | | **106** | | **1** | | **32** | | **139** | |
|  |  | |  | |  | |  | |  | |  | |  | |  |
| **Low Priority Regions Morgan Horses Fixed Boundaries** | | | | | | | | | | | | | | | |
| **Trait** | | **Chr** | | **Min_Region** | | **Max_Region** | | **Protein_Coding** | | **Pseudogenes** | | **RNA_Genes** | | **Total_Genes** | |
| **Insulin** | | 20 | | 4135861 | | 5202640 | | 9 | | 0 | | 7 | | 16 | |
| **Insulin-OST** | | 11 | | 18348207 | | 19509809 | | 44 | | 1 | | 2 | | 47 | |
| **ACTH** | | 11 | | 18228679 | | 19404099 | | 42 | | 1 | | 1 | | 44 | |
|  | | 18 | | 40892781 | | 41892781 | | 4 | | 0 | | 6 | | 10 | |
| **Table S9:** Final prioritization of the GWA results based on a fixed-size region for the Morgan horses (cont.) | | | | | | | | | | | | | | | |
| **Low Priority Regions Morgan Horses Fixed Boundaries (cont.)** | | | | | | | | | | | | | | | |
| **Trait** | | **Chr** | | **Min_Region** | | **Max_Region** | | **Protein_Coding** | | **Pseudogenes** | | **RNA_Genes** | | **Total_Genes** | |
| **NH** | | 1 | | 77993587 | | 80282621 | | 8 | | 0 | | 12 | | 20 | |
| **GH** | | 1 | | 78675507 | | 79734421 | | 5 | | 0 | | 4 | | 9 | |
|  | | 6 | | 2639850 | | 3953652 | | 4 | | 0 | | 6 | | 10 | |
| **LAM** | | 6 | | 2966933 | | 3966933 | | 4 | | 0 | | 4 | | 8 | |
| **Total** | |  | |  | |  | | **120** | | **2** | | **42** | | **164** | |

Regions were categorized as high priority (regions found on metanalysis or was shared with another trait and considered an ROI), medium priority (region was identified as an ROI in at least one GWA), or low priority (region was shared with across traits but region was not an ROI). Final region boundaries of the region were defined as 500Kb 5’ of the lowest SNP (Min_ROI) and 500Kb 3’ of the highest (Max_ROI) SNP across relevant GWA data. The total number of genes includes all protein-coding genes, pseudogenes, and RNA genes identified for region based on EquCab3. Shared regions across prioritized traits are indicated by highlighted chromosomes.

| **Table S10:** Prioritization of the GWA results of the full Welsh pony cohort based on LD-bound regions | | | | | | | | | |
| --- | --- | --- | --- | --- | --- | --- | --- | --- | --- |
| **Trait** | **Chr** | **Summary** | **Min_SNP** | **Max_SN** | **Sugg_SNPs** | **Sign_SNPs** | **Min_Region** | **Max_Region** | **Total_Genes** |
| **Insulin** | 1 |  | 46119989 | NA | 1 | 0 | 46069989 | 46169989 | 0 |
|  | 5 | **E H** | 40632818 | 41895313 | 86 | 4 | 35409104 | 44806458 | 306 |
|  | 6 | **A** | 82238815 | 82729921 | 11 | 0 | 81685572 | 83066256 | 17 |
|  | 8 | **A** | 75410291 | 75771110 | 6 | 0 | 74768024 | 76080554 | 13 |
|  | 9 |  | 83981022 | 84014912 | 6 | 0 | 83924785 | 84136547 | 11 |
|  | 13 |  | 14234078 | 14849603 | 7 | 0 | 12830836 | 16025943 | 19 |
|  | 13 |  | 37700109 | 37723843 | 3 | 1 | 37642362 | 37732522 | 0 |
|  | 14 |  | 31226680 | 31583686 | 4 | 0 | 31004830 | 31595632 | 5 |
|  | 15 | **A H** | 5748638 | 6140956 | 28 | 5 | 5748377 | 6612684 | 1 |
|  | 15 |  | 54081224 | 54559632 | 5 | 1 | 54076168 | 54634446 | 5 |
|  | 19 |  | 9606463 | 9637331 | 4 | 3 | 9604680 | 9680011 | 0 |
|  | 23 |  | 46084858 | 46952228 | 6 | 3 | 45940500 | 46233500 | 2 |
|  | 24 | **H** | 28580621 | 29056428 | 15 | 7 | 28451012 | 29887250 | 6 |
|  | 34 |  | 38174280 | NA | 1 | 0 | 38124280 | 38224280 | 0 |
|  | 28 |  | 38543945 | NA | 1 | 0 | 38493945 | 38593945 | 4 |
| **Insulin-OST** | 1 |  | 119102659 | 119140428 | 6 | 0 | 117422338 | 119310838 | 17 |
|  | 8 | **C** | 73418239 | 73458142 | 4 | 0 | 73223448 | 73648399 | 2 |
|  | 9 |  | 27879884 | NA | 1 | 0 | 27829884 | 27929884 | 1 |
|  | 9 |  | 28468074 | NA | 1 | 0 | 28418074 | 28518074 | 3 |
|  | 20 |  | 59181583 | 59182258 | 2 | 0 | 59165490 | 59819879 | 5 |
|  | 28 | **H** | 39385975 | 39462810 | 4 | 2 | 39322188 | 39488807 | 9 |
|  | 31 |  | 8856537 | 8855069 | 2 | 0 | 8487501 | 9386141 | 16 |
| **Glucose** | 3 |  | 32093888 | 32098312 | 4 | 0 | 32038651 | 32100834 | 2 |
|  | 8 | **C** | 84913969 | 85008392 | 2 | 2 | 84907297 | 85013865 | 0 |
|  | 15 | **H** | 83778178 | NA | 1 | 1 | 83728178 | 83828178 | 2 |
|  | 16 |  | 86563618 | 86743699 | 2 | 0 | 86419997 | 87063084 | 3 |
|  | 22 |  | 42270349 | 42320092 | 2 | 0 | 42222066 | 42357143 | 2 |
|  | 28 |  | 14969841 | 15045427 | 2 | 1 | 14968044 | 15079576 | 0 |
|  | 28 |  | 33576312 | NA | 1 | 0 | 33526312 | 33626312 | 1 |
| **Table S10:** Prioritization of the GWA results of the full Welsh pony cohort based on LD-bound regions (cont.) | | | | | | | | | |
| **Trait** | **Chr** | **Summary** | **Min_SNP** | **Max_SN** | **Sugg_SNPs** | **Sign_SNPs** | **Min_Region** | **Max_Region** | **Total_Genes** |
| **Glucose (cont.)** | 29 | **A** | 22555245 | 22557916 | 2 | 0 | 22420947 | 22764383 | 9 |
| **Glucose-OST** | 1 |  | 185361795 | NA | 1 | 0 | 185311795 | 185411795 | 2 |
|  | 4 |  | 8502301 | 8522723 | 4 | 0 | 8380352 | 8750255 | 5 |
|  | 4 |  | 40150197 | 40352671 | 11 | 4 | 40143954 | 40782593 | 9 |
|  | 7 |  | 76975314 | NA | 1 | 0 | 76925314 | 77025314 | 5 |
|  | 23 | **C** | 10182647 | 10226427 | 4 | 2 | 9741040 | 10226824 | 2 |
| **NEFA** | 4 |  | 14831152 | 14840371 | 4 | 0 | 14262252 | 15798579 | 40 |
|  | 6 |  | 68206430 | 68512033 | 2 | 0 | 67662851 | 69169794 | 39 |
|  | 6 | **C** | 77102911 | NA | 1 | 0 | 77052911 | 77152911 | 1 |
|  | 8 |  | 11128642 | 11158885 | 2 | 0 | 10809267 | 11158996 | 5 |
|  | 8 |  | 69737476 | NA | 1 | 0 | 69687476 | 69787476 | 1 |
|  | 19 | **H** | 1055718 | NA | 1 | 1 | 1005718 | 1105718 | 2 |
|  | 20 |  | 8830210 | NA | 1 | 0 | 8780210 | 8880210 | 1 |
|  | 20 |  | 26078001 | NA | 1 | 0 | 26028001 | 26128001 | 15 |
|  | 22 | **C** | 19009107 | NA | 1 | 0 | 18959107 | 19059107 | 1 |
|  | 31 |  | 13902942 | NA | 1 | 0 | 13852942 | 13952942 | 0 |
| **TG** | 1 |  | 153409995 | 153700953 | 2 | 0 | 152034119 | 154351987 | 11 |
|  | 4 |  | 93870436 | NA | 1 | 0 | 93820436 | 93920436 | 3 |
|  | 12 | **C** | 35927778 | 35956541 | 4 | 1 | 35859835 | 36313678 | 13 |
| **Adiponectin** | 17 |  | 61546409 | 61552964 | 3 | NA | 61267646 | 61749127 | 7 |
|  | 18 | **H** | 60290699 | 60393507 | 10 | 5 | 60060215 | 61349045 | 13 |
| **Leptin** | 1 |  | 72370796 | 73160541 | 31 | 1 | 71902092 | 78569116 | 57 |
|  | 5 | **E** | 43015591 | 43412260 | 24 | NA | 39751797 | 50431769 | 239 |
|  | 7 | **A H** | 67955613 | 67964668 | 5 | 4 | 67910114 | 68117086 | 1 |
|  | 10 | **C H** | 866333 | 884264 | 3 | 1 | 692055 | 956048 | 4 |
|  | 21 | **E H** | NA | NA | 2 | 1 | 22940681 | 23516697 | 1 |
|  | 26 |  | 11291558 | NA | 1 | NA | 11241558 | 11341558 | 1 |
|  | 28 |  | 36456338 | 36459615 | 3 | NA | 36070224 | 36467537 | 15 |
| **ACTH** | 1 | **H** | 70266479 | 70832972 | 20 | 1 | 69558737 | 70960589 | 23 |
| **Table S10:** Prioritization of the GWA results of the full Welsh pony cohort based on LD-bound regions (cont.) | | | | | | | | | |
| **Trait** | **Chr** | **Summary** | **Min_SNP** | **Max_SN** | **Sugg_SNPs** | **Sign_SNPs** | **Min_Region** | **Max_Region** | **Total_Genes** |
| **ACTH (cont.)** | 5 | **A** | 16869826 | 17349383 | 30 | 4 | 16534115 | 18234765 | 26 |
|  | 10 | **H** | 55658306 | 56077011 | 2 | 1 | 55060512 | 56255134 | 2 |
|  | 10 | **C H** | 80023665 | NA | 1 | 0 | 79973665 | 80073665 | 6 |
|  | 18 |  | 4529063 | NA | 1 | 0 | 4479063 | 4579063 | 1 |
|  | 19 | **C** | 24243287 | 24246621 | 2 | 0 | 24226051 | 24312494 | 6 |
|  | 19 |  | 33315383 | 33342063 | 4 | 0 | 33290960 | 33465193 | 4 |
|  | 20 |  | 63684506 | NA | 1 | 0 | 63634506 | 63734506 | 1 |
|  | 21 |  | 264658 | 2467359 | 12 | 0 | -566031 | 5646555 | 188 |
|  | 25 |  | 26250218 | NA | 1 | 0 | 26200218 | 26300218 | 5 |
| **NH** | 1 |  | 91537471 | 91969415 | 11 | 0 | 91257387 | 92299877 | 13 |
|  | 4 |  | 62017772 | 62060721 | 2 | 2 | 61306607 | 62232293 | 14 |
|  | 4 | **A F G** | 68114618 | 68576476 | 2 | 0 | 67588953 | 69039376 | 6 |
|  | 4 |  | 77390519 | NA | 1 | 0 | 77340519 | 77440519 | 1 |
|  | 4 |  | 78314683 | 78699729 | 3 | 1 | 77300209 | 79809543 | 26 |
|  | 4 | **A** | 79698145 | 80390074 | 14 | 7 | 77298241 | 81186565 | 40 |
|  | 6 | **F** | 1019810 | 1033178 | 3 | 0 | 903258 | 1451922 | 2 |
|  | 7 | **E** | 93233594 | 93628623 | 10 | 6 | 93176991 | 93628686 | 1 |
|  | 8 | **C** | 64510733 | 64609130 | 2 | 1 | 64277141 | 64856816 | 5 |
|  | 8 |  | 88125499 | 88327659 | 2 | 1 | 87999710 | 88892517 | 5 |
|  | 9 | **H** | 33913440 | 35808721 | 39 | 9 | 32632235 | 37587269 | 18 |
|  | 11 | **E H** | 19050799 | 19240093 | 11 | 8 | 18342117 | 19876247 | 60 |
|  | 12 |  | 7654801 | 7676262 | 2 | 0 | 7314424 | 7786773 | 1 |
|  | 12 |  | 15601877 | NA | 1 | 0 | 15551877 | 15651877 | 1 |
|  | 14 | **H** | 63736228 | 63834285 | 7 | 3 | 63702522 | 63847210 | 2 |
|  | 20 | **H** | 40661395 | 41066022 | 10 | 4 | 40244007 | 41210876 | 14 |
|  | 20 | **H** | 60832063 | 61575820 | 11 | 2 | 60723014 | 61735694 | 2 |
|  | 21 | **A E H** | 20812917 | 22117426 | 22 | 13 | 19515280 | 23543447 | 33 |
|  | 24 |  | 21429112 | 21604747 | 3 | 0 | 20723403 | 21637158 | 13 |
|  | 24 | **H** | 33796794 | 35472785 | 62 | 33 | 31843480 | 36758215 | 57 |
| **Table S10:** Prioritization of the GWA results of the full Welsh pony cohort based on LD-bound regions (cont.) | | | | | | | | | |
| **Trait** | **Chr** | **Summary** | **Min_SNP** | **Max_SN** | **Sugg_SNPs** | **Sign_SNPs** | **Min_Region** | **Max_Region** | **Total_Genes** |
| **NH (cont.)** | 27 |  | 14461113 | 14463955 | 2 | NA | 14185003 | 14663824 | 6 |
|  | 29 |  | 33232105 | 33233161 | 2 | NA | 32443212 | 34147999 | 11 |
| **GH** | 1 | **C** | 120905261 | NA | 1 | 0 | 120855261 | 120955261 | 3 |
|  | 1 | **H** | 132203667 | 133711337 | 30 | 14 | 132184772 | 133716124 | 16 |
|  | 1 |  | 150735268 | NA | 1 | 0 | 150685268 | 150785268 | 1 |
|  | 4 |  | 67153317 | 67163513 | 2 | 0 | 67079978 | 67434976 | 4 |
|  | 4 | **F** | 68576476 | NA | 1 | 0 | 68526476 | 68626476 | 1 |
|  | 4 | **A H** | 84285316 | 85497218 | 63 | 33 | 81804323 | 85719241 | 69 |
|  | 6 | **F** | 1019810 | 1154034 | 4 | 3 | 903258 | 1734708 | 5 |
|  | 7 | **E** | 93233594 | 93580126 | 7 | 4 | 93191676 | 93628672 | 1 |
|  | 9 |  | 55626969 | 55685330 | 2 | 0 | 55565458 | 56169206 | 9 |
|  | 10 |  | 3673095 | 3673552 | 2 | 0 | 3671537 | 3801071 | 0 |
|  | 10 |  | 32529022 | 32559811 | 4 | 0 | 32333094 | 33276546 | 2 |
|  | 11 | **E H** | 18827291 | 19240093 | 14 | 9 | 18613895 | 19317536 | 26 |
|  | 12 |  | 25641997 | NA | 1 | NA | 25591997 | 25691997 | 2 |
|  | 15 |  | 15062753 | 15656836 | 12 | 4 | 13131438 | 16662645 | 55 |
|  | 19 | **H** | 31283482 | 31445588 | 9 | 1 | 31204596 | 31799125 | 0 |
|  | 20 | **A** | 30141925 | 30449510 | 12 | 4 | 29486630 | 30976763 | 62 |
|  | 20 | **H** | 64731849 | 64861251 | 12 | 7 | 64722427 | 65336095 | 4 |
|  | 21 | **E H** | 22007711 | NA | 2 | 0 | 21957711 | 22057711 | 1 |
|  | 22 | **H** | 41033715 | 41065262 | 4 | 4 | 41032889 | 41066045 | 0 |
|  | 24 |  | 18072215 | 18172937 | 4 | 0 | 18024756 | 18508703 | 2 |
|  | 25 | **H** | 19485041 | NA | 1 | 1 | 19435041 | 19535041 | 4 |
|  | 31 |  | 17898824 | 17912707 | 4 | 0 | 17847583 | 18148648 | 2 |
|  | 31 |  | 18700158 | 18819670 | 4 | 0 | 18685559 | 18858145 | 1 |
| **LAM** | 1 | **C H** | 49441032 | NA | 1 | 1 | 49391032 | 49491032 | 1 |
|  | 2 | **A H** | 36123836 | 36633565 | 14 | 11 | 35909634 | 36665473 | 13 |
|  | 5 |  | 79658109 | NA | 1 | 0 | 79608109 | 79708109 | 1 |
|  | 7 |  | 97437120 | 97439429 | 2 | 0 | 97415193 | 97521397 | 0 |
| **Table S10:** Prioritization of the GWA results of the full Welsh pony cohort based on LD-bound regions (cont.) | | | | | | | | | |
| **Trait** | **Chr** | **Summary** | **Min_SNP** | **Max_SN** | **Sugg_SNPs** | **Sign_SNPs** | **Min_Region** | **Max_Region** | **Total_Genes** |
| **LAM (cont.)** | 10 |  | 64224504 | 64281425 | 2 | 0 | 64224315 | 64303766 | 1 |
|  | 13 |  | 27143211 | 27035221 | 5 | 0 | 27081260 | 27274065 | 2 |
|  | 14 |  | 52578019 | 52579053 | 2 | 0 | 52549348 | 52667915 | 2 |
|  | 15 | **C H** | 50978261 | 51005138 | 3 | 1 | 50973563 | 51006110 | 0 |
|  | 15 |  | 64654206 | 64769743 | 12 | 0 | 64265615 | 65947460 | 16 |
|  | 16 |  | 35123235 | 35595543 | 3 | 0 | 35116321 | 35769589 | 6 |
|  | 16 | **C** | 66694166 | NA | 1 | 0 | 66644166 | 66744166 | 1 |
|  | 19 |  | 39125743 | 39626653 | 11 | 1 | 37990377 | 39825664 | 27 |
|  | 19 | **H** | 59885237 | 61849890 | 78 | 35 | 57082025 | 62825378 | 59 |
|  | 20 |  | 62018962 | 62085163 | 4 | 3 | 61971048 | 62085845 | 1 |
|  | 22 | **D** | 3551367 | NA | 1 | 0 | 3501367 | 3601367 | 1 |
|  | 25 |  | 32816803 | 32852556 | 2 | 0 | 32681105 | 32892597 | 7 |
|  | 28 | **H** | 10461982 | 10666731 | 19 | 7 | 9990892 | 10844823 | 4 |
|  | 31 |  | 10611327 | 10509324 | 5 | 3 | 10611124 | 10918134 | 3 |

To be considered an ROI, at least five SNP had to exceed the suggestive threshold (1.0e-05) with one SNP exceeding the threshold for genome-wide significance (5.98e-08). Provided in the table is the base pair position of the lowest (Min_SNP) and highest (Max_SNP) SNP in the region, the min (Min_Region) and maximum (Max_Region) boundaries of the region based on LD, as well as the number of SNPs per region which exceeded the suggestive (Sugg_SNPs) and genome-wide significance (Sign_SNPs) threshold. Total_Genes includes all protein-coding genes, pseudogenes, and RNA genes identified for region based on EquCab3. A black box in the summary column indicates the region did not meet the criteria to be considered an ROI and was not shared with another GWA cohort or trait. Letters in the summary column represent: (A) region was shared with another Welsh pony cohort GWA and at least one region was considered an ROI, (B) region was shared with the Morgan GWA and at least one region was considered an ROI, (C) region was shared with another Welsh pony cohort GWA but no regions met the criteria for an ROI, (D) region was shared with Morgan GWA but no regions met the criteria for an ROI, (E) region was shared with another trait in this GWA cohort and at least one region was considered an ROI, (F) region was shared with another trait in this GWA cohort but no regions met the criteria for an ROI, (G) region was shared across multiple traits in another GWA cohort, (H) region was identified as shared across breeds on metanalysis and was considered a MA-ROI. Highlighted chromosomes (Chr) indicate regions which were shared with several traits.

| **Table S11:** Prioritization of the GWA results of section A, B, C and D Welsh ponies based on the LD-bound regions | | | | | | | | | |
| --- | --- | --- | --- | --- | --- | --- | --- | --- | --- |
| **Trait** | **Chr** | **Summary** | **Min_SNP** | **Max_SNP** | **Sugg_SNPs** | **Sign_SNPs** | **Min_Region** | **Max_Region** | **Total_Genes** |
| **Insulin** | 1 |  | 88317885 | NA | 1 | 0 | 88267885 | 88367885 | 0 |
|  | 4 |  | 16025941 | 16029966 | 4 | 0 | 15938986 | 16180572 | 4 |
|  | 6 | **A** | 82585066 | 83826234 | 32 | 2 | 80572788 | 83599194 | 37 |
|  | 9 | **A** | 61021946 | 61144885 | 13 | 3 | 58372873 | 61241976 | 19 |
|  | 12 |  | 859517 | NA | 1 | 0 | 809517 | 909517 | 2 |
|  | 12 |  | 5811391 | 5812573 | 3 | 0 | 5254445 | 6105807 | 3 |
|  | 17 |  | 11193396 | 11343933 | 4 | 0 | 10851587 | 11442936 | 3 |
|  | 18 | **E** | 79126354 | 79216656 | 3 | 1 | 78720858 | 79634082 | 6 |
|  | 21 |  | 34649027 | NA | 1 | 0 | 34599027 | 34699027 | 0 |
|  | 24 |  | 38174280 | NA | 1 | 0 | 38124280 | 38224280 | 2 |
| **Insulin-OST** | 1 | **E** | 176823704 | NA | 1 | 0 | 176773704 | 176873704 | 1 |
|  | 1 |  | 181205641 | NA | 1 | 0 | 181155641 | 181255641 | 0 |
|  | 3 |  | 67119398 | NA | 1 | 0 | 67069398 | 67169398 | 2 |
|  | 5 |  | 89878763 | NA | 1 | 0 | 89828763 | 89928763 | 2 |
|  | 6 | **C** | 15257536 | 15267456 | 3 | 1 | 15328522 | 15802598 | 5 |
|  | 10 | **B C H** | 73334761 | 73417042 | 3 | 0 | 71967783 | 72438937 | 3 |
|  | 31 |  | 5253579 | NA | 1 | 0 | 5203579 | 5303579 | 2 |
| **Glucose** | 2 |  | 88732913 | 88775982 | 2 | 0 | 87700669 | 88704605 | 7 |
|  | 4 |  | 57433023 | 57463516 | 2 | 1 | 56762597 | 57781825 | 21 |
|  | 8 | **C** | 84774486 | 84918226 | 5 | 0 | 81206774 | 81830595 | 2 |
|  | 8 | **A** | 92368897 | NA | 1 | 0 | 92318897 | 92418897 | 2 |
|  | 15 |  | 71112709 | NA | 1 | 0 | 71062709 | 71162709 | 2 |
|  | 19 |  | 8738837 | 8781089 | 2 | 0 | 6175910 | 6939325 | 4 |
|  | 29 |  | 4401376 | 4855454 | 4 | 1 | 3141136 | 3956266 | 3 |
| **Glucose-OST** | 5 | **C** | 63041759 | 63549216 | 12 | 0 | 64707107 | 68397655 | 30 |
|  | 23 | **C** | 10217644 | 10226427 | 2 | 0 | 10729234 | 11001562 | 0 |
|  | 28 |  | 14969841 | NA | 1 | 0 | 14919841 | 15019841 | 0 |
|  | 28 | **A E G H** | 34960948 | NA | 1 | 1 | 34910948 | 35010948 | 4 |
| **NEFA** | 6 | **C** | 77102911 | NA | 1 | 0 | 77052911 | 77152911 | 1 |
| **Table S11:** Prioritization of the GWA results of section A, B, C and D Welsh ponies based on the LD-bound regions (cont.) | | | | | | | | | |
| **Trait** | **Chr** | **Summary** | **Min_SNP** | **Max_SNP** | **Sugg_SNPs** | **Sign_SNPs** | **Min_Region** | **Max_Region** | **Total_Genes** |
| **NEFA (cont.)** | 7 | **C** | 7744001 | 7858148 | 2 | 0 | 7084283 | 7235090 | 1 |
|  | 7 |  | 8657121 | 8718823 | 3 | 0 | 7794692 | 8979276 | 3 |
|  | 9 | **A** | 48687570 | 50301924 | 74 | 5 | 43402596 | 51140717 | 71 |
|  | 13 |  | 3866723 | NA | 1 | 0 | 3816723 | 3916723 | 2 |
|  | 14 | **C** | 33187338 | NA | 1 | 0 | 33137338 | 33237338 | 1 |
|  | 22 | **C** | 19009107 | NA | 1 | 0 | 18959107 | 19059107 | 1 |
|  | 28 | **A E G** | 34777499 | 35488520 | 25 | 6 | 32909542 | 35703535 | 76 |
|  | 31 |  | 9275456 | 8325401 | 9 | 0 | 9225456 | 9325456 | 2 |
|  | 32 |  | 21391267 | 21497776 | 2 | 0 | 20203883 | 21904415 | 0 |
| **TG** | 7 |  | 28031826 | 28039745 | 2 | 0 | 27130249 | 27583217 | 6 |
|  | 12 | **C** | 35945816 | 35956541 | 2 | 0 | 31936287 | 32490177 | 17 |
|  | 17 |  | 17532266 | NA | 2 | 0 | 17482266 | 17582266 | 2 |
|  | 17 |  | 33912651 | NA | 1 | 0 | 33862651 | 33962651 | 1 |
|  | 20 |  | 56719186 | 56815453 | 4 | 1 | 53870768 | 56991611 | 21 |
| **Adiponectin** | 1 | **E** | 175782149 | 177072407 | 32 | 1 | 171861236 | 178270042 | 49 |
|  | 7 |  | 75100837 | NA | 1 | 0 | 75050837 | 75150837 | 7 |
|  | 20 |  | 8415408 | 9139191 | 5 | 0 | 6054702 | 11308877 | 65 |
|  | 22 | **C** | 37875269 | 37957795 | 3 | 0 | 36564923 | 37148074 | 9 |
|  | 25 |  | 9125953 | NA | 1 | 0 | 9075953 | 9175953 | 1 |
|  | 28 |  | 41052952 | NA | 1 | 0 | 41002952 | 41102952 | 1 |
| **Leptin** | 2 |  | 87434404 | NA | 1 | 0 | 87384404 | 87484404 | 1 |
|  | 4 | **F** | 48014169 | 48031048 | 6 | 0 | 47052514 | 48193459 | 6 |
|  | 6 |  | 2348093 | 2376386 | 6 | 1 | 488137 | 4012580 | 25 |
|  | 6 |  | 21686436 | 22141052 | 9 | 0 | 21323668 | 22378607 | 6 |
|  | 7 | **A H** | 67955613 | 67964668 | 2 | 0 | 65678376 | 65985348 | 2 |
|  | 8 |  | 87412707 | NA | 1 | 0 | 87362707 | 87462707 | 1 |
|  | 10 | **C H** | 857433 | 884264 | 4 | 0 | 809627 | 1068890 | 5 |
|  | 10 |  | 83363991 | NA | 1 | 0 | 83313991 | 83413991 | 1 |
|  | 10 |  | 84395615 | NA | 1 | 0 | 84345615 | 84445615 | 2 |
| **Table S11:** Prioritization of the GWA results of section A, B, C and D Welsh ponies based on the LD-bound regions (cont.) | | | | | | | | | |
| **Trait** | **Chr** | **Summary** | **Min_SNP** | **Max_SNP** | **Sugg_SNPs** | **Sign_SNPs** | **Min_Region** | **Max_Region** | **Total_Genes** |
| **Leptin (cont.)** | 11 |  | 33592865 | NA | 1 | 0 | 33542865 | 33642865 | 7 |
|  | 12 |  | 25937470 | 25914980 | 2 | 0 | 22339448 | 2294098 | 0 |
|  | 13 |  | 7690179 | NA | 1 | 0 | 7640179 | 7740179 | 3 |
|  | 15 |  | 24814175 | 24816607 | 2 | 0 | 23820813 | 24351119 | 3 |
|  | 16 |  | 42665270 | NA | 1 | 0 | 42615270 | 42715270 | 1 |
|  | 26 |  | 11291558 | 11425566 | 4 | 1 | 10695805 | 10988327 | 1 |
|  | 28 |  | 40504716 | NA | 1 | NA | 40454716 | 40554716 | 0 |
| **ACTH** | 1 | **A F** | 44391917 | 44627074 | 13 | 0 | 43064008 | 44872306 | 7 |
|  | 3 | **D** | 44073772 | 44105888 | 8 | 0 | 41547556 | 43484146 | 9 |
|  | 5 | **A** | 17101043 | 17252354 | 9 | 0 | 18357723 | 21333724 | 36 |
|  | 10 | **C H** | 78845710 | NA | 1 | 1 | 78795710 | 78895710 | 3 |
|  | 15 |  | 13711487 | NA | 1 | 0 | 13661487 | 13761487 | 1 |
|  | 19 | **C** | 24243287 | 24246621 | 2 | 0 | 21800444 | 22098695 | 7 |
|  | 19 |  | 37642432 | NA | 1 | 0 | 37592432 | 37692432 | 2 |
|  | 20 | **E** | 60431850 | NA | 3 | 0 | 60381850 | 60481850 | 0 |
|  | 24 | **C** | 39497717 | NA | 1 | 0 | 39447717 | 39547717 | 0 |
|  | 30 |  | 1302176 | 1304866 | 4 | 0 | 962895 | 1567967 | 8 |
| **NH** | 1 | **F** | 44398249 | NA | 1 | 0 | 44348249 | 44448249 | 0 |
|  | 3 |  | 109783963 | NA | 1 | 0 | 109733963 | 109833963 | 1 |
|  | 4 | **A E G** | 69118549 | 69714717 | 17 | 14 | 68425678 | 69636837 | 6 |
|  | 4 |  | 72715285 | 73055056 | 2 | 0 | 72321830 | 72867956 | 2 |
|  | 4 | **C** | 76437287 | 77891737 | 17 | 0 | 73229449 | 79629933 | 56 |
|  | 4 | **E** | 83194842 | 85546563 | 54 | 38 | 83144842 | 83244842 | 1 |
|  | 8 | **C** | 64510733 | 64548459 | 2 | 0 | 60856045 | 61415070 | 6 |
|  | 14 |  | 57777287 | NA | 1 | 0 | 57727287 | 57827287 | 1 |
|  | 15 |  | 44638315 | NA | 1 | 3 | 44588315 | 44688315 | 0 |
|  | 15 |  | 73207370 | 73338081 | 5 | 3 | 73033562 | 73478127 | 3 |
|  | 16 |  | 19756213 | 20195426 | 10 | 0 | 17609338 | 19833772 | 15 |
|  | 16 |  | 26012920 | NA | 1 | 0 | 25962920 | 26062920 | 0 |
| **Table S11:** Prioritization of the GWA results of section A, B, C and D Welsh ponies based on the LD-bound regions (cont.) | | | | | | | | | |
| **Trait** | **Chr** | **Summary** | **Min_SNP** | **Max_SNP** | **Sugg_SNPs** | **Sign_SNPs** | **Min_Region** | **Max_Region** | **Total_Genes** |
| **NH (cont.)** | 17 |  | 36798348 | 36856368 | 9 | 9 | 36704887 | 37121452 | 0 |
|  | 18 |  | 68917978 | NA | 1 | 1 | 68867978 | 68967978 | 1 |
|  | 20 | **F** | 30160893 | NA | 1 | 1 | 30110893 | 30210893 | 1 |
|  | 21 |  | 6664894 | 6835706 | 5 | 2 | 5280993 | 6396786 | 8 |
|  | 21 | **A F H** | 20812917 | 21679286 | 4 | 0 | 20606675 | 21752563 | 9 |
|  | 24 |  | 8561717 | 11119679 | 17 | 0 | 4971965 | 15097475 | 125 |
|  | 24 |  | 30419181 | 30419482 | 2 | 0 | 30330777 | 30578714 | 0 |
|  | 26 |  | 13669849 | NA | 1 | 0 | 13619849 | 13719849 | 0 |
|  | 29 |  | 12571950 | 12620905 | 5 | 0 | 10813364 | 11907852 | 14 |
|  | 30 |  | 30980395 | NA | 1 | 0 | 30930395 | 31030395 | 5 |
| **GH** | 1 |  | 73428660 | 73434597 | 2 | 0 | 72506297 | 72913313 | 1 |
|  | 1 | **C** | 120654637 | 120684527 | 5 | 0 | 119265228 | 119651584 | 9 |
|  | 4 | **F** | 46793329 | 47851529 | 10 | 0 | 47576773 | 47975728 | 4 |
|  | 4 |  | 52253860 | NA | 1 | 0 | 52203860 | 52303860 | 1 |
|  | 4 |  | 61808230 | 62195564 | 3 | 0 | 61460640 | 62542569 | 11 |
|  | 4 | **E** | 69236860 | 69660028 | 5 | 0 | 68425678 | 69636837 | 6 |
|  | 4 |  | 74331018 | 76480368 | 41 | 7 | 70026254 | 81648125 | 95 |
|  | 4 |  | 79807650 | 80390074 | 12 | 8 | 80015281 | 81132522 | 9 |
|  | 4 | **A E H** | 84044345 | 85375688 | 34 | 18 | 82570011 | 86366835 | 75 |
|  | 5 |  | 20984165 | NA | 1 | 0 | 20934165 | 21034165 | 2 |
|  | 9 |  | 75259988 | 75263736 | 2 | 1 | 73067974 | 73332751 | 3 |
|  | 10 |  | 51267938 | NA | 1 | 0 | 51217938 | 51317938 | 1 |
|  | 11 |  | 15497051 | 16248131 | 36 | 26 | 15414337 | 16451463 | 25 |
|  | 13 |  | 1235274 | 531380 | 29 | 19 | 4097503 | 6272661 | 18 |
|  | 14 |  | 5739617 | 6265883 | 11 | 0 | 6039980 | 7464225 | 18 |
|  | 15 |  | 85423316 | NA | 1 | 0 | 85373316 | 85473316 | 2 |
|  | 16 |  | 27103995 | 28231661 | 41 | 11 | 25105634 | 30681811 | 44 |
|  | 16 |  | 88165628 | 88202104 | 5 | 0 | 85453703 | 87408801 | 25 |
|  | 17 |  | 167021 | NA | 1 | 0 | 117021 | 217021 | 2 |
| **Table S11:** Prioritization of the GWA results of section A, B, C and D Welsh ponies based on the LD-bound regions (cont.) | | | | | | | | | |
| **Trait** | **Chr** | **Summary** | **Min_SNP** | **Max_SNP** | **Sugg_SNPs** | **Sign_SNPs** | **Min_Region** | **Max_Region** | **Total_Genes** |
| **GH (cont.)** | 17 |  | 57101997 | NA | 1 | 0 | 57051997 | 57151997 | 2 |
|  | 18 |  | 70918093 | NA | 1 | 0 | 70868093 | 70968093 | 0 |
|  | 18 |  | 75058371 | 76075236 | 26 | 6 | 74790214 | 76353283 | 23 |
|  | 18 | **E** | 80391110 | 81050756 | 27 | 1 | 79527484 | 81467661 | 25 |
|  | 20 | **A F** | 30141925 | 30160893 | 3 | 1 | 28634038 | 30114993 | 64 |
|  | 20 | **E** | 60935600 | 61788330 | 8 | 1 | 59464566 | 61015217 | 3 |
|  | 21 |  | 18238312 | 18263289 | 2 | 0 | 17104955 | 18679882 | 20 |
|  | 21 | **F** | 21539433 | 21542349 | 2 | 0 | 20611963 | 21174919 | 6 |
|  | 22 |  | 43609456 | NA | 1 | 0 | 43559456 | 43659456 | 0 |
|  | 24 |  | 22090203 | 22552582 | 9 | 0 | 21281696 | 23226701 | 36 |
|  | 25 |  | 25896326 | 25897963 | 2 | 0 | 23582383 | 26321736 | 50 |
| **LAM** | 1 | **C H** | 49441032 | NA | 1 | 0 | 49391032 | 49491032 | 1 |
|  | 2 |  | 29737934 | 29777141 | 17 | 7 | 29447761 | 29803535 | 8 |
|  | 2 | **A H** | 36322824 | 36633565 | 8 | 0 | 35880861 | 36496556 | 12 |
|  | 11 |  | 37530491 | 37555597 | 2 | 0 | 36920316 | 37335219 | 15 |
|  | 16 |  | 9221468 | NA | 1 | 0 | 9171468 | 9271468 | 1 |
|  | 17 |  | 46013130 | 46020667 | 3 | 0 | 46036409 | 46209083 | 3 |
|  | 18 |  | 26676637 | NA | 1 | 0 | 26626637 | 26726637 | 1 |
|  | 19 |  | 37272294 | 37328619 | 15 | 3 | 34513667 | 34812456 | 4 |
|  | 20 |  | 48609221 | NA | 1 | 0 | 48559221 | 48659221 | 3 |
|  | 23 |  | 4297964 | 4341498 | 5 | 0 | 3933380 | 4310086 | 5 |
|  | 27 |  | 4129106 | NA | 1 | 0 | 4079106 | 4179106 | 1 |
|  | 30 |  | 12607858 | 13128439 | 15 | 9 | 11660801 | 12736188 | 12 |

To be considered an ROI, at least five SNP had to exceed the suggestive threshold (1.0e-05) with one SNP exceeding the threshold for genome-wide significance (5.98e-08). Provided in the table is the base pair position of the lowest (Min_SNP) and highest (Max_SNP) SNP in the region, the min (Min_Region) and maximum (Max_Region) boundaries of the region based on LD, as well as the number of SNPs per region which exceeded the suggestive (Sugg_SNPs) and genome-wide significance (Sign_SNPs) threshold. Total_Genes includes all protein-coding genes, pseudogenes, and RNA genes based on EquCab3. Black box in the summary column indicates the region did not meet the criteria to be considered an ROI and was not shared with another GWA cohort or trait. Letters in the summary column represent: (A) region was shared with another Welsh pony cohort and at least one region was considered an ROI, (B) region was shared with the Morgans and at least one region was considered an ROI, (C) region was shared with another Welsh pony cohort but no regions met the criteria for an ROI, (D) region was shared with Morgan but no regions met the criteria for an ROI, (E) region was shared with another trait in this cohort and at least one region was considered an ROI, (F) region was shared with another trait in this cohort but no regions met the criteria for an ROI, (G) region was shared across multiple traits in another cohort, (H) region was identified as shared across breeds on metanalysis and was considered a MA-ROI. Highlighted chromosomes (Chr) indicate regions which were shared with several traits.

| **Table S12:** Prioritization of the GWA results of section A and B Welsh ponies based on LD-bound regions | | | | | | | | | |
| --- | --- | --- | --- | --- | --- | --- | --- | --- | --- |
| **Trait** | **Chr** | **Summary** | **Min_SNP** | **Max_SNP** | **Sugg_SNPs** | **Sign_SNPs** | **Min_Region** | **Max_Region** | **Total_Genes** |
| **Insulin** | 4 |  | 4672416 | NA | 1 | 0 | 4622416 | 4722416 | 1 |
|  | 8 | **A E** | 71911322 | 73187367 | 71 | 48 | 69350844 | 75906595 | 55 |
|  | 9 | **A** | 60513208 | 62048722 | 21 | 6 | 60380309 | 63287617 | 19 |
|  | 10 |  | 72007185 | 72019609 | 2 | 0 | 71920915 | 72410422 | 5 |
|  | 11 |  | 20890216 | NA | 1 | 0 | 20840216 | 20940216 | 9 |
|  | 14 |  | 44171754 | NA | 1 | 0 | 44121754 | 44221754 | 2 |
|  | 15 | **A H** | 5760603 | NA | 1 | 0 | 5710603 | 5810603 | 2 |
|  | 16 |  | 85072895 | 85226499 | 2 | 0 | 84940660 | 85543835 | 12 |
|  | 18 |  | 27406051 | 27434219 | 3 | 0 | 27257163 | 27649509 | 10 |
|  | 21 |  | 37151683 | NA | 1 | 0 | 37101683 | 37201683 | 2 |
| **Insulin-OST** | 3 |  | 67827963 | NA | 1 | 0 | 67777963 | 67877963 | 3 |
|  | 6 | **C** | 15257536 | NA | 1 | 0 | 15207536 | 15307536 | 2 |
|  | 8 | **C E** | 73418276 | 73458142 | 3 | 0 | 73173455 | 73699198 | 2 |
|  | 9 |  | 51694853 | 52360209 | 26 | 1 | 51519922 | 52222979 | 2 |
|  | 10 | **B C H** | 73415161 | NA | 1 | 0 | 73365161 | 73465161 | 2 |
|  | 23 |  | 33075103 | NA | 2 | 0 | 33025103 | 33125103 | 1 |
| **Glucose** | 2 |  | 78104573 | NA | 1 | 0 | 78054573 | 78154573 | 0 |
|  | 4 |  | 91598735 | 91632300 | 3 | 0 | 91430847 | 91770165 | 2 |
|  | 5 |  | 60653615 | 61066511 | 3 | 1 | 58829349 | 61396408 | 8 |
|  | 8 | **A** | 89457249 | 90171577 | 19 | 3 | 86176351 | 93189207 | 41 |
|  | 14 |  | 6606837 | 6628311 | 4 | 3 | 6496358 | 6828698 | 4 |
|  | 17 |  | 79020897 | 79997119 | 21 | 4 | 78895931 | 80077759 | 22 |
|  | 29 | **A** | 22523122 | 23217070 | 6 | 5 | 22370951 | 22609138 | 5 |
|  | 31 |  | 7659497 | 7654406 | 3 | 0 | 7278750 | 7741436 | 2 |
| **Glucose-OST** | 5 | **C** | 63447777 | 63549216 | 3 | 0 | 62823019 | 64472035 | 13 |
|  | 16 |  | 86361940 | NA | 1 | 1 | 86311940 | 86411940 | 1 |
|  | 28 | **A E G H** | 34434081 | 34960948 | 5 | 1 | 34271949 | 35138699 | 9 |
| **NEFA** | 5 |  | 17682899 | NA | 1 | 0 | 17632899 | 17732899 | 2 |
|  | 6 |  | 74667806 | 74721945 | 4 | 0 | 74462718 | 75137224 | 42 |
| **Table S12:** Prioritization of the GWA results of section A and B Welsh ponies based on LD-bound regions (cont.) | | | | | | | | | |
| **Trait** | **Chr** | **Summary** | **Min_SNP** | **Max_SNP** | **Sugg_SNPs** | **Sign_SNPs** | **Min_Region** | **Max_Region** | **Total_Genes** |
| **NEFA (cont.)** | 7 |  | 5590146 | NA | 1 | 0 | 5540146 | 5640146 | 5 |
|  | 7 | **C** | 7744001 | NA | 1 | 0 | 7694001 | 7794001 | 1 |
|  | 7 |  | 90384141 | 90387298 | 2 | 0 | 90103889 | 90535803 | 6 |
|  | 9 | **A** | 48876850 | 50428786 | 66 | 10 | 48031329 | 51265805 | 37 |
|  | 14 | **C** | 33144705 | 33289979 | 13 | 0 | 32851327 | 33404045 | 5 |
|  | 18 |  | 21325941 | 22264264 | 2 | 0 | 20477464 | 22315518 | 9 |
|  | 20 |  | 31639261 | NA | 1 | 0 | 31589261 | 31689261 | 2 |
|  | 22 | **C** | 19009107 | 19028315 | 2 | 0 | 17869740 | 19275273 | 17 |
|  | 28 | **A E G** | 34865969 | 34877252 | 7 | 0 | 34727198 | 35029308 | 5 |
| **TG** | 1 |  | 47645272 | NA | 1 | 0 | 47595272 | 47695272 | 0 |
|  | 2 |  | 98328483 | NA | 1 | 0 | 98278483 | 98378483 | 1 |
|  | 4 |  | 88686448 | NA | 1 | 0 | 88636448 | 88736448 | 1 |
|  | 7 |  | 26533379 | 26635921 | 7 | 1 | 26358820 | 26960566 | 21 |
|  | 9 |  | 73409149 | 73438018 | 4 | 0 | 72531878 | 73855746 | 15 |
|  | 20 |  | 56347955 | NA | 1 | 0 | 56297955 | 56397955 | 1 |
| **Adiponectin** | 8 |  | 5894342 | NA | 1 | 0 | 5844342 | 5944342 | 2 |
|  | 18 |  | 39196722 | NA | 1 | 0 | 39146722 | 39246722 | 1 |
|  | 20 |  | 26633993 | NA | 1 | 0 | 26583993 | 26683993 | 1 |
|  | 22 | **C** | 37957795 | NA | 1 | 1 | 37907795 | 38007795 | 1 |
| **Leptin** | 10 | **C H** | 872249 | NA | 1 | 0 | 822249 | 922249 | 1 |
|  | 14 |  | 60295756 | NA | 1 | 0 | 60245756 | 60345756 | 1 |
|  | 17 |  | 5633648 | NA | 1 | 1 | 5583648 | 5683648 | 1 |
| **ACTH** | 1 | **A** | 44284734 | 45133993 | 30 | 1 | 42944403 | 45232767 | 9 |
|  | 5 |  | 17101043 | 17252354 | 4 | 0 | 16484006 | 18102564 | 25 |
|  | 10 | **C H** | 79880592 | 80023665 | 2 | 1 | 79691144 | 80306613 | 25 |
|  | 11 |  | 58160240 | NA | 1 | 0 | 58110240 | 58210240 | 5 |
|  | 20 |  | 8331002 | 8355327 | 7 | 0 | 8092967 | 8599500 | 7 |
|  | 24 | **C** | 38516095 | 39660384 | 23 | 0 | 38361711 | 40099306 | 28 |
| **NH** | 1 |  | 9347701 | NA | 1 | 0 | 9297701 | 9397701 | 1 |
| **Table S12:** Prioritization of the GWA results of section A and B Welsh ponies based on LD-bound regions (cont.) | | | | | | | | | |
| **Trait** | **Chr** | **Summary** | **Min_SNP** | **Max_SNP** | **Sugg_SNPs** | **Sign_SNPs** | **Min_Region** | **Max_Region** | **Total_Genes** |
| **NH (cont.)** | 3 |  | 69624972 | NA | 1 | 0 | 69574972 | 69674972 | 1 |
|  | 4 | **A** | 67618110 | 69482711 | 14 | 3 | 67130904 | 69873296 | 16 |
|  | 10 |  | 10827320 | NA | 1 | 0 | 10777320 | 10877320 | 3 |
|  | 14 |  | 73473354 | NA | 1 | 0 | 73423354 | 73523354 | 0 |
|  | 21 | **F** | 23990259 | 24995726 | 2 | 0 | 23600027 | 25046226 | 16 |
| **GH** | 1 |  | 166271712 | NA | 1 | 0 | 166221712 | 166321712 | 1 |
|  | 10 |  | 11229405 | NA | 1 | 0 | 11179405 | 11279405 | 9 |
|  | 10 |  | 70502635 | 70536766 | 2 | 1 | 69524859 | 70587090 | 6 |
|  | 12 |  | 20064456 | NA | 1 | 0 | 20014456 | 20114456 | 1 |
|  | 17 |  | 27064422 | NA | 1 | 0 | 27014422 | 27114422 | 2 |
|  | 21 | **F** | 23776930 | 23991948 | 5 | 0 | 23171361 | 24411682 | 12 |
|  | 24 |  | 49764166 | NA | 1 | 0 | 49714166 | 49814166 | 0 |
|  | 22 |  | 23930066 | NA | 1 | 0 | 23880066 | 23980066 | 2 |
|  | 25 |  | 15030393 | NA | 1 | 0 | 14980393 | 15080393 | 2 |
| **LAM** | 3 |  | 77977500 | NA | 1 | 0 | 77927500 | 78027500 | 3 |
|  | 8 |  | 45552432 | NA | 1 | 0 | 45502432 | 45602432 | 1 |
|  | 10 |  | 15374259 | 15988198 | 15 | 2 | 14730688 | 16165003 | 66 |
|  | 13 |  | 24882636 | 25740597 | 8 | 2 | 24242621 | 26399066 | 36 |
|  | 14 |  | 58930834 | 59667233 | 4 | 0 | 58326568 | 60185720 | 17 |
|  | 15 | **C H** | 50978261 | 51005138 | 3 | 1 | 50923563 | 51056110 | 0 |
|  | 16 | **C** | 66471008 | 66521264 | 2 | 0 | 66309711 | 66691345 | 5 |
|  | 18 |  | 15365144 | NA | 1 | 0 | 15315144 | 15415144 | 1 |
|  | 19 |  | 54249861 | 54263396 | 4 | 0 | 54026036 | 54315136 | 2 |
|  | 20 |  | 43136147 | 43150142 | 3 | 0 | 43086126 | 43312141 | 9 |

Provided in the table is the base pair position of the lowest (Min_SNP) and highest (Max_SNP) SNP in the region, the min (Min_ROI) and maximum (Max_ROI) boundaries of the region based on LD, as well as the number of SNPs per region which exceeded the suggestive (Sugg_SNPs) and genome-wide significance (Sign_SNPs) threshold. The total number of genes includes all protein-coding genes, pseudogenes, and RNA genes based on EquCab3. Black box in the summary column indicates the region did not meet the criteria to be considered an ROI and was not shared with another GWA cohort or trait. Letters in the summary column represent: (A) region was shared with another Welsh pony cohort GWA and at least one region was considered an ROI, (B) region was shared with the Morgan GWA and at least one region was considered an ROI, (C) region was shared with another Welsh pony cohort GWA but no regions met the criteria for an ROI, (D) region was shared with Morgan GWA but no regions met the criteria for an ROI, (E) region was shared with another trait in this GWA cohort and at least one region was considered an ROI, (F) region was shared with another trait in this GWA cohort but no regions met the criteria for an ROI, (G) region was shared across multiple traits in another GWA cohort, (H) region was identified as shared across breeds on metanalysis and was considered a MA-ROI. Highlighted chromosomes (Chr) indicate regions which were shared with several traits.

| **Table S13:** Final prioritization of the GWA results based on LD-bound regions for the Welsh ponies | | | | | | | | | | | | | | | | | | | | | |  |
| --- | --- | --- | --- | --- | --- | --- | --- | --- | --- | --- | --- | --- | --- | --- | --- | --- | --- | --- | --- | --- | --- | --- |
| **High Priority Region Based on LD in Welsh Ponies** | | | | | | | | | | | | | | | | | | | | | |  |
| **Trait** | | **Chr** | | **Min_Region** | | | **Max_Region** | | | **Protein_Coding** | | | **Pseudogenes** | | | **RNA_Genes** | | | **Total_Genes** | | |  |
| **Insulin** | | 5 | | 35409104 | | | 44806458 | | | 267 | | | 1 | | | 38 | | | 306 | | |  |
|  | | 8 | | 69350844 | | | 75906595 | | | 32 | | | 0 | | | 23 | | | 55 | | |  |
|  | | 15 | | 5748377 | | | 6612684 | | | 0 | | | 0 | | | 1 | | | 1 | | |  |
|  | | 18 | | 78720858 | | | 79634082 | | | 2 | | | 0 | | | 4 | | | 6 | | |  |
|  | | 24 | | 28451012 | | | 29887250 | | | 2 | | | 0 | | | 4 | | | 6 | | |  |
| **Insulin-OST** | | 1 | | 176773704 | | | 176873704 | | | 0 | | | 0 | | | 1 | | | 1 | | |  |
|  | | 8 | | 73173455 | | | 73699198 | | | 1 | | | 0 | | | 1 | | | 2 | | |  |
|  | | 10 | | 71967783 | | | 72438937 | | | 3 | | | 0 | | | 0 | | | 3 | | |  |
|  | | 28 | | 39322188 | | | 39488807 | | | 8 | | | 0 | | | 1 | | | 9 | | |  |
| **Glucose** | | 15 | | 83728178 | | | 83828178 | | | 2 | | | 0 | | | 0 | | | 2 | | |  |
| **Glucose-OST** | | 28 | | 34271949 | | | 35138699 | | | 9 | | | 0 | | | 0 | | | 9 | | |  |
| **Adiponectin** | | 1 | | 171861236 | | | 178270042 | | | 25 | | | 0 | | | 24 | | | 49 | | |  |
|  | | 18 | | 60060215 | | | 61349045 | | | 7 | | | 0 | | | 6 | | | 13 | | |  |
| **Leptin** | | 5 | | 39751797 | | | 50431769 | | | 207 | | | 0 | | | 32 | | | 239 | | |  |
|  | | 6 | | 488137 | | | 4012580 | | | 15 | | | 0 | | | 10 | | | 25 | | |  |
|  | | 7 | | 65678376 | | | 68117086 | | | 1 | | | 0 | | | 2 | | | 3 | | |  |
|  | | 10 | | 692055 | | | 1068890 | | | 0 | | | 0 | | | 5 | | | 5 | | |  |
|  | | 21 | | 22940681 | | | 23516697 | | | 1 | | | 0 | | | 0 | | | 1 | | |  |
| **NEFA** | | 19 | | 1005718 | | | 1105718 | | | 2 | | | 0 | | | 0 | | | 2 | | |  |
|  | | 28 | | 32909542 | | | 35703535 | | | 65 | | | 0 | | | 11 | | | 76 | | |  |
| **ACTH** | | 1 | | 42944403 | | | 45232767 | | | 5 | | | 0 | | | 4 | | | 9 | | |  |
|  | | 1 | | 69558737 | | | 70960589 | | | 7 | | | 0 | | | 16 | | | 23 | | |  |
|  | | 10 | | 55060512 | | | 56255134 | | | 1 | | | 0 | | | 1 | | | 2 | | |  |
|  | | 10 | | 78795710 | | | 80306613 | | | 20 | | | 0 | | | 5 | | | 25 | | |  |
|  | | 20 | | 60381850 | | | 60481850 | | | 0 | | | 0 | | | 0 | | | 0 | | |  |
| **NH** | | 4 | | 67130904 | | | 69873296 | | | 8 | | | 0 | | | 8 | | | 16 | | |  |
|  | | 4 | | 77298241 | | | 81186565 | | | 24 | | | 1 | | | 15 | | | 40 | | |  |
|  | | 4 | | 83144842 | | | 83244842 | | | 1 | | | 0 | | | 0 | | | 1 | | |  |
|  | | 7 | | 93176991 | | | 93628686 | | | 0 | | | 0 | | | 1 | | | 1 | | |  |
| **Table S13:** Final prioritization of the GWA results based on LD-bound regions for the Welsh ponies (cont.) | | | | | | | | | | | | | | | | | | | | | |  |
| **High Priority Region Based on LD Welsh Ponies (cont.)** | | | | | | | | | | | | | | | | | | | | | |  |
| **Trait** | | **Chr** | | **Min_Region** | | | **Max_Region** | | | **Protein_Coding** | | | **Pseudogenes** | | | **RNA_Genes** | | | **Total_Genes** | | |  |
| **NH (cont.)** | | 9 | | 32632235 | | | 37587269 | | | 10 | | | 0 | | | 8 | | | 18 | | |  |
|  | | 11 | | 18342117 | | | 19876247 | | | 55 | | | 1 | | | 4 | | | 60 | | |  |
|  | | 14 | | 63702522 | | | 63847210 | | | 0 | | | 0 | | | 2 | | | 2 | | |  |
|  | | 20 | | 40244007 | | | 41210876 | | | 3 | | | 0 | | | 11 | | | 14 | | |  |
|  | | 20 | | 60723014 | | | 61735694 | | | 0 | | | 0 | | | 2 | | | 2 | | |  |
|  | | 21 | | 5280993 | | | 6396786 | | | 2 | | | 0 | | | 6 | | | 8 | | |  |
|  | | 21 | | 19515280 | | | 25046226 | | | 22 | | | 0 | | | 27 | | | 49 | | |  |
|  | | 24 | | 31843480 | | | 36758215 | | | 47 | | | 0 | | | 10 | | | 57 | | |  |
| **GH** | | 1 | | 132184772 | | | 133716124 | | | 9 | | | 0 | | | 7 | | | 16 | | |  |
|  | | 4 | | 68425678 | | | 69636837 | | | 5 | | | 0 | | | 1 | | | 6 | | |  |
|  | | 4 | | 70026254 | | | 81648125 | | | 49 | | | 1 | | | 45 | | | 95 | | |  |
|  | | 4 | | 82570011 | | | 86366835 | | | 49 | | | 1 | | | 25 | | | 75 | | |  |
|  | | 7 | | 93191676 | | | 93628672 | | | 0 | | | 0 | | | 1 | | | 1 | | |  |
|  | | 11 | | 15414337 | | | 16451463 | | | 24 | | | 0 | | | 1 | | | 25 | | |  |
|  | | 11 | | 18613895 | | | 19317536 | | | 26 | | | 0 | | | 0 | | | 26 | | |  |
|  | | 18 | | 79527484 | | | 81467661 | | | 13 | | | 0 | | | 12 | | | 25 | | |  |
|  | | 19 | | 31204596 | | | 31799125 | | | 0 | | | 0 | | | 0 | | | 0 | | |  |
|  | | 20 | | 29486630 | | | 30976763 | | | 54 | | | 0 | | | 8 | | | 62 | | |  |
|  | | 20 | | 59464566 | | | 61015217 | | | 1 | | | 0 | | | 2 | | | 3 | | |  |
|  | | 20 | | 64722427 | | | 65336095 | | | 1 | | | 0 | | | 3 | | | 4 | | |  |
|  | | 21 | | 20611963 | | | 22057711 | | | 3 | | | 0 | | | 4 | | | 7 | | |  |
|  | | 22 | | 41032889 | | | 41066045 | | | 0 | | | 0 | | | 0 | | | 0 | | |  |
|  | | 25 | | 19435041 | | | 19535041 | | | 4 | | | 0 | | | 0 | | | 4 | | |  |
| **LAM** | | 1 | | 49391032 | | | 49491032 | | | 0 | | | 0 | | | 1 | | | 1 | | |  |
|  | | 2 | | 35880861 | | | 36665473 | | | 8 | | | 0 | | | 6 | | | 14 | | |  |
|  | | 19 | | 57082025 | | | 62825378 | | | 42 | | | 1 | | | 16 | | | 59 | | |  |
|  | | 28 | | 9990892 | | | 10844823 | | | 4 | | | 0 | | | 0 | | | 4 | | |  |
| **Total** | |  | |  | | |  | | | **1146** | | | **6** | | | **415** | | | **1567** | | |  |
|  |  | |  | | |  | | |  | | |  | | |  | | |  | | |  |  |
| **Table S13:** Final prioritization of the GWA results based on LD-bound regions for the Welsh ponies (cont.) | | | | | | | | | | | | | | | | | | | | | |  |
| **Medium Priority Region Based on LD Welsh Ponies** | | | | | | | | | | | | | | | | | | | | | | |
| **Trait** | | **Chr** | | | **Min_Region** | | | **Max_Region** | | | **Protein_Coding** | | | **Pseudogenes** | | | **RNA_Genes** | | | **Total_Genes** | | |
| **Insulin** | | 6 | | | 80572788 | | | 83599194 | | | 17 | | | 0 | | | 20 | | | 37 | | |
|  | | 9 | | | 60380309 | | | 63287617 | | | 7 | | | 0 | | | 12 | | | 19 | | |
|  | | 15 | | | 54076168 | | | 54634446 | | | 2 | | | 0 | | | 3 | | | 5 | | |
|  | | 23 | | | 45940500 | | | 46233500 | | | 0 | | | 0 | | | 2 | | | 2 | | |
| **Insulin-OST** | | 9 | | | 51519922 | | | 52222979 | | | 1 | | | 0 | | | 1 | | | 2 | | |
| **Glucose** | | 8 | | | 86176351 | | | 93189207 | | | 22 | | | 0 | | | 19 | | | 41 | | |
|  | | 17 | | | 78895931 | | | 80077759 | | | 13 | | | 0 | | | 9 | | | 22 | | |
|  | | 29 | | | 22370951 | | | 22764383 | | | 7 | | | 0 | | | 2 | | | 9 | | |
| **Glucose-OST** | | 4 | | | 40143954 | | | 40782593 | | | 5 | | | 0 | | | 4 | | | 9 | | |
| **Leptin** | | 1 | | | 71902092 | | | 78569116 | | | 34 | | | 0 | | | 23 | | | 57 | | |
| **NEFA** | | 9 | | | 43402596 | | | 51140717 | | | 48 | | | 0 | | | 23 | | | 71 | | |
| **TG** | | 7 | | | 26358820 | | | 26960566 | | | 19 | | | 0 | | | 2 | | | 21 | | |
| **ACTH** | | 5 | | | 16534115 | | | 18234765 | | | 22 | | | 0 | | | 4 | | | 26 | | |
| **NH** | | 15 | | | 73033562 | | | 73478127 | | | 1 | | | 0 | | | 2 | | | 3 | | |
|  | | 17 | | | 36704887 | | | 37121452 | | | 0 | | | 0 | | | 0 | | | 0 | | |
| **GH** | | 13 | | | 4097503 | | | 6272661 | | | 8 | | | 0 | | | 10 | | | 18 | | |
|  | | 15 | | | 13131438 | | | 16662645 | | | 42 | | | 1 | | | 12 | | | 55 | | |
|  | | 16 | | | 25105634 | | | 30681811 | | | 31 | | | 1 | | | 12 | | | 44 | | |
|  | | 18 | | | 74790214 | | | 76353283 | | | 21 | | | 0 | | | 2 | | | 23 | | |
| **LAM** | | 2 | | | 29447761 | | | 29803535 | | | 7 | | | 0 | | | 1 | | | 8 | | |
|  | | 10 | | | 14730688 | | | 16165003 | | | 56 | | | 0 | | | 10 | | | 66 | | |
|  | | 13 | | | 24242621 | | | 26399066 | | | 32 | | | 0 | | | 4 | | | 36 | | |
|  | | 19 | | | 34513667 | | | 34812456 | | | 4 | | | 0 | | | 0 | | | 4 | | |
|  | | 19 | | | 37990377 | | | 39825664 | | | 23 | | | 0 | | | 4 | | | 27 | | |
|  | | 30 | | | 11660801 | | | 12736188 | | | 7 | | | 0 | | | 5 | | | 12 | | |
|  | | 31 | | | 10611124 | | | 10918134 | | | 2 | | | 0 | | | 1 | | | 3 | | |
| **Total** | |  | | |  | | |  | | | **431** | | | **2** | | | **187** | | | **620** | | |
|  |  | |  | | |  | | |  | | |  | | |  | | |  | | |  |  |
|  |  | |  | | |  | | |  | | |  | | |  | | |  | | |  |  |
|  |  | |  | | |  | | |  | | |  | | |  | | |  | | |  |  |
| **Table S13:** Final prioritization of the GWA results based on LD-bound regions for the Welsh ponies (cont.) | | | | | | | | | | | | | | | | | | | | | |  |
| **Low Priority Region Based on LD Welsh Ponies** | | | | | | | | | | | | | | | | | | | | | | |
| **Trait** | | **Chr** | | | **Min_Region** | | | **Max_Region** | | | **Protein_Coding** | | | **Pseudogenes** | | | **RNA_Genes** | | | **Total_Genes** | | |
| **Leptin** | | 4 | | | 47052514 | | | 48193459 | | | 3 | | | 0 | | | 3 | | | 6 | | |
| **NH** | | 1 | | | 44348249 | | | 44448249 | | | 0 | | | 0 | | | 0 | | | 0 | | |
|  | | 6 | | | 903258 | | | 1451922 | | | 1 | | | 0 | | | 1 | | | 2 | | |
|  | | 20 | | | 30110893 | | | 30210893 | | | 1 | | | 0 | | | 0 | | | 1 | | |
| **GH** | | 4 | | | 47576773 | | | 47975728 | | | 1 | | | 0 | | | 3 | | | 4 | | |
|  | | 6 | | | 903258 | | | 1734708 | | | 2 | | | 0 | | | 3 | | | 5 | | |
|  | | 21 | | | 23171361 | | | 24411682 | | | 10 | | | 0 | | | 2 | | | 12 | | |
| **Total** | |  | | |  | | |  | | | **18** | | | **0** | | | **12** | | | **30** | | |

Regions were categorized as high priority (regions found on metanalysis OR region was shared with another trait), medium priority (region was an ROI in at least one Welsh pony cohort but was not shared), or low priority (region was shared across traits but region was not an ROI). Final region boundaries of the region were based on LD-ROI and are indicated by the lowest base pair position (Min_ROI) and the highest base pair position (Max_ROI). The total number of genes includes all protein-coding genes, pseudogenes, and RNA genes identified for region based on EquCab3. Shared regions across prioritized traits are indicated by highlighted chromosomes.

| **Table S14:** Prioritization of the GWA results of the Morgan horses based on the LD-bound regions | | | | | | | | | |
| --- | --- | --- | --- | --- | --- | --- | --- | --- | --- |
| **Trait** | **Chr** | **Summary** | **Min_SNP** | **Max_SNP** | **Sugg_SNPs** | **Sign_SNPs** | **Min_Region** | **Max_Region** | **Total_Genes** |
| **Insulin** | 2 |  | 117366086 | 117410894 | 5 | 1 | 117310352 | 117579882 | 4 |
|  | 3 |  | 115316619 | 115326166 | 4 | 0 | 114849263 | 115698498 | 10 |
|  | 4 |  | 97370223 | NA | 1 | 0 | 97370223 | 97420223 | 3 |
|  | 5 |  | 88722709 | NA | 2 | 0 | 88722709 | 88772709 | 1 |
|  | 8 |  | 36946690 | NA | 1 | 0 | 36946690 | 36996690 | 2 |
|  | 8 |  | 62414695 | 62422169 | 3 | 0 | 61953438 | 62651012 | 5 |
|  | 10 |  | 54997568 | 55022644 | 3 | 0 | 54821584 | 55225831 | 0 |
|  | 18 |  | 38197723 | NA | 1 | 0 | 38197723 | 38247723 | 0 |
|  | 19 |  | 20841248 | NA | 1 | 0 | 20841248 | 20891248 | 2 |
|  | 20 |  | 4635861 | 4702640 | 7 | 0 | 4544080 | 5465175 | 12 |
|  | 24 | **H** | 21134897 | NA | 1 | 0 | 21134897 | 21184897 | 1 |
|  | 26 |  | 39653507 | NA | 1 | 0 | 39653507 | 39703507 | 2 |
| **Insulin-OST** | 2 |  | 22468309 | 22541921 | 4 | 1 | 21941652 | 22859290 | 15 |
|  | 2 |  | 51548258 | 51661415 | 7 | 0 | 51173763 | 52005569 | 27 |
|  | 4 | **E** | 28373202 | NA | 1 | 0 | 28373202 | 28423202 | 0 |
|  | 4 |  | 57780431 | 57786154 | 2 | 0 | 57533782 | 57927057 | 9 |
|  | 6 | **E** | 32931767 | 33694226 | 2 | 0 | 32751552 | 34029749 | 22 |
|  | 8 |  | 10116471 | NA | 1 | 0 | 10116471 | 10166471 | 3 |
|  | 10 | **B H** | 71996093 | 73613162 | 50 | 5 | 71666607 | 73534053 | 12 |
|  | 11 | **F** | 18848207 | 19009809 | 7 | 0 | 18355073 | 19629302 | 53 |
|  | 20 |  | 51914168 | NA | 1 | 0 | 51914168 | 51964168 | 1 |
|  | 21 |  | 20781491 | NA | 1 | 0 | 20781491 | 20831491 | 1 |
| **Glucose** | 4 | **E H** | 17981325 | 18477651 | 33 | 11 | 17239374 | 19043831 | 11 |
|  | 8 |  | 11530408 | 12159746 | 5 | 1 | 11193683 | 12404572 | 17 |
|  | 16 |  | 42711571 | NA | 1 | 0 | 42711571 | 42761571 | 1 |
|  | 28 |  | 36615983 | NA | 1 | 0 | 36615983 | 36665983 | 2 |
|  | 29 |  | 9494870 | NA | 1 | 0 | 9494870 | 9544870 | 2 |
|  | 31 |  | 21504871 | NA | 1 | 0 | 21504871 | 21554871 | 0 |
| **Glucose-OST** | 2 |  | 62607747 | NA | 1 | 0 | 62607747 | 62657747 | 1 |
| **Table S14:** Prioritization of the GWA results of the Morgan horses based on the LD-bound regions (cont.) | | | | | | | | | |
| **Trait** | **Chr** | **Summary** | **Min_SNP** | **Max_SNP** | **Sugg_SNPs** | **Sign_SNPs** | **Min_Region** | **Max_Region** | **Total_Genes** |
| **Glucose-OST (cont.)** | 3 | **H** | 56674808 | 58220254 | 85 | 53 | 55746338 | 58085997 | 21 |
|  | 4 | **E H** | 27505119 | 28710128 | 39 | 4 | 26695616 | 29116058 | 9 |
|  | 14 |  | 28998387 | 29000329 | 2 | 0 | 28709052 | 29055844 | 9 |
|  | 25 |  | 18872032 | NA | 1 | 0 | 18872032 | 18922032 | 3 |
|  | 26 |  | 22407530 | 23379414 | 23 | 2 | 21572162 | 23496516 | 5 |
| **NEFA** | 1 |  | 166669064 | 166888483 | 3 | 0 | 166406343 | 167009561 | 6 |
|  | 1 | **H** | 185892360 | 186617146 | 25 | 15 | 184859013 | 187238015 | 41 |
|  | 2 |  | 106012533 | 106052266 | 6 | 1 | 105664825 | 106542344 | 13 |
|  | 7 |  | 86986401 | 87004808 | 3 | 0 | 86924655 | 87232954 | 1 |
|  | 9 |  | 76549280 | 76571642 | 3 | 0 | 75789603 | 77130495 | 17 |
|  | 15 |  | 66056425 | NA | 1 | 0 | 66056425 | 66106425 | 0 |
|  | 17 |  | 13427110 | 14189583 | 14 | 1 | 12653835 | 14464765 | 6 |
|  | 18 |  | 7685942 | 9565563 | 44 | 0 | 8293585 | 9790956 | 13 |
|  | 19 |  | 48235446 | NA | 1 | 0 | 48235446 | 48285446 | 2 |
|  | 24 | **H** | 20381260 | 20888104 | 2 | 1 | 20287835 | 20973401 | 16 |
|  | 24 |  | 45325106 | 45675218 | 5 | 0 | 44139172 | 47064880 | 72 |
|  | 30 |  | 6239856 | 6258423 | 5 | 0 | 5851204 | 6743672 | 9 |
|  | 30 | **H** | 20974703 | 21044590 | 11 | 4 | 20915473 | 21380977 | 0 |
| **TG** | 1 |  | 126407798 | 127401777 | 6 | 0 | 126542590 | 128810519 | 46 |
|  | 10 |  | 65383517 | NA | 1 | 0 | 65383517 | 65433517 | 1 |
|  | 20 |  | 52368013 | 52589211 | 4 | 1 | 52145954 | 52997964 | 5 |
|  | 21 | **F** | 49201984 | 49202284 | 2 | 0 | 48839667 | 49489807 | 2 |
| **Adiponectin** | 1 |  | 129650721 | 129653375 | 2 | 0 | 129419765 | 130122651 | 9 |
|  | 1 |  | 138037003 | NA | 1 | 0 | 138037003 | 138087003 | 1 |
|  | 2 | **H** | 16747148 | 17739125 | 38 | 27 | 16362904 | 18105119 | 42 |
|  | 4 | **H** | 36557672 | 38544490 | 54 | 4 | 34723398 | 39321960 | 47 |
|  | 6 | **E H** | 32601529 | 32727370 | 19 | 1 | 32486287 | 32841880 | 7 |
|  | 6 | **E H** | 67997807 | 69847785 | 68 | 6 | 64297403 | 71493047 | 191 |
|  | 7 |  | 21524454 | 21986901 | 14 | 0 | 19621101 | 22583950 | 53 |
| **Table S14:** Prioritization of the GWA results of the Morgan horses based on the LD-bound regions (cont.) | | | | | | | | | |
| **Trait** | **Chr** | **Summary** | **Min_SNP** | **Max_SNP** | **Sugg_SNPs** | **Sign_SNPs** | **Min_Region** | **Max_Region** | **Total_Genes** |
| **Adiponectin (cont.)** | 7 |  | 32963159 | 32963459 | 2 | 0 | 32448807 | 33202795 | 29 |
|  | 8 |  | 3347264 | 3419299 | 6 | 0 | 2972877 | 3485969 | 18 |
|  | 15 |  | 21830373 | 21834175 | 2 | 0 | 21702151 | 21904600 | 1 |
|  | 15 |  | 66865469 | 66893151 | 4 | 0 | 66810113 | 66986537 | 2 |
|  | 18 | **F H** | 41448414 | NA | 1 | 1 | 41448414 | 41498414 | 1 |
|  | 18 |  | 49705278 | 49893633 | 7 | 0 | 48222088 | 50189162 | 36 |
|  | 19 |  | 25833383 | 25859655 | 2 | 0 | 25269042 | 26285152 | 16 |
|  | 20 | **H** | 3734902 | 3954772 | 12 | 0 | 3649052 | 4325872 | 11 |
|  | 20 |  | 1882774 | NA | 1 | 0 | 1882774 | 1932774 | 2 |
|  | 21 | **F** | 49478363 | NA | 1 | 0 | 49478363 | 49528363 | 1 |
| **Leptin** | 1 |  | 130957068 | 131062691 | 3 | 0 | 130419659 | 131677667 | 14 |
|  | 4 | **E** | 52373692 | 52614368 | 22 | 0 | 51590680 | 52810437 | 9 |
|  | 6 |  | 38446793 | NA | 1 | 0 | 38446793 | 38496793 | 3 |
|  | 8 |  | 8682147 | NA | 1 | 0 | 8682147 | 8732147 | 2 |
|  | 19 | **H** | 51360775 | 53132722 | 57 | 27 | 51286493 | 53959028 | 21 |
|  | 21 |  | 16547954 | 16608200 | 3 | 0 | 14655783 | 16880737 | 21 |
|  | 24 | **H** | 27275709 | 29038412 | 65 | 14 | 25564765 | 29384679 | 21 |
|  | 25 |  | 27438558 | 27907420 | 14 | 2 | 26217071 | 29045128 | 65 |
| **ACTH** | 1 | **E H** | 83546191 | 83734040 | 17 | 4 | 82700933 | 84269783 | 24 |
|  | 3 | **D H** | 43335201 | 44116411 | 13 | 0 | 42674448 | 44422013 | 10 |
|  | 3 | **H** | 103056163 | 103438726 | 49 | 34 | 102944842 | 103801021 | 6 |
|  | 5 | **H** | 25785666 | 27061038 | 32 | 10 | 25378878 | 27689002 | 28 |
|  | 10 |  | 67992633 | 67997136 | 2 | 0 | 67173693 | 68509748 | 16 |
|  | 10 |  | 70528773 | NA | 1 | 0 | 70528773 | 70578773 | 0 |
|  | 11 | **F** | 18728679 | 18904099 | 4 | 0 | 17711712 | 19910206 | 80 |
|  | 11 |  | 52897545 | 53669056 | 32 | 0 | 52809863 | 54320401 | 18 |
|  | 13 |  | 25806289 | NA | 1 | 0 | 25806289 | 25856289 | 3 |
|  | 16 |  | 31200001 | NA | 1 | 0 | 31200001 | 31250001 | 1 |
|  | 18 | **F** | 41392781 | NA | 1 | 0 | 41392781 | 41442781 | 1 |
| **Table S14:** Prioritization of the GWA results of the Morgan horses based on the LD-bound regions (cont.) | | | | | | | | | |
| **Trait** | **Chr** | **Summary** | **Min_SNP** | **Max_SNP** | **Sugg_SNPs** | **Sign_SNPs** | **Min_Region** | **Max_Region** | **Total_Genes** |
| **ACTH (cont.)** | 20 |  | 29056288 | NA | 1 | 0 | 29056288 | 29106288 | 3 |
|  | 21 |  | 11112604 | NA | 1 | 0 | 11112604 | 11162604 | 0 |
|  | 21 |  | 24436227 | 24439739 | 3 | 0 | 23458912 | 25104737 | 16 |
|  | 25 |  | 13299542 | NA | 1 | 0 | 13299542 | 13349542 | 0 |
|  | 25 |  | 14989527 | NA | 1 | 0 | 14989527 | 15039527 | 1 |
|  | 31 |  | 16965044 | 17737242 | 4 | 0 | 16852976 | 17943693 | 21 |
| **NH** | 1 | **F** | 78493587 | 79782621 | 37 | 0 | 78152399 | 80485573 | 21 |
|  | 1 | **E** | 82958480 | 83232130 | 10 | 0 | 82097718 | 83618523 | 20 |
|  | 2 |  | 93824111 | 93833011 | 2 | 0 | 93612698 | 93999072 | 2 |
|  | 4 | **E H** | 52076906 | 53659651 | 149 | 110 | 52024470 | 54237747 | 20 |
|  | 5 |  | 59796357 | 60233277 | 10 | 0 | 59986780 | 60283685 | 1 |
|  | 5 |  | 65804297 | 65824216 | 3 | 0 | 65300990 | 66750795 | 4 |
|  | 6 | **E H** | 64502443 | 65350057 | 44 | 12 | 60410647 | 70570773 | 172 |
|  | 8 |  | 29756282 | NA | 1 | 0 | 29756282 | 29806282 | 0 |
|  | 9 |  | 49062306 | 49078134 | 2 | 0 | 47678054 | 55125332 | 61 |
|  | 14 |  | 74532493 | NA | 1 | 0 | 74532493 | 74582493 | 1 |
|  | 18 |  | 2306238 | NA | 1 | 0 | 2306238 | 2356238 | 0 |
|  | 19 | **H** | 1188889 | 1197320 | 3 | 2 | 661978 | 1345372 | 6 |
|  | 19 | **E H** | 34421059 | 36247260 | 102 | 23 | 32962795 | 37391949 | 73 |
|  | 19 |  | 46479290 | 47156982 | 5 | 1 | 46345791 | 47243745 | 16 |
|  | 21 |  | 4745903 | NA | 1 | 0 | 4745903 | 4795903 | 0 |
|  | 24 |  | 42026470 | 42450741 | 9 | 1 | 41516893 | 42504006 | 13 |
| **GH** | 1 | **F** | 79175507 | 79234421 | 5 | 0 | 79092549 | 79839480 | 7 |
|  | 1 |  | 109778420 | 109819993 | 4 | 0 | 108645695 | 110793330 | 27 |
|  | 1 | **B H** | 122383349 | 123036781 | 71 | 31 | 120644115 | 124691346 | 56 |
|  | 2 |  | 85183513 | 86093522 | 20 | 10 | 84295572 | 88599903 | 36 |
|  | 4 |  | 3032922 | NA | 1 | 1 | 3032922 | 3082922 | 1 |
|  | 6 | **F** | 3139850 | 3453652 | 2 | 0 | 2318331 | 3601991 | 10 |
|  | 6 |  | 6272129 | 6335115 | 8 | 0 | 6143412 | 6435255 | 8 |
| **Table S14:** Prioritization of the GWA results of the Morgan horses based on the LD-bound regions (cont.) | | | | | | | | | |
| **Trait** | **Chr** | **Summary** | **Min_SNP** | **Max_SNP** | **Sugg_SNPs** | **Sign_SNPs** | **Min_Region** | **Max_Region** | **Total_Genes** |
| **GH (cont.)** | 6 |  | 15729023 | 16202020 | 8 | 0 | 14200808 | 18578117 | 55 |
|  | 7 |  | 26684853 | 26701040 | 6 | 0 | 26591740 | 26974624 | 18 |
|  | 8 |  | 63557829 | NA | 1 | 0 | 63557829 | 63607829 | 0 |
|  | 17 | **H** | 32020513 | 33031579 | 39 | 2 | 31806060 | 33720086 | 7 |
|  | 18 |  | 2423391 | NA | 1 | 0 | 2423391 | 2473391 | 1 |
|  | 22 |  | 45719751 | 48733979 | 2 | 0 | 48299638 | 49204093 | 12 |
|  | 29 |  | 19108245 | 19432974 | 7 | 2 | 19255639 | 19488768 | 3 |
| **LAM** | 2 |  | 66192812 | NA | 1 | 1 | 66192812 | 66242812 | 1 |
|  | 3 |  | 3294278 | NA | 1 | 0 | 3294278 | 3344278 | 2 |
|  | 4 | **E H** | 17509325 | 19295909 | 52 | 4 | 17301415 | 19812653 | 16 |
|  | 6 | **F** | 3466933 | NA | 1 | 0 | 3466933 | 3516933 | 1 |
|  | 6 |  | 79661858 | NA | 1 | 0 | 79661858 | 79711858 | 1 |
|  | 8 |  | 59199626 | 60121756 | 24 | 0 | 59149588 | 60266527 | 9 |
|  | 12 | **H** | 33127411 | 34414133 | 53 | 27 | 32885278 | 34800986 | 45 |
|  | 14 |  | 66311023 | 66688404 | 15 | 0 | 65014422 | 67256851 | 8 |
|  | 14 | **H** | 88975206 | 90135630 | 48 | 9 | 87916190 | 91602875 | 58 |
|  | 16 |  | 64556111 | NA | 1 | 0 | 64556111 | 64606111 | 0 |
|  | 16 |  | 74667638 | NA | 1 | 0 | 74667638 | 74717638 | 2 |
|  | 18 | **H** | 31710749 | 33317633 | 65 | 33 | 30095266 | 35177011 | 36 |
|  | 19 | **H** | 30133826 | NA | 51 | 3 | 30133826 | 30183826 | 2 |
|  | 22 | **B H** | 3616445 | 4853827 | 75 | 45 | 2843476 | 5225020 | 23 |
|  | 22 |  | 13852015 | NA | 1 | 0 | 13852015 | 13902015 | 0 |
|  | 22 |  | 23806850 | NA | 1 | 0 | 23806850 | 23856850 | 1 |
|  | 23 | **H** | 11116499 | 12515439 | 51 | 46 | 7656404 | 12984095 | 34 |
|  | 31 |  | 6804894 | NA | 1 | 0 | 6804894 | 6854894 | 1 |

Provided in the table is the base pair position of the lowest (Min_SNP) and highest (Max_SNP) SNP in the region, the min (Min_Region) and maximum (Max_Region) boundaries of the region based on LD, as well as the number of SNPs per region which exceeded the suggestive (Sugg_SNPs) and genome-wide significance (Sign_SNPs) threshold. The total number of genes includes all protein-coding genes, pseudogenes, and RNA genes based on EquCab3. Black box in the summary column indicates the region did not meet the criteria to be considered an ROI, was not significant on metanalysis and was not shared with another or trait. Letters in the summary column represent: (B) region was shared with one or more Welsh pony cohorts and at least one region was considered an ROI, (D) region was shared with one or more Welsh pony cohorts but no regions met the criteria for an ROI, (E) region was shared with another trait in the Morgan horses and at least one region was considered an ROI, (F) region was shared with another trait in the Morgan horses but no regions met the criteria for an ROI, (H) region was identified as shared across breeds on metanalysis and was considered an MA-ROI. Highlighted chromosomes (Chr) indicate regions which were shared with several traits.

| **Table S15:** Final prioritization of the GWA results based on LD-bound region for the Morgan horses | | | | | | | |
| --- | --- | --- | --- | --- | --- | --- | --- |
| **High Priority Regions based on LD Morgan Horses** | | | | | | | |
| **Trait** | **Chr** | **Min_Region** | **Max_Region** | **Protein_Coding** | **Pseudogenes** | **RNA_Genes** | **Total_Genes** |
| **Insulin** | 24 | 21134897 | 21184897 | 1 | 0 | 0 | 1 |
| **Insulin-OST** | 4 | 28373202 | 28423202 | 0 | 0 | 0 | 0 |
|  | 6 | 32751552 | 34029749 | 12 | 0 | 10 | 22 |
|  | 10 | 71666607 | 73534053 | 6 | 1 | 5 | 12 |
| **Glucose** | 4 | 17239374 | 19043831 | 6 | 0 | 5 | 11 |
|  | 8 | 11193683 | 12404572 | 8 | 0 | 9 | 17 |
| **Glucose-OST** | 3 | 55746338 | 58085997 | 15 | 0 | 6 | 21 |
|  | 4 | 26695616 | 29116058 | 5 | 0 | 4 | 9 |
| **NEFA** | 1 | 184859013 | 187238015 | 24 | 0 | 17 | 41 |
|  | 17 | 12653835 | 14464765 | 4 | 0 | 2 | 6 |
|  | 24 | 20287835 | 20973401 | 15 | 0 | 1 | 16 |
|  | 30 | 20915473 | 21380977 | 0 | 0 | 0 | 0 |
| **Adiponectin** | 2 | 16362904 | 18105119 | 21 | 0 | 21 | 42 |
|  | 4 | 34723398 | 39321960 | 36 | 1 | 10 | 47 |
|  | 6 | 32486287 | 32841880 | 3 | 0 | 4 | 7 |
|  | 6 | 64297403 | 71493047 | 168 | 1 | 22 | 191 |
|  | 18 | 41448414 | 41498414 | 0 | 0 | 1 | 1 |
|  | 20 | 3649052 | 4325872 | 8 | 0 | 3 | 11 |
| **Leptin** | 4 | 51590680 | 52810437 | 4 | 0 | 5 | 9 |
|  | 19 | 51286493 | 53959028 | 7 | 0 | 14 | 21 |
|  | 24 | 25564765 | 29384679 | 7 | 0 | 14 | 21 |
| **ACTH** | 1 | 82700933 | 84269783 | 18 | 1 | 5 | 24 |
|  | 3 | 42674448 | 44422013 | 2 | 1 | 7 | 10 |
|  | 3 | 102944842 | 103801021 | 2 | 0 | 4 | 6 |
|  | 5 | 25378878 | 27689002 | 12 | 0 | 16 | 28 |
| **NH** | 1 | 82097718 | 83618523 | 14 | 0 | 6 | 20 |
|  | 4 | 52024470 | 54237747 | 8 | 0 | 12 | 20 |
|  | 6 | 60410647 | 70570773 | 144 | 2 | 26 | 172 |
| **Table S15:** Final prioritization of the GWA results based on LD-bound region for the Morgan horses (cont.) | | | | | | | |
| **High Priority Regions based on LD Morgan Horses (cont.)** | | | | | | | |
| **Trait** | **Chr** | **Min_Region** | **Max_Region** | **Protein_Coding** | **Pseudogenes** | **RNA_Genes** | **Total_Genes** |
| **NH (cont.)** | 19 | 661978 | 1345372 | 4 | 1 | 1 | 6 |
|  | 19 | 32962795 | 37391949 | 53 | 1 | 19 | 73 |
| **GH** | 1 | 120644115 | 124691346 | 36 | 0 | 20 | 56 |
|  | 17 | 31806060 | 33720086 | 3 | 1 | 3 | 7 |
| **LAM** | 4 | 17301415 | 19812653 | 8 | 1 | 7 | 16 |
|  | 12 | 32885278 | 34800986 | 29 | 0 | 16 | 45 |
|  | 14 | 87916190 | 91602875 | 32 | 0 | 26 | 58 |
|  | 18 | 30095266 | 35177011 | 23 | 0 | 13 | 36 |
|  | 19 | 30133826 | 30183826 | 2 | 0 | 0 | 2 |
|  | 22 | 2843476 | 5225020 | 13 | 0 | 10 | 23 |
|  | 23 | 7656404 | 12984095 | 11 | 0 | 23 | 34 |
| **Total** |  |  |  | **764** | **11** | **367** | **1142** |
|  |  |  |  |  |  |  |  |
| **Medium Priority Regions based on LD Morgan Horses** | | | | | | | |
| **Trait** | **Chr** | **Min_Region** | **Max_Region** | **Protein_Coding** | **Pseudogenes** | **RNA_Genes** | **Total_Genes** |
| **Insulin** | 2 | 117310352 | 117579882 | 2 | 0 | 2 | 4 |
| **Glucose_OST** | 26 | 21572162 | 23496516 | 0 | 0 | 5 | 5 |
| **NEFA** | 2 | 105664825 | 106542344 | 10 | 0 | 3 | 13 |
| **Leptin** | 25 | 26217071 | 29045128 | 59 | 2 | 4 | 65 |
| **NH** | 19 | 46345791 | 47243745 | 15 | 0 | 1 | 16 |
|  | 24 | 41516893 | 42504006 | 8 | 0 | 5 | 13 |
| **GH** | 2 | 84295572 | 88599903 | 22 | 0 | 14 | 36 |
|  | 29 | 19255639 | 19488768 | 3 | 0 | 0 | 3 |
| **Total** |  |  |  | **119** | **2** | **34** | **155** |
|  |  |  |  |  |  |  |  |
| **Low Priority Regions based on LD Morgan Horses** | | | | | | | |
| **Trait** | **Chr** | **Min_Region** | **Max_Region** | **Protein_Coding** | **Pseudogenes** | **RNA_Genes** | **Total_Genes** |
| **Insulin_OST** | 11 | 18355073 | 19629302 | 50 | 1 | 2 | 53 |
| **TG** | 21 | 48839667 | 49489807 | 1 | 0 | 1 | 2 |
| **Table S15:** Final prioritization of the GWA results based on LD-bound region for the Morgan horses (cont.) | | | | | | | |
| **Low Priority Regions based on LD Morgan Horses (cont.)** | | | | | | | |
| **Trait** | **Chr** | **Min_Region** | **Max_Region** | **Protein_Coding** | **Pseudogenes** | **RNA_Genes** | **Total_Genes** |
| **Adiponectin** | 21 | 49478363 | 49528363 | 1 | 0 | 0 | 1 |
| **ACTH** | 11 | 17711712 | 19910206 | 67 | 1 | 12 | 80 |
|  | 18 | 41392781 | 41442781 | 0 | 0 | 1 | 1 |
| **NH** | 1 | 78152399 | 80485573 | 9 | 0 | 12 | 21 |
| **GH** | 1 | 79092549 | 79839480 | 2 | 0 | 5 | 7 |
|  | 6 | 2318331 | 3601991 | 5 | 0 | 5 | 10 |
| **LAM** | 6 | 3466933 | 3516933 | 0 | 0 | 1 | 1 |
| **Total** |  |  |  | **135** | **2** | **39** | **176** |

Regions were categorized as high priority (regions found on metanalysis OR region was shared with another trait), medium priority (region was an ROI in the Morgan horses but was not shared), or low priority (region was shared across traits but region was not an ROI). Final region boundaries of the region were based on LD-ROI and are indicated by the lowest base pair position (Min_ROI) and the highest base pair position (Max_ROI). The total number of genes includes all protein-coding genes, pseudogenes, and RNA genes identified for region based on EquCab3. Shared regions across prioritized traits are indicated by highlighted chromosomes.
