# Supplementary material for: Genome-Wide Association Analyses of Equine Metabolic Syndrome Phenotypes in Welsh Ponies and Morgan Horses
Source: Genes (Basel). 2019 Nov 6;10(11):893. doi: 10.3390/genes10110893 (PMC6895807; doi:10.3390/genes10110893)
Supplement: Supplementary file 1 [file genes-10-00893-s001.zip › Norton_EMS_GWA_Supplementary_Figure.docx]

**
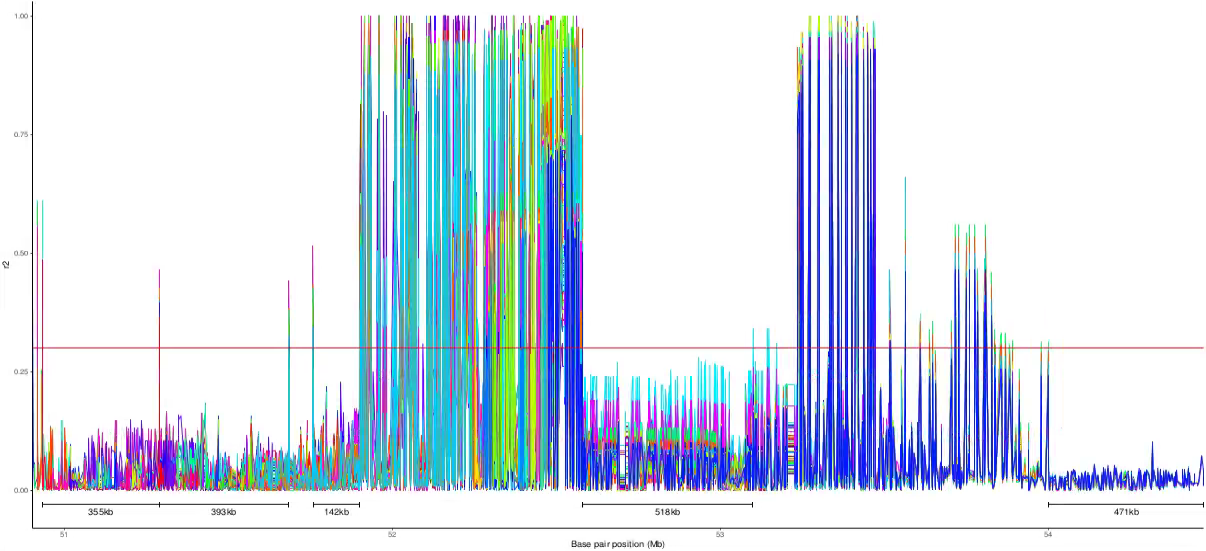
**

**Figure S1:** **Linkage disequilibrium (LD) for neck-to-height-ratio (NH) on equine chromosome 4 (ECA4) in the Morgan horses.** Base pair (bp) positions are on the x-axis and values for the pairwise comparisons of LD (r2) are on the y-axis. Red horizontal line indicates the threshold for LD at an r2 of 0.3. Individual colors represent the LD for each SNP identified on genome wide association analysis. The length in bp of the regions where the LD dropped below 0.3 for a minimum of 100kg for all SNPs are labeled parallel to the x-axis. Two peaks were identified with the first between bp 51900767-52580849 and the second peak from bp 53099275 to 54002853. Evaluation of the reference region identified an inversion at the position of the second peak.
